# Supplementary material for: Pan-microalgal dark proteome mapping via interpretable deep learning and synthetic chimeras
Source: Patterns (N Y). 2025 Sep 24;6(11):101373. doi: 10.1016/j.patter.2025.101373 (PMC12664985; doi:10.1016/j.patter.2025.101373)
Supplement: Document S1. Figures S1–S7 [file mmc1.pdf]

Patterns, Volume *6*

Supplemental information

Pan-microalgal dark proteome mapping via interpretable deep learning and synthetic chimeras

David R. Nelson, Ashish Kumar Jaiswal, Noha Samir Ismail, Alexandra Mystikou, and Kourosh Salehi-Ashtiani

Supplemental information for: "*Pan-microalgal dark proteome mapping via interpretable deep learning and synthetic chimeras*"

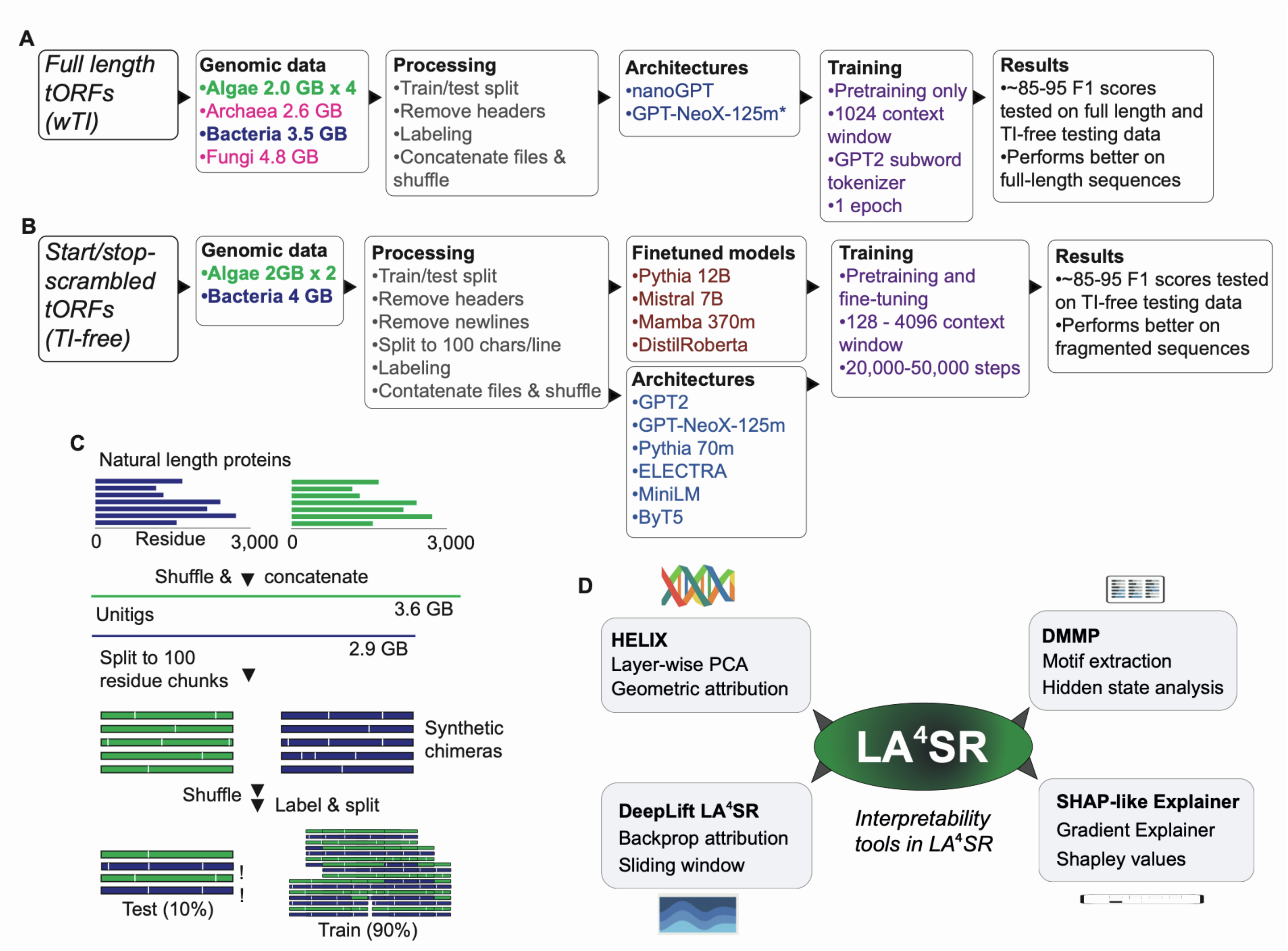

Figure S1. Schematic diagram of LM approaches for DNN embedding and representation of microbial genome-based amino acid sequence data

(A) Integrated, TI-inclusive approach. The TI-inclusive training regime used full-length protein sequences. The algaGPTnano and GPT-NeoX models were trained with a diverse representation of contaminant species' sequences (i.e, full-length proteins, full-spectrum (bacterial, fungal, archaeal), see Data S1). We used all available nonredundant microalgal genomic sequences passing a contamination filter, equaling about 2 gigabytes in total (Table S1).

(B) Core, TI-free approach. This approach had a directive to resolve intermediate regions regardless of TI. All input sequences per class were concatenated to a single line, then divided to lines of 100 residues each and shuffled. Resultant models exhibited robust performance with multiple adaptability and accessibility features, including small memory overhead and strong generalization capacity on incomplete sequences. The training data and scripts are in Data S1 and Data S2.

(C) Illustration of the TI-free set preparation.

(D) Interpretability tools for protein language models (PLMs). These models were developed to decipher the influence of amino acid patterns in the model's decision-making process at different scales.

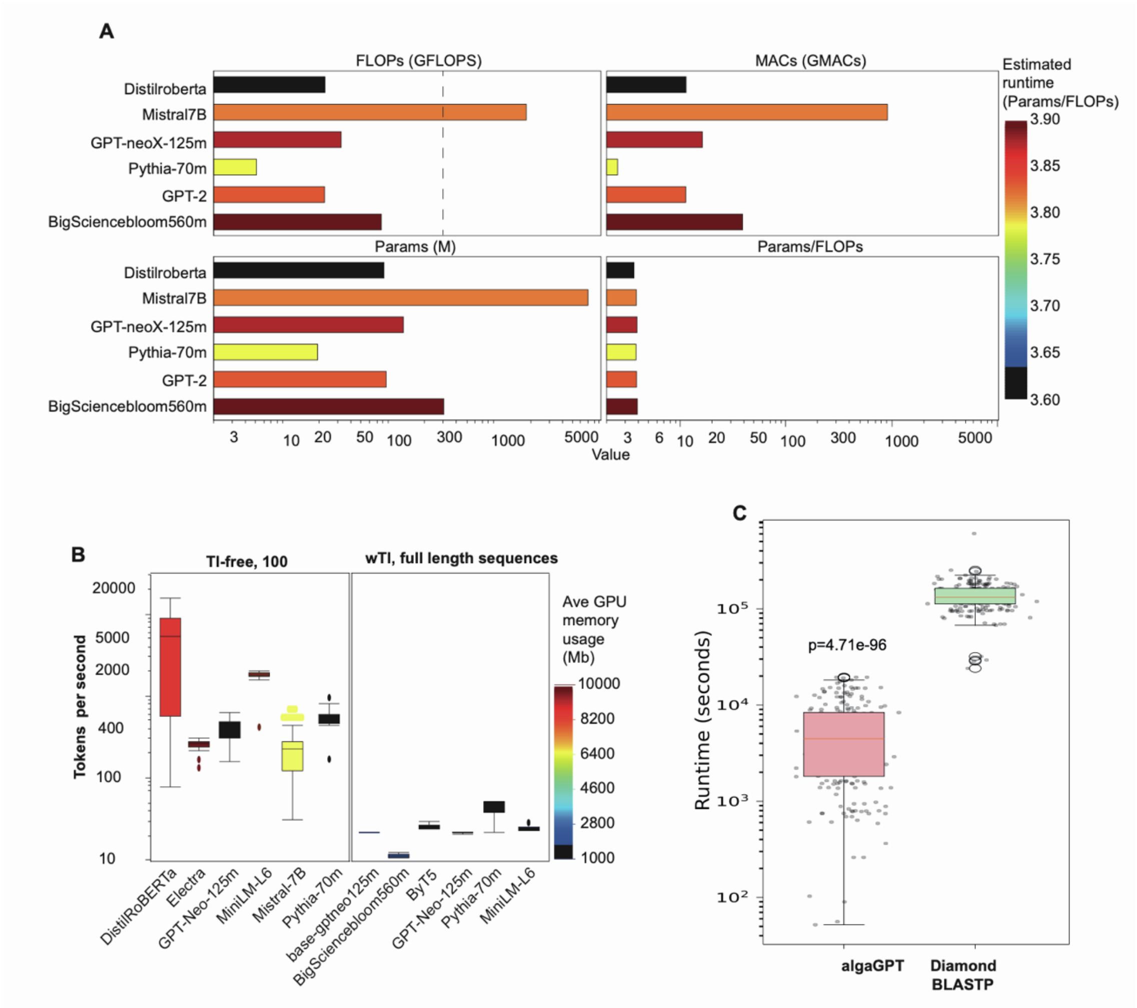

Figure S2. Technical performance evaluation of representative open-source LM model architectures used in LA⁴SR

(A) Comparison of FLOPS, MACs, and parameters of representative LA⁴SR models.
(B) The wTI and TI-free approaches are compared in terms of tokens per second and average GPU usage during inference of either TI-free (100 amino acids per query) and wTI (i.e., full-length sequences, average length = 197.76). We developed a custom evaluation pipeline that could accommodate a range of models. Results from compatible architectures are shown.
(C) Runtimes estimates of classification models. The algaGPT model runs 82.9x faster, on average, than Diamond BLASTP[S1], which has been shown to have up to 20,000 the speed of BLASTX (also see Table S1).

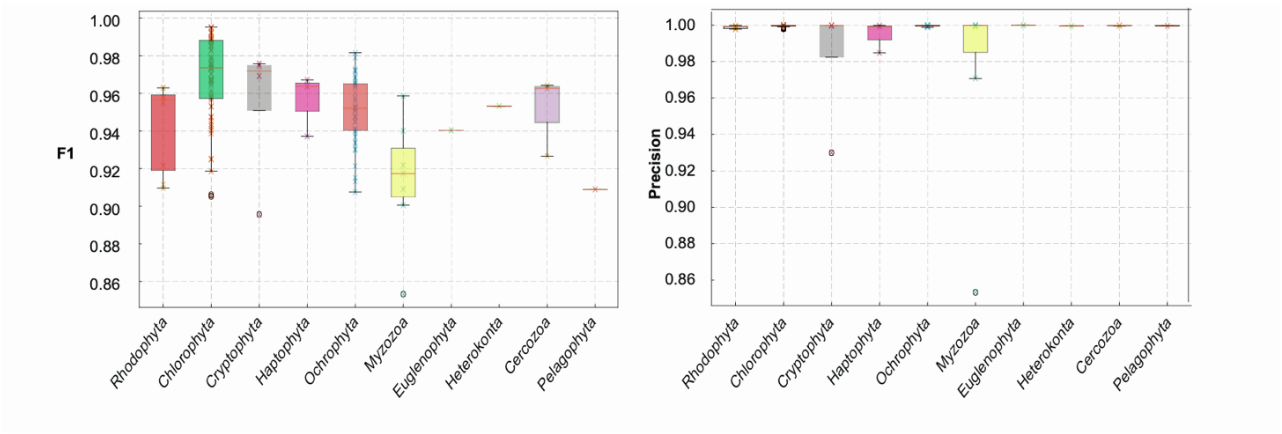

Figure S3. Microalgal lineage-stratified sensitivity analysis of LA⁴SR classification performance.

Per-class F_1 and precision metrics were computed using the TI-free LA⁴SR 13m model on full-length hold-out in silico proteomes. Boxplots depict the median (horizontal line), interquartile range (box), whiskers extending to 1.5× the interquartile range, and individual lineage scores as overlaid points. Despite uneven representation (e.g., very small clades or genera), median precision remains ≥ 0.98 across all groupings, confirming robust generalizability. F_1 score distributions aggregated by phylum across 10 microalgal lineages. Variation reflects differential sample representation and evolutionary distance; enforcing equal-size stratified sampling severely degraded performance in all phyla.

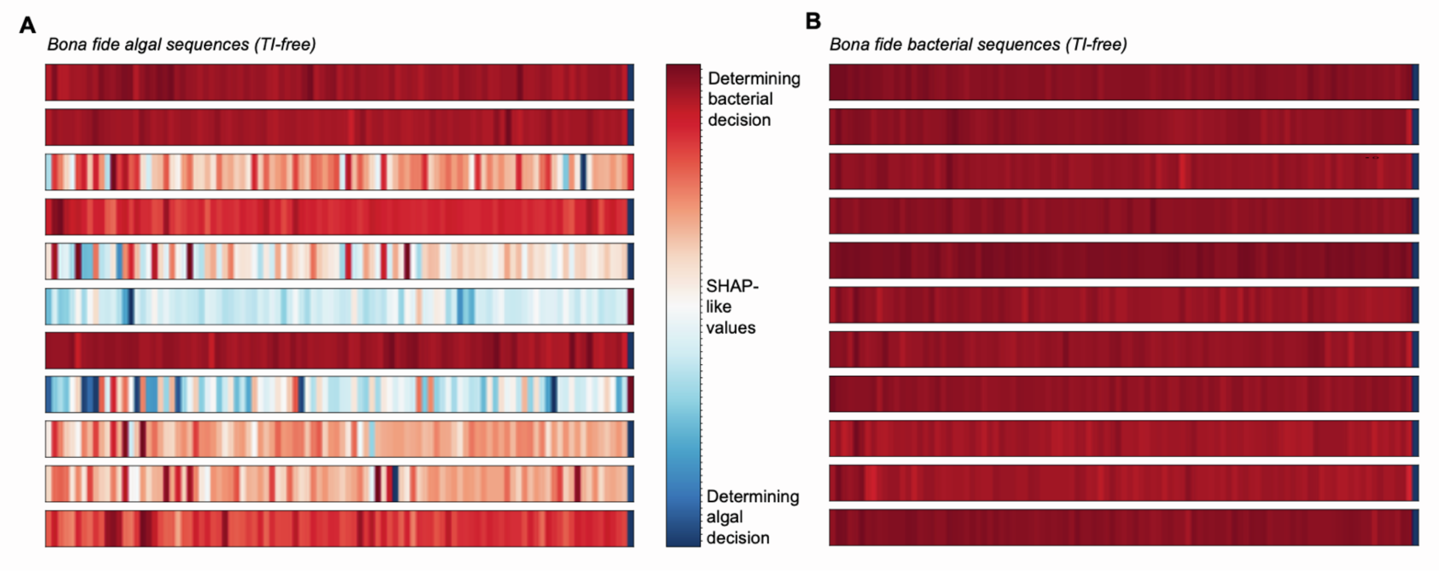

Figure S4. Feature attribution in bacterial and algal protein classification in a bi-directional BERT (distilRoBERTa)-based model.

(A) Heatmaps showing SHAP-like influence values for individual amino acids in TI-free algal protein sequences in a LA⁴SR -distilRoBERTa post-trained model. Each row represents a distinct protein sequence from the TI-free training set with amino acids displayed along the x-axis. Color intensity indicates the magnitude and direction of each amino acid's contribution to classification, with red signifying bacterial and blue algal prediction.

(B) Heatmaps showing SHAP-like influence values for individual amino acids in TI-free bacterial protein sequences in a LA⁴SR -distilRoBERTa post-trained model, similarly to (A).

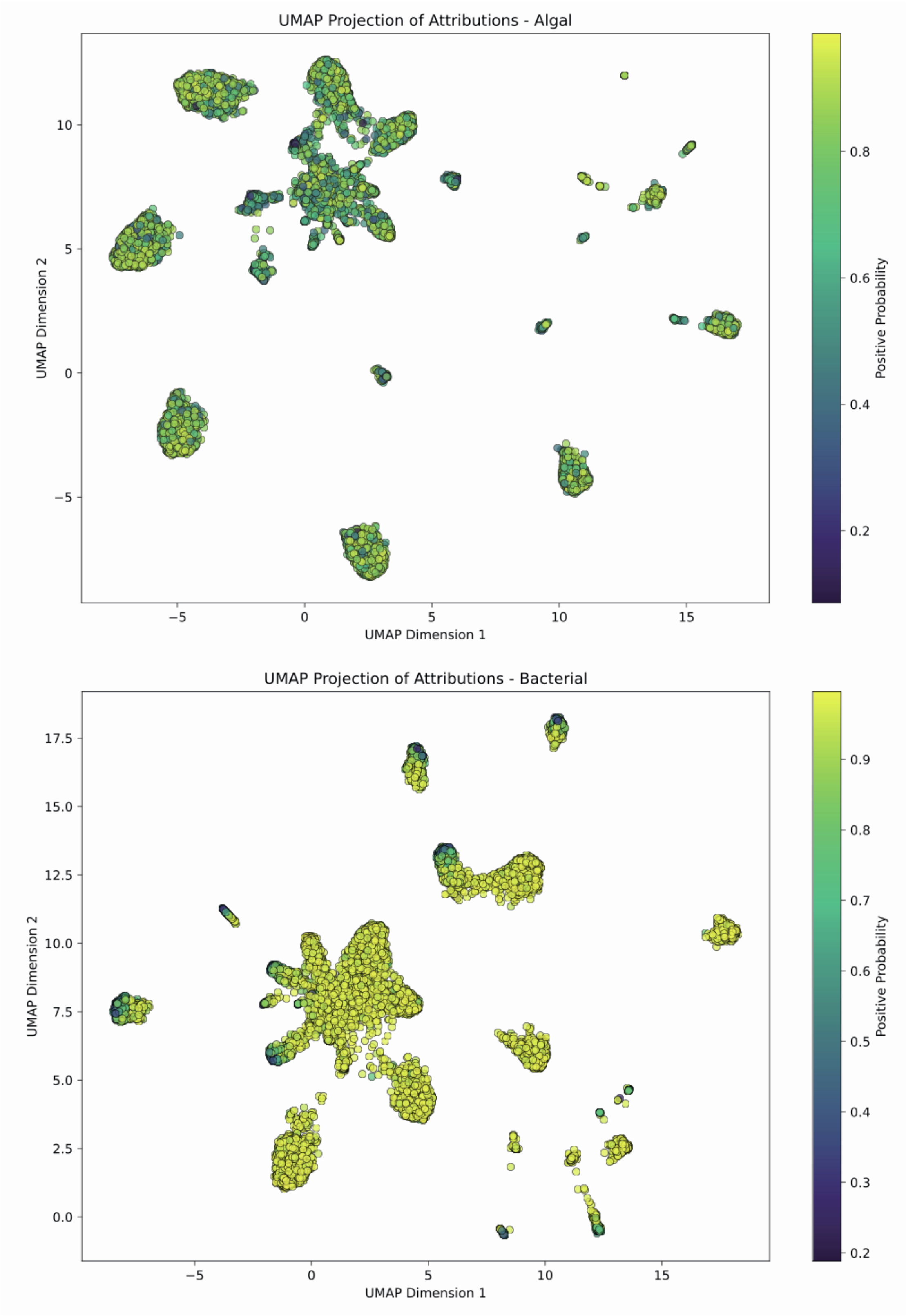

Figure S5. UMAP on attribution scores from 20,000 algal and bacterial sequences from LA⁴SR model transformer outputs

A comparison of bacterial and algal inputs was analyzed with the ALMGA TI-inclusive LA⁴SR model (Data S5). The tight clustering from UMAP reveals approximately ten large clusters of algal sequences, corresponding to the ten major algal phyla sampled in the training data, with a relatively low positive probability of being identified as a contaminant.

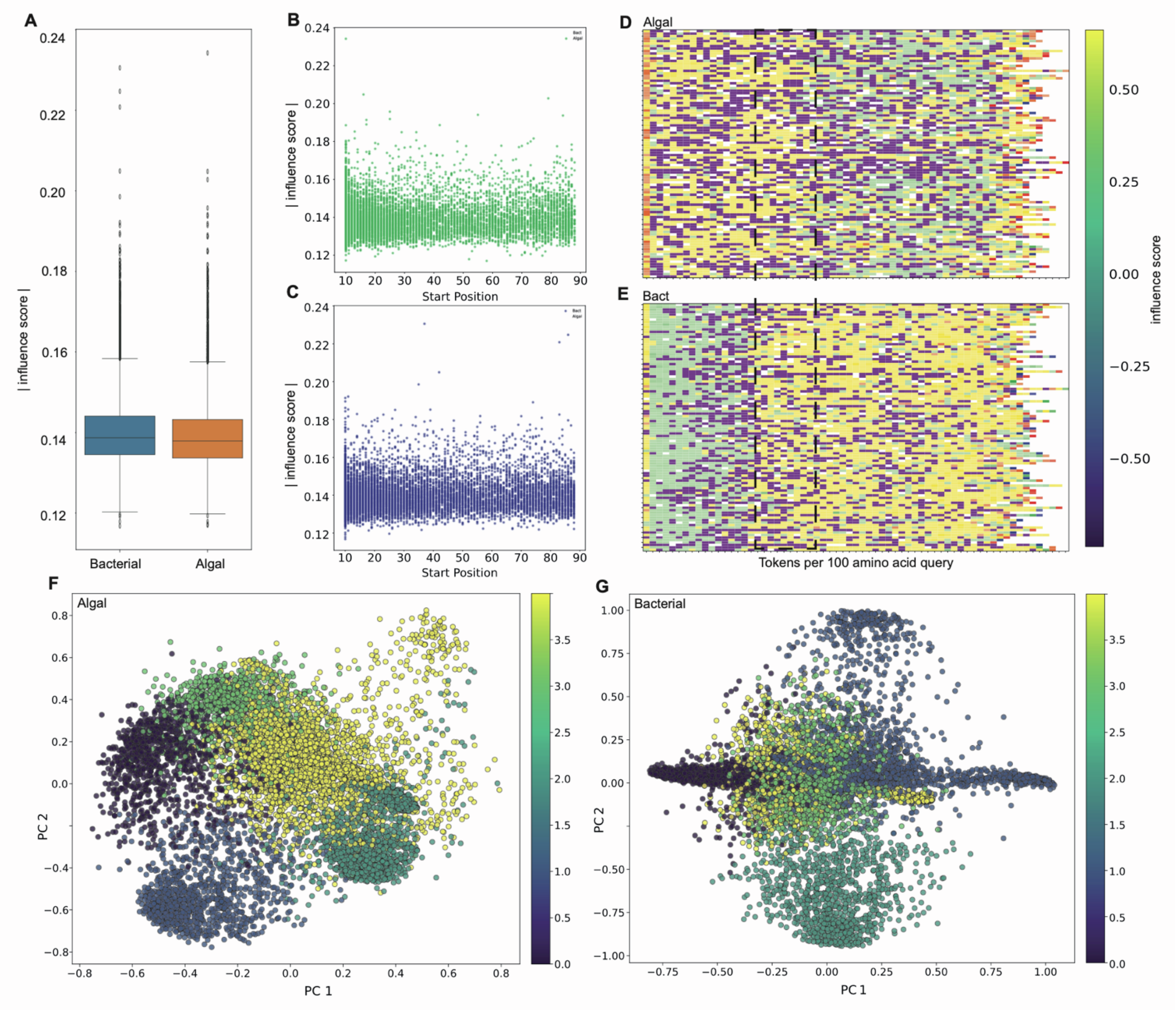

Figure S6. Captum-based analysis of algal and bacterial per-residue attribution scores

(A) Distributions of absolute influence scores in algal and bacterial sequences in a Pythia 70m-based LA^{4}SR model.

(B-C) Distributions of absolute influence scores per position in algal and bacterial sequences, showing a relatively even distribution of influence scores nearly independent of position.

(D-E) Heatmaps showing influence scores from sequences of tokens per 100 amino acid queries for algal (top) and bacterial (bottom) samples. The x-axis represents the position in the sequence, and the y-axis represents individual samples. The color scale ranges from -0.50 (blue: negative influence scores) to 0.50 (yellow: positive influence scores). The contrasting heatmaps give insight into the LA^{4}SR prediction process. The sequences around the 30 amino acid mark appear highly influential in the Pythia-based LA^{4}SR model. This finding was further supported by our motif-based analyses (Fig. 4), where the most influential three-to-six amino residue motifs were usually found 29-34 residues into the sampled sequence.

(F-G) PCA plots from layer-aggregated transformer outputs, corresponding to the main text Fig. 7. Color scales represent attributions.

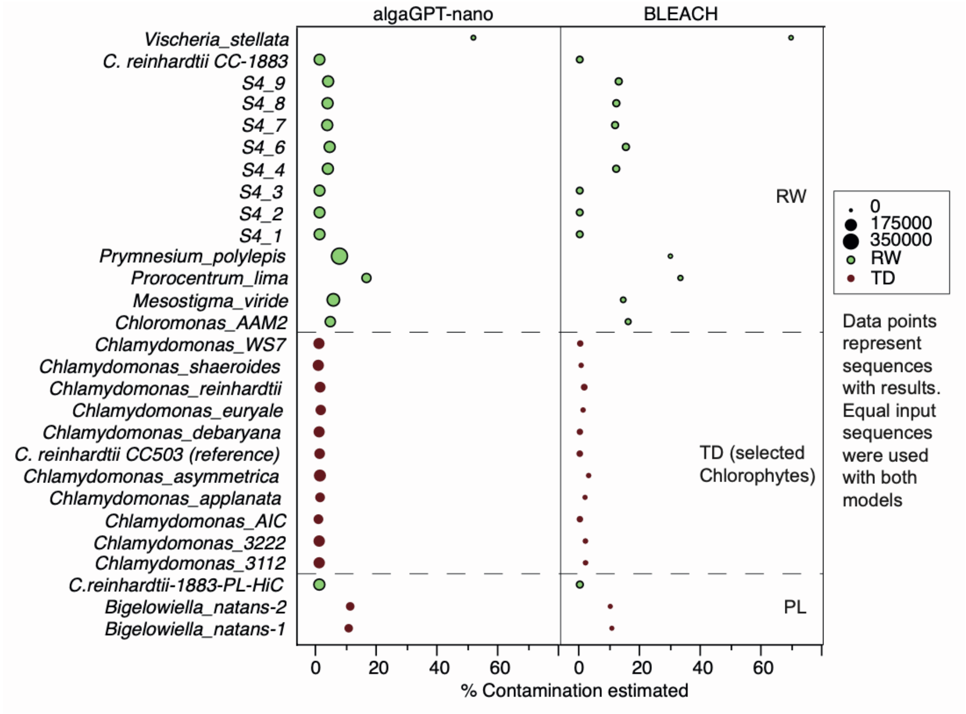

Figure S7. LA^{4}SR models perform well on new, real-world data

Shown are algaGPT-nano and BLEACH (BLAST-based contamination removal method)[S2] estimates of contamination in selected real-world (RW) sequencing, training datasets (TD), and platinum-level (PL) assemblies (i.e., chromosome-level, high coverage of long reads). Sequences known to be contaminated (e.g., the *Vischeria* and *Prorocentrum* assemblies) were compared with clean assemblies (e.g., assemblies from various species of *Chlamydomonas*[S3] and *Bigelowiella)*[S3,S4] from the TD using BLEACH and algaGPT-nano. The Dovetail Hi-C *C. reinhardtii* 1883 and *Bigelowiella natans*[S4] assemblies are shown as platinum-level, *bona fide* axenic reference benchmarks.

SUPPLEMENTARY TABLES

Table S1 | External spreadsheet. This spreadsheet contains LA^{4}SR performance metrics, technical performance estimations, and preliminary BLAST screening results of genomes comprising the algal training data.

Table S2 | External spreadsheet. Captum attributions for 100 sequences each of algal and bacterial origin obtained using the LayerIntegratedGradients function.

Table S3 | External spreadsheet. Influential motifs found with the DeepMotifMinerPro (DMMP) software introduced in this work as well as their functional associations (e.g., PFAM and REACTOME annotations (see also Data S3)).

Table S4 | External spreadsheet. LA^{4}SR and Diamond BLAST[S1] results for data from new assemblies from seen species (Fig. S7), contaminated assemblies from unseen genera (Fig. S7), and clean assemblies from unseen genera (Fig. 8). For the LA^{4}SR results for genome assemblies from unseen genera, the genomes were published after the model was trained, and the genera shown were not included in the training dataset. Newly sequenced genomes were uploaded to NCBI SRA accession SUB14799921.

SUPPLEMENTARY DATASETS

Data S1 | Neural network training sequences (4GB). Dataset comprising DNA sequences processed in two formats: with terminal information (TI-inclusive) and terminal information-free (TI-free).

Data S2 | Training and inference scripts. The LA^{4}SR framework integrated several open-source software packages and models. We used LORA[S5] (Low-Rank Adaptation) and QLORA[S6] (Quantized Low-Rank Adaptation) for parameter-efficient post-training and used Mamba[S7,S8] as an alternative to transformer-based architectures. The Hugging Face Transformers library facilitated implementation, pretraining, and post-training of the open-source models. Training was performed on an HPC cluster, with jobs going to nodes with NVIDIA (Santa Clara, CA, USA) V100, A100, or H100 GPUs.

Data S3 | Interpretability software developed in this study. Includes scripts for the implementation of the custom explainer programs presented with this work, including Captum[S9]-, DeepLift[S10], and SHAP[S11]-based approaches (Data S3) to explain how different amino acid residues and their patterns and positions affect model decisions.

Data S4 | Additional validations. To validate our approach on new clean assemblies from seen taxa, contaminated assemblies from unseen taxa, and new clean assemblies from unseen taxa, we performed additional validations. We applied LA^{4}SR models to data from new assemblies from seen species (Fig. S7), contaminated assemblies from unseen genera (Fig. S7), and clean assemblies from unseen genera (Fig. 8). We downloaded 12 new genomes from NCBI that had been published after the model was trained or otherwise not included in the training data; these genomes constitute the clean, unseen genera (Fig. 8). These unseen genera included two macroalgal genomes (Asparagopsis taxiformis and *Agarophyton chilense*) and one alga lacking chlorophyll (*Prototheca cutis*).

We cultured and sequenced ten separate isogenic colonies of *Chlamydomonas reinhardtii* CC-1883. Of these, nine were sequenced with Illumina 150 bp paired-end short reads and one with Pacific Biosciences (PacBio, Menlo Park, CA, USA) HiFi reads and DoveTail (Sydney, Australia) Hi-C to generate a complete, axenic reference assembly (Fig. S3; Data S4).

Data S5 | Singularity container to run LA^{4}SR. Includes an environment supporting LA^{4}SR, with over 200 dependency packages installed and organized, and run scripts, base programs, and example data inputs and outputs.

SI BIBLIOGRAPHY

S1. Buchfink, B., Xie, C., and Huson, D.H. (2015). Fast and sensitive protein alignment using diamond. Nat Methods. *12*, 59-60. https://doi.org/10.1038/nmeth.3176.

S2. Nelson, D.R., Mystikou, A., Jaiswal, A., Rad-Menendez, C., Preston, M.J., De Boever, F., El Assal, D.C., Daakour, S., Lomas, M.W., Twizere, J.C., et al. (2024). Macroalgal deep genomics illuminate multiple paths to aquatic, photosynthetic multicellularity. Mol Plant. *17*, 747-771. https://doi.org/10.1016/j.molp.2024.03.011.

S3. Nelson, D.R., Hazzouri, K.M., Lauersen, K.J., Jaiswal, A., Chaiboonchoe, A., Mystikou, A., Fu, W., Daakour, S., Dohai, B., Alzahmi, A., et al. (2021). Large-scale genome sequencing reveals the driving forces of viruses in microalgal evolution. Cell Host Microbe. *29*, 250-266 e258. https://doi.org/10.1016/j.chom.2020.12.005.

S4. Curtis, B.A., Tanifuji, G., Burki, F., Gruber, A., Irimia, M., Maruyama, S., Arias, M.C., Ball, S.G., Gile, G.H., Hirakawa, Y., et al. (2012). Algal genomes reveal evolutionary mosaicism and the fate of nucleomorphs. Nature. *492*, 59-65. https://doi.org/10.1038/nature11681.

S5. Hu, E.J., Shen, Y., Wallis, P., Allen-Zhu, Z., Li, Y., Wang, S., Wang, L., and Chen, W. (2021). Lora: Low-rank adaptation of large language models. Preprint at arXiv, https://doi.org/10.48550/arXiv.2106.09685.

S6. Dettmers, T., Pagnoni, A., Holtzman, A., and Zettlemoyer, L. (2023). Qlora: Efficient finetuning of quantized llms. Preprint at arXiv, https://doi.org/10.48550/arXiv.2305.14314.

S7. Dao, T., and Gu, A. (2024). Transformers are ssms: Generalized models and efficient algorithms through structured state space duality. Preprint at arXiv, https://doi.org/10.48550/arXiv.2405.21060.

S8. Gu, A., and Dao, T. (2023). Mamba: Linear-time sequence modeling with selective state spaces. Preprint at arXiv, https://doi.org/10.48550/arXiv.2312.00752.

S9. Kokhlikyan, N., Miglani, V., Martin, M., Wang, E., Alsallakh, B., Reynolds, J., Melnikov, A., Kliushkina, N., Araya, C., Yan, S., and Reblitz-Richardson, O. (2020). Captum: A unified and generic model interpretability library for pytorch. Preprint at arXiv, https://doi.org/10.48550/arXiv.2009.07896.

S10. Shrikumar, A., Greenside, P., and Kundaje, A. (2017). Learning important features through propagating activation differences. Preprint at arXiv, https://doi.org/10.48550/arXiv.1704.02685.

S11. Contreras, J., Winterfeld, A., Popp, J., and Bocklitz, T. (2024). Spectral zones-based shap/lime: Enhancing interpretability in spectral deep learning models through grouped feature analysis. Anal Chem. *96*, 15588-15597. https://doi.org/10.1021/acs.analchem.4c02329.
