# Supplementary material for: Pan-microalgal dark proteome mapping via interpretable deep learning and synthetic chimeras
Source: Patterns (N Y). 2025 Sep 24;6(11):101373. doi: 10.1016/j.patter.2025.101373 (PMC12664985; doi:10.1016/j.patter.2025.101373)
Supplement: Document S2. Article plus supplemental information [file mmc6.pdf]

Article

Patterns

Pan-microalgal dark proteome mapping via interpretable deep learning and synthetic chimeras

Graphical abstract

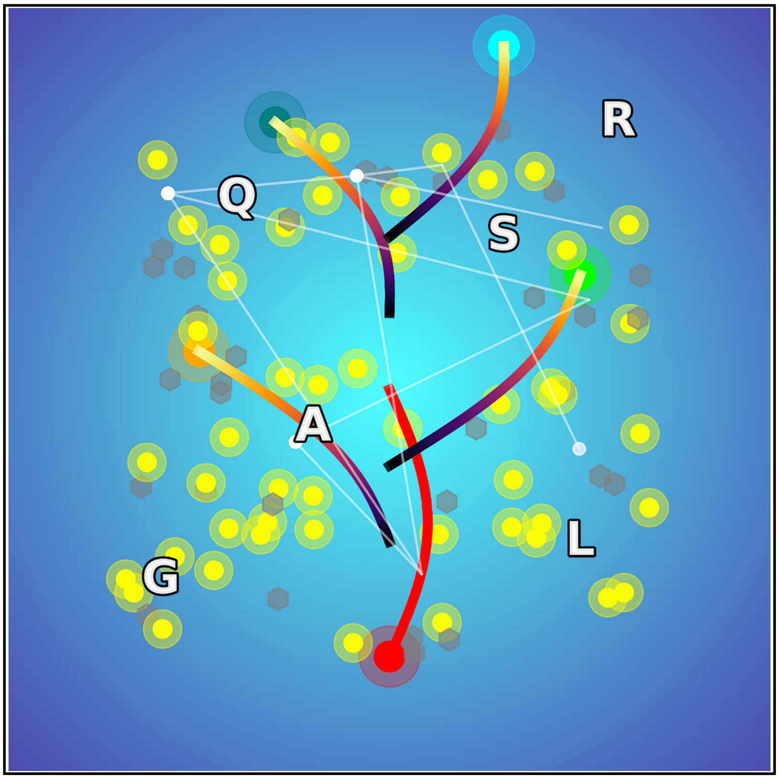

Highlights

- LA^{4}SR classifies microalgal and contaminant sequences with near-complete recall
- Training on internal sequence features alone facilitates robust classification
- Inference runs ~10,701× faster than BLASTP$^+$, enabling large-scale proteome annotation
- Model explainability links amino acid patterns to evolutionary and biophysical features

Authors

David R. Nelson,
Ashish Kumar Jaiswal,
Noha Samir Ismail,
Alexandra Mystikou,
Kourosh Salehi-Ashtiani

Correspondence

drn2@nyu.edu (D.R.N.),
ksa3@nyu.edu (K.S.-A.)

In brief

The vast microbial "dark proteome" remains largely uncharacterized. Nelson et al. present LA^{4}SR, a transformer framework that classifies algal sequences 10,000× faster than traditional methods with near-perfect recall. Trained on 77 million sequences including synthetic chimeras, the system can robustly classify unknown sequences with natural or scrambled start and stop information. They also developed an accompanying suite of explainability software to reveal biologically meaningful patterns linking amino acid patterns to evolutionary affiliations.

Check for updates

Nelson et al., 2025, Patterns *6*, 101373
November 14, 2025 © 2025 The Author(s). Published by Elsevier Inc.
https://doi.org/10.1016/j.patter.2025.101373

CellPress

Patterns

CellPress
OPEN ACCESS

Article

Pan-microalgal dark proteome mapping via interpretable deep learning and synthetic chimeras

David R. Nelson,[1,5,*] Ashish Kumar Jaiswal,[1] Noha Samir Ismail,[1,2] Alexandra Mystikou,[1,3,4] and Kourosh Salehi-Ashtiani[1,*]

[1]Laboratory of Algal, Artificial Intelligence, Synthetic, and Systems Biology (A2S2 Group), Division of Science and Math, New York University Abu Dhabi (NYUAD), Abu Dhabi, UAE

[2]Department of Biology, New York University, New York, NY, USA

[3]Biotechnology Research Center, Technology Innovation Institute (TII), PO Box 9639, Masdar City, Abu Dhabi, UAE

[4]Present address: Omics Center of Excellence, M42 Health, Abu Dhabi, UAE

[5]Lead contact

*Correspondence: drn2@nyu.edu (D.R.N.), ksa3@nyu.edu (K.S.-A.)

https://doi.org/10.1016/j.patter.2025.101373

THE BIGGER PICTURE Microbes drive Earth's energy flow in all of Earth's ecosystems, yet most microbial proteins remain uncharacterized. Large-scale sequencing projects have gathered stores of environmental DNA from diverse geographies, but many of these are without matches in current sequence databases, forming what is known as the "dark proteome." Traditional computational methods for classifying these mysterious proteins are prohibitively slow, failing entirely when proteins are too evolutionarily distant from known sequences. This creates a critical bottleneck because we are generating data far faster than we can interpret them. Recent advances in AI, particularly language models originally developed for human text, offer a promising path forward by learning patterns directly from protein sequences. These approaches could transform how we explore microbial diversity, potentially unlocking new high-value compounds, enzymes for biotechnology, and insights into how life adapts to extreme environments.

SUMMARY

Microalgal genomes contain a vast "dark proteome"—sequences lacking detectable homology that evade conventional classification tools. We developed LA^{4}SR (language modeling with AI for algal amino acid sequence representation), a framework using transformer- and state-space models to classify translated ORFeomes across ten algal phyla. Training on ~77 million sequences, LA^{4}SR achieves near-complete recall, accelerates classification by ~10,701× relative to BLASTP$^+$, and generalizes robustly to unseen sequences using less than 2% of available data. Models trained on synthetic, chimeric (terminal information [TI]-free) sequences maintained high accuracy, demonstrating that internal sequence features alone can drive robust classification. Inference speed and scalability were further enhanced under TI-free settings, supporting rapid annotation of large proteomic datasets. Custom explainability tools revealed interpretable amino acid patterns linked to evolutionary and biophysical features. Designed for accessibility across disciplines, LA^{4}SR integrates biological context and computational innovation in parallel, enabling both biologists and data scientists to interrogate the microbial dark proteome.

INTRODUCTION

Protein sequence analysis seeks to uncover the biophysical, structural, and evolutionary principles that govern protein function across life, from individual residues to entire proteomes.[1,2] While N- and C-terminal regions often encode signals for localization, post-translational modification, and protein-protein interactions (i.e., terminal information [TI]), these features are typically more phylogenetically volatile than the internal domains responsible for core cellular functions. Transformer-based language models have demonstrated that rich, semantically meaningful representations of protein sequences can be learned directly from sequence data, obviating the need for traditional alignment-based search.

© 2025 The Author(s). Published by Elsevier Inc.
This is an open access article under the CC BY license (http://creativecommons.org/licenses/by/4.0/).

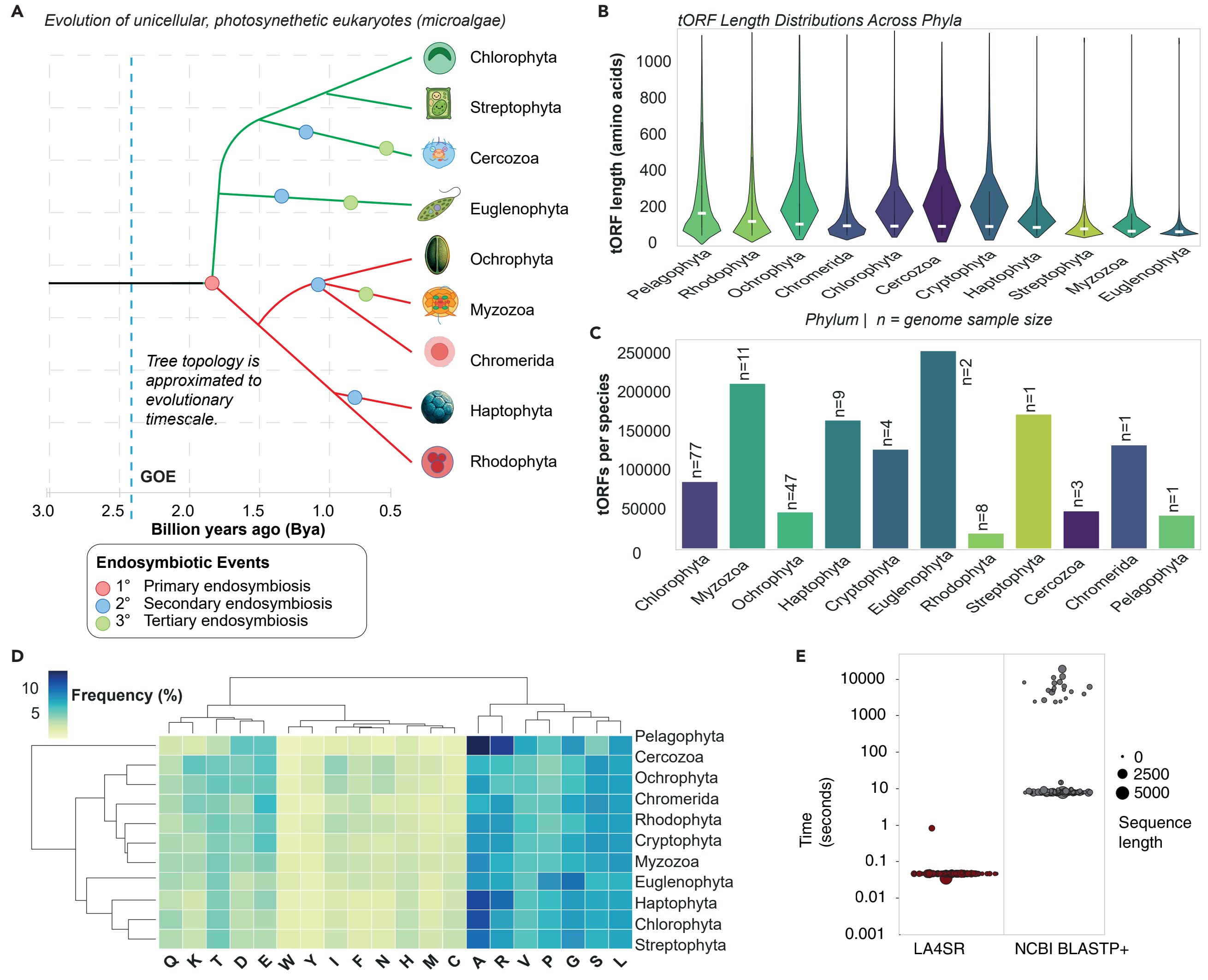

Figure 1. Evolutionary context, tORFeome characteristics, and performance benchmarks for microalgal sequence classification

(A) Approximated evolutionary history of plastids in unicellular photosynthetic eukaryotes (microalgae), as inferred from primary (red), secondary (blue), and tertiary (green) endosymbiosis events and molecular clock experiments.[7–9,10] Primary endosymbiosis of a cyanobacterium gave rise to plastids in the common ancestor of green and red microalgae. Secondary endosymbiosis of red algal plastids led to plastid-bearing lineages in the SAR clade (Ochrophyta, Myzozoa, and Chromerida) and Haptophyta, while green plastids were acquired independently by Euglenophyta and Cercozoa through secondary or tertiary endosymbiosis. Branching reflects plastid phylogeny—not host nuclear relationships—and highlights the reticulate nature of plastid transfer across diverse eukaryotic lineages. Tree topology approximates plastid evolutionary timelines based on molecular clock estimates. Plastid types are indicated by branch color; endosymbiosis events are marked by colored circles. The dashed vertical line marks the Great Oxidation Event.[11,12]

(B) Violin plots of protein (translated ORF [tORF]) length distributions (amino acids) for each phylum. White dots denote median lengths; numeric labels give the median value in amino acids.

(C) Bar chart of total proteins per species for each phylum (ordered left to right by median length from B); genome sample size (*n*) is annotated above each bar.

(D) Heatmap of amino acid frequencies (%) across phyla (rows) and residues (columns), hierarchically clustered by Euclidean distance (average linkage). The Ochrophyta-centric cluster (Pelagophyta, Cercozoa, Ochrophyta, and Chromerida) is defined by high serine (S) and leucine (L) levels, whereas Haptophyta unexpectedly groups with the Streptophyta-Chlorophyta clade driven by its elevated alanine (A) and arginine (R) content.

(E) BLASTP$^+$ and LA^{4}SR runtime (seconds) vs. sequence length (amino acids) for 257 randomly selected proteins. These measurements reflect runtime on a single processor, either a CPU (AMD EPYC 7742 @2.25 GHz; BLAST) or a GPU (NVIDIA A100; LA^{4}SR). Point size encodes sequence length category; point color indicates the fold-speedup by LA^{4}SR over BLASTP$^+$ (color bar at right). The gray band is a moving-box average of BLASTP$^+$ runtime. Extended runtimes are typical of sequences with sparsely represented relatives in the non-redundant (nr) database. An average of 10,701× speedup was observed from LA^{4}SR runs.

Proteome composition varies markedly across life's domains. Prokaryotic proteomes, despite undergoing extensive horizontal gene transfer,[3] often retain conserved functional architectures within defined clades.[4] In contrast, microalgal proteomes reflect compounded complexity from domain shuffling, alternative splicing, secondary and tertiary endosymbioses, and ancient chimerism (Figures 1A–1D).[5–9] These processes diversify protein structures and confound alignment- and *k*-mer-based

CellPress
OPEN ACCESS

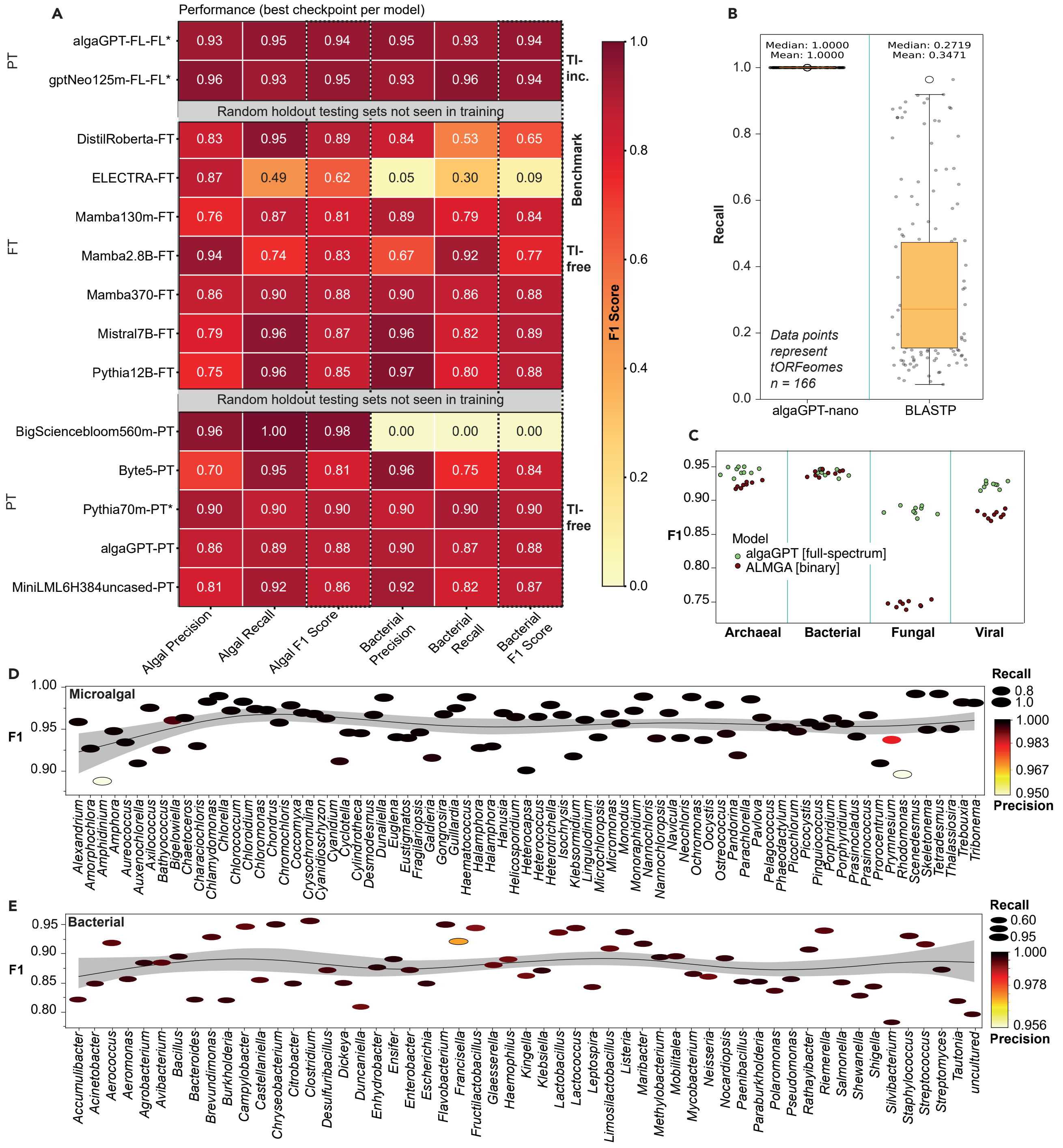

Figure 2. Performance comparison of language model architectures for amino acid sequence classification

(A) Pre-training (PT) from scratch with language model architectures produces high-accuracy, general-purpose amino acid sequence models. The best-performing checkpoint was used to create the final pre-trained model. Figure S1 shows further details of the experimental design. Fine-tuning (FT; i.e., post-training) existing pre-trained models also had high accuracy, often in fewer training steps. The heatmap shows the accuracy of pre- and post-trained models using various language model architectures for algal amino acid sequence classification: testing set = 1,000 full-length sequences (TI-inc.) or 10,000 synthetic chimeras (TI-free) per class as marked.

(B) Comparison of recall rates for ultra-sensitive Diamond BLASTP[11] and algaGPT on translated ORFeomes (tORFeomes) from n = 166 microalgal genomes.

(C) Sensitivity analysis showing performance of TI-inc. binary LA^{4}SR (ALMGA, Data S5)[13] on all-to-all pairings of three algae holdout sets with each contaminant group shown. The binary model was only trained on microalgae and bacteria, yet it generalizes to unseen groups. The algaGPT full-length model (Data S5)[13] was trained on the full spectrum of contaminants and performs notably better on the holdout fungal and viral testing sets. Total n = 1,000 sequences, each data point.

(legend continued on next page)

classifiers, which frequently misassign divergent or novel algal sequences. As a result, a large fraction of microalgal open reading frames (ORFs)—known as the "dark proteome"—remains unclassified (Figure 2B).

To overcome these limitations, we developed LA^{4}SR (language modeling with AI for algal amino acid sequence representation), a framework that leverages transformer- and S6-based language models to distinguish algal from contaminant sequences at scale. Trained on ~77 million sequences from 166 microalgal genomes spanning ten phyla (Data S1 [Data S1–S5 are available on Zenodo]),[13] LA^{4}SR achieves near-complete recall across the microalgal dark proteome and accelerates protein classification by 10,701-fold compared to traditional alignment methods. To support interdisciplinary use, LA^{4}SR integrates biological and computational insights throughout—from sequence encoding and model design to the interpretation of emergent amino acid patterns. The decision rule is

$$\hat{y} = \arg \max_{y \in \{\text{algal}, \text{bacterial}\}} p(y|x;\theta)$$

where $x = (a_1, .., a_L)$ is the query amino acid sequence of length L, $y \in \{\text{algal}, \text{bacterial}\}$ is a candidate class label, and $p(y|x;\theta)$ is the model-estimated posterior probability given parameters θ, and $\hat{y}$ is the resulting predicted class.

We designed "pan-microalgal" models to maximize generalizability across lineages, training on full-length, natural sequences (TI-inclusive, Figure S1) and synthetic chimeras with terminal regions and gene boundaries scrambled (TI-free, Figure S1). TI-free models, which relied solely on internal sequence features, matched TI-inclusive models in classification performance while achieving up to 10-fold inference speedups and exhibiting distinctly position-independent attribution profiles. These results demonstrate robust, scalable proteome classification even in the absence of complete or accurate gene annotations, offering a transformative approach for rapid microalgal genome analysis. Our open-source software suite (Data S2–S5)[13] provides researchers with immediately deployable models alongside flexible training frameworks for custom applications. The modular architecture enables straightforward adaptation to new taxonomic groups or sequence types. We provide documentation and worked examples to ensure reproducibility; all other training and evaluation programs are provided as well for accessibility across diverse research contexts.

RESULTS

LA^{4}SR enables classification of the microalgal dark proteome

A major barrier in microalgal genomics is the prevalence of "dark proteome" sequences—translated open reading frames (tORFs) that return no hits against public databases such as NCBI's non-redundant (nr) protein set. In our dataset of 166 microalgal genomes, approximately 65.3% of all tORFs were classified as "dark," escaping detection even under ultra-sensitive Diamond and basic local alignment search tool for proteins (BLASTP) and NCBI BLASTP$^+$.[14] Homology-based methods recovered only ~35% of total sequences, leaving the majority uncharacterized (Figure 2B and Table S1). The quantity of "dark matter" seen with alignment-based methods may be due to limitations in database representation and algorithm flexibility or generalizability.

In contrast, LA^{4}SR models demonstrated near-complete coverage of the microalgal proteomes, successfully classifying >99% of input sequences across all tested genomes, including many that were previously unclassifiable using homology-based tools. LA^{4}SR generalized effectively to novel, never previously seen sequences, including a variety of holdout sets and new real-world datasets comprising both axenic and xenic cultures (Figures 2, 8, and S7; Table S4; Data S4).[13] Models trained on synthetic chimeras generalized well to full-length sequences and performed better on scrambled protein sequence information (Figure 2A), indicating a superior performance on fragmented annotations.

We subjected all of the trained models to performance evaluations on holdout test sets as well as new data downloaded from NCBI and sequenced in-house. The top-performing models had nearly perfect recall not only on holdout sets but also across entire, unseen proteomes—including species with high genomic chimerism or extensive evolutionary divergence from the training data. This capacity to annotate previously inaccessible regions of the microalgal proteome marks a major advancement over alignment-based methods, bridging a critical gap in environmental genomics and facilitating downstream applications in assembly validation, contamination screening, and functional annotation. Together, these results establish LA^{4}SR as a transformative tool for illuminating the microalgal dark proteome, combining scalability and robustness to support emerging genomic efforts across diverse algal lineages.

LA^{4}SR outperforms alignment-based methods in speed and recall

Benchmarking against Diamond[11] and NCBI BLASTP$^+$ revealed dramatic performance advantages for LA^{4}SR. On a single NVIDIA A100 GPU, LA^{4}SR processed queries in 0.0378 ± 0.0029 s, significantly faster than 535.02 ± 2056 s for NCBI BLASTP$^+$[14] running on an AMD EPYC 7742 CPU (at 2.25 GHz), achieving an approximate 10,701-fold speedup (Figures 1E and 1F), where the speedup ratio was

$$\text{Speedup} = \frac{T_{\text{BLASTP}}}{T_{\text{LA}^4\text{SR}}} \approx 10,701 \times .$$

Against Diamond,[11] LA^{4}SR operated 82.9 times faster (Table S1). This acceleration remained consistent across diverse

(D) Sensitivity analysis showing performance of a TI-free LA^{4}SR model on natural, whole-genome *in silico* proteomes from various algal genera, most of which were represented in trained data as randomly split and fused chimeras. These metrics are summarized at the phylum level in Figure S3. Accuracy was slightly below 100 for most inference runs, indicating the absence of overfitting. F_1 score distributions aggregated by genus across ~80 microalgal genera.
(E) F_1 score distributions aggregated by genus across ~100 randomly sampled bacterial genera from NCBI (known contaminant taxa). The overlap with training data was estimated at 5%. Modest drops in the sparsest genera (e.g., *Tisochrysis* and *Campylobacter*) highlight priority targets for future data augmentation, while strong performance on novel bacterial proteomes demonstrates learning of core sequence features independent of translational initiation.

CellPress
OPEN ACCESS

sequence lengths and complexities (Figures 1E and 1F), enabling linear scaling to large-scale genomic and pan-genomic datasets. Notably, LA^{4}SR inference times were largely invariant to sequence size, in contrast to the non-linear runtime penalties typical of alignment-based approaches.

Beyond speed, LA^{4}SR closed critical annotation gaps: while classical methods failed to characterize ~65% of microalgal ORFs, LA^{4}SR classified this dark proteome with near-perfect recall (Figures 2A and 2B). Furthermore, the TI-free mode—scrambling terminal signals during training, generating synthetic, chimeric sequences—yielded an additional order-of-magnitude increase in token generation speed (Figure S2) while performing well in a microalgal lineage-stratified sensitivity analysis (Figure S3), further enhancing throughput without sacrificing accuracy. Together, these results demonstrate that LA^{4}SR is not only orders of magnitude faster than classical tools but also more sensitive, more scalable, and more robust to novel biological variation.

Dataset generation and model training strategies

To train and evaluate LA^{4}SR, we constructed large-scale microbial genomics datasets comprising ~77 million distinct protein sequences. These included translated ORFeomes (tORFeomes) from 166 microalgal genomes across ten phyla—spanning Chlorophyta, Rhodophyta, Haptophyta, Cercozoa, Ochrophyta, Myzozoa, Euglenophyta, Arachniophyta, Streptophyta, and Chromerida—combined 1:1 with bacterial, archaeal, and fungal contaminant sequences for full-length datasets and 1:1 algal-bacterial for TI-free scrambled datasets. The genomic datasets were derived from tORFeomes across diverse microalgal lineages, encompassing a wide range of natural protein lengths and amino acid compositions (Figure 1). Despite the heterogeneous distributions of available genomes throughout phyla (e.g., over-representation in Chlorophyta and Ochrophyta and under-representation in other clades), LA^{4}SR performed well across the pan-microalgal genome. For example, TI-free LA^{4}SR was trained on synthetic chimeras from 77 Chlorophyta species but only four Rhodophyta species, reflecting the availability of genome sequences available from each clade. However, the F_1 scores only slightly decreased, from 98 to 94, when evaluated on full-length Chlorophyta and Rhodophyta proteins, demonstrating robust generalization.

Internal regions of proteins are generally more evolutionarily conserved, while termini are often more variable or incomplete in genome annotations.[12,15] By training on TI-free datasets, LA^{4}SR models emphasized internal sequence signals (Figure S1), promoting greater robustness to incomplete or misannotated gene models (Figure 1). Notably, despite scrambling gene boundaries, LA^{4}SR models maintained strong predictive performance, indicating that taxonomic signatures are broadly distributed across entire sequences rather than being restricted to termini. This generalizable strategy addresses critical limitations in cases of sub-platinum-level gene annotation. Input variability can result from RNA-sequencing-based transcriptomics where the full space of spliceosomal variation is not fully explored or with *in silico* ORFeome annotations that can lack contiguity because of complex or long-ranging splicing patterns.

Zero-shot transfer learning and early domain knowledge

A striking feature of LA^{4}SR's training dynamics was the rapid emergence of biologically meaningful classification patterns. Even after only 50 post-training steps, several models, including the 370-million (370m)-parameter Mamba, began to correctly distinguish algal from bacterial protein sequences, despite never having been explicitly trained for this task beforehand. This early performance likely stems from zero-shot knowledge transfer acquired during large-scale pre-training on natural language corpora such as The Pile,[16] a diverse dataset containing scientific literature (including PubMed articles) and source-code repositories (e.g., GitHub). These pre-training sources contain extensive biological information, indirectly exposing models to the statistical patterns of molecular biology, protein structures, and evolutionary terminology. The post-training data included nuclear, chloroplast, and mitochondrial genomes, as well as coding sequences from all known extrachromosomal compartments: secondary-plastid nucleomorphs (in cryptophytes and chlorarachniophytes),[17] plastid minicircles (in dinoflagellates),[10,18] natural plasmid-like elements (in diatoms and certain green algae),[19,20] and viral or episomal inserts. Non-nuclear genomes have been shown to transfer genes to the host nuclear genome[6]; thus, we did not discriminate for the sake of broader generalization.

The ability of LA^{4}SR models to generalize algal and bacterial signatures with minimal fine-tuning suggests that transformer and state-space architectures can leverage latent biological priors learned from unrelated textual domains. This powerful cross-domain transfer highlights the unique potential of language models for biological sequence analysis, where embedded representations of biological knowledge can be rapidly adapted to specialized genomic tasks.

Scaling across architectures: Transformer vs. S6 models

To evaluate the flexibility of LA^{4}SR across different model architectures and to set up a competitive model arena for selecting the best-performing models, we pre- and post-trained both transformer-based models (e.g., GPT-NeoX, Mistral, and Pythia) and state-space models based on the S6 Mamba framework. After generating hundreds of models, we evaluated their performance on the holdout testing sets, using true and false positives (TPs and FPs) to calculate precision, recall, and F_1 scores, where

$$\text{Precision} = \frac{\text{TP}}{\text{TP}+\text{FP}}, \text{Recall} = \frac{\text{TP}}{\text{TP}+\text{FN}},$$
$$F_1 = 2\frac{\text{Precision} \times \text{Recall}}{\text{Precision}+\text{Recall}},$$

with FN being false negative. Models exceeding 300m parameters consistently had F_1 scores above 88 after training on less than 2% of the available dataset, underscoring the efficiency of large model architectures for sequence classification tasks. Pre-training smaller models (≤300m) from scratch required longer training durations—approximately 20,000 steps—to reach comparable performance benchmarks (Figure 2).

Among the S6 models, the 370m-parameter Mamba emerged as the best performer, balancing high accuracy with rapid inference speed. Although larger Mamba models (780m, 1.4 billion [1.4B], and 2.8B parameters) had slightly higher peak accuracy, the improvements were marginal relative to the substantial increases in computational cost and runtime. Inference times increased sharply with model size for S6 architectures,

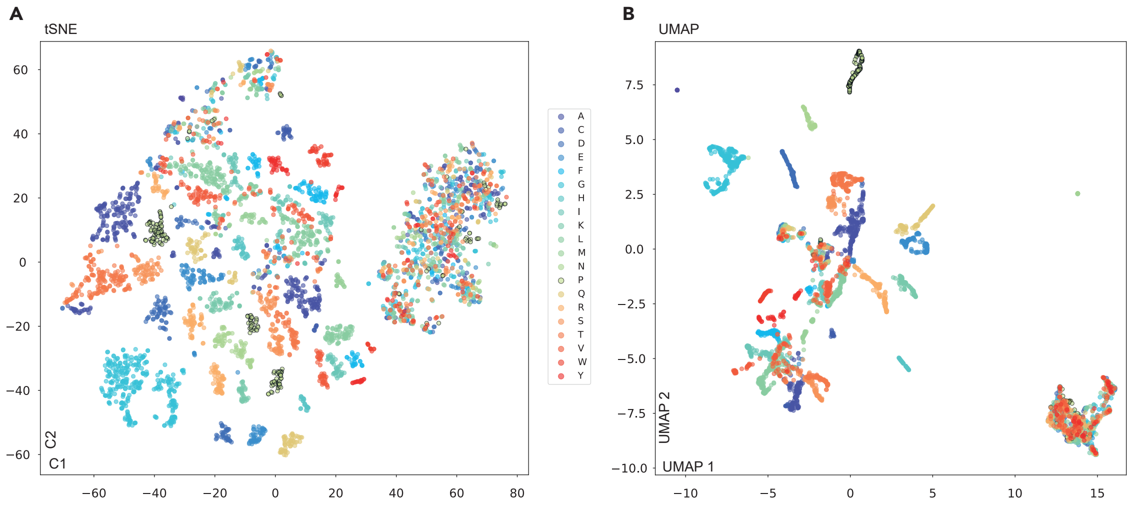

Figure 3. Dimensionality reduction reveals amino-acid-level importance patterns learned by the LA⁴SR model

(A) t-Stochastic neighbor embedding (t-SNE) visualization showing local clustering of individual amino acids based on their attribution scores. Amino acids largely group with others of the same type, suggesting that the model assigns similar levels of biological importance to identical residues across various bacterial sequences. A large, dispersed cluster containing mixed amino acid types reveals regions supporting functionally flexible or structurally variable regions.

(B) Uniform manifold approximation and projection (UMAP) visualization emphasizing global relationships between these amino acid attribution patterns. Like t-SNE, UMAP reveals amino acids primarily cluster by type, but also highlights broader relationships, indicating the model's recognition of conserved bacterial sequence characteristics across larger scales while simultaneously increasing resolution (see Figure S5 for algal sequence-based UMAP).

suggesting an optimal efficiency threshold around the 300–400m parameter range. Overall, LA^{4}SR demonstrated strong cross-architecture portability, achieving robust classification performance across both transformer and non-transformer models. This architectural flexibility highlights the generalizability of the framework and its potential for future adaptation to a broader range of genomic and proteomic challenges.

Model interpretability and feature analysis

What sequence features and molecular patterns drive LA^{4}SR models' decision-making process? To answer this fundamental question, we ran multi-faceted model interpretability[21] analyses, probing the neural networks' learning mechanisms. Adapting interpretability techniques from Tuned Lens,[22] Captum,[23] DeepLift,[24] and SHAP,[25] we systematically decoded how specific amino acid residues, their patterns, and positional relationships influence model decisions (Figures 3, 4, 5, 6, 7, and S4–S6). This analytical approach led to the development of model-agnostic gradient explainer tools (Data S3),[13] enabling us to extract and visualize crucial gradient information from multiple perspectives, such as per-residue, per-position, and per-motif attribution scores.

Figure 3 shows complementary dimensionality reduction analyses of amino acid attribution scores that reveal the structure of learned representations. t-Stochastic neighbor embedding (t-SNE) (Figure 3A) emphasizes local relationships, producing compact, circular clusters for each amino acid type, while uniform manifold approximation and projection (UMAP) (Figure 3B) preserves global structure through elongated, arc-like arrangements spanning larger embedding regions. This methodological contrast—discrete islands in t-SNE vs. continuous manifolds in UMAP—demonstrates how dimensionality reduction influences interpretation of model representations.

Notably, UMAP's curved trajectories suggest the model encodes continuous gradients between amino acid types rather than categorical distinctions. Both visualizations exhibit heterogeneous cluster densities, from tight homogeneous groups to dispersed regions, indicating context-dependent variation in amino acid importance. This structural heterogeneity reflects biological reality: the model captures both amino acids with specific functional roles (tight clusters) and positions with greater evolutionary flexibility (dispersed regions), demonstrating learned representations that mirror genuine sequence variability in proteins.

Amino acid usage patterns reflect GC bias and structural tendencies

We saw that glutamine and glycine dominated token usage frequency (Figures 4C and 4D). The disproportionate emphasis on glycine and glutamine in distinguishing between eukaryotic microalgae and bacteria highlights the importance of amino acid usage patterns in reflecting evolutionary adaptations to different ecological niches, particularly in relation to nitrogen metabolism. To investigate potential drivers of this pattern, we considered underlying genomic and structural features. First, codon usage and GC content offer a plausible explanation: glycine is encoded by four GC-rich codons (GGN), and its prevalence in bacterial sequences may reflect the broader variability in bacterial genomic GC content. Conversely, glutamine is encoded by AT-rich codons (CAA and CAG), and its enrichment in algal sequences may result from more constrained or conserved GC levels in algal nuclear genomes. Second, we hypothesize that LA^{4}SR models may implicitly capture structural signals related to intrinsic disorder. Glutamine is commonly enriched in disordered regions, which are more prevalent in eukaryotic proteins, particularly in regulatory and signaling domains.[30,31] Glycine, associated with

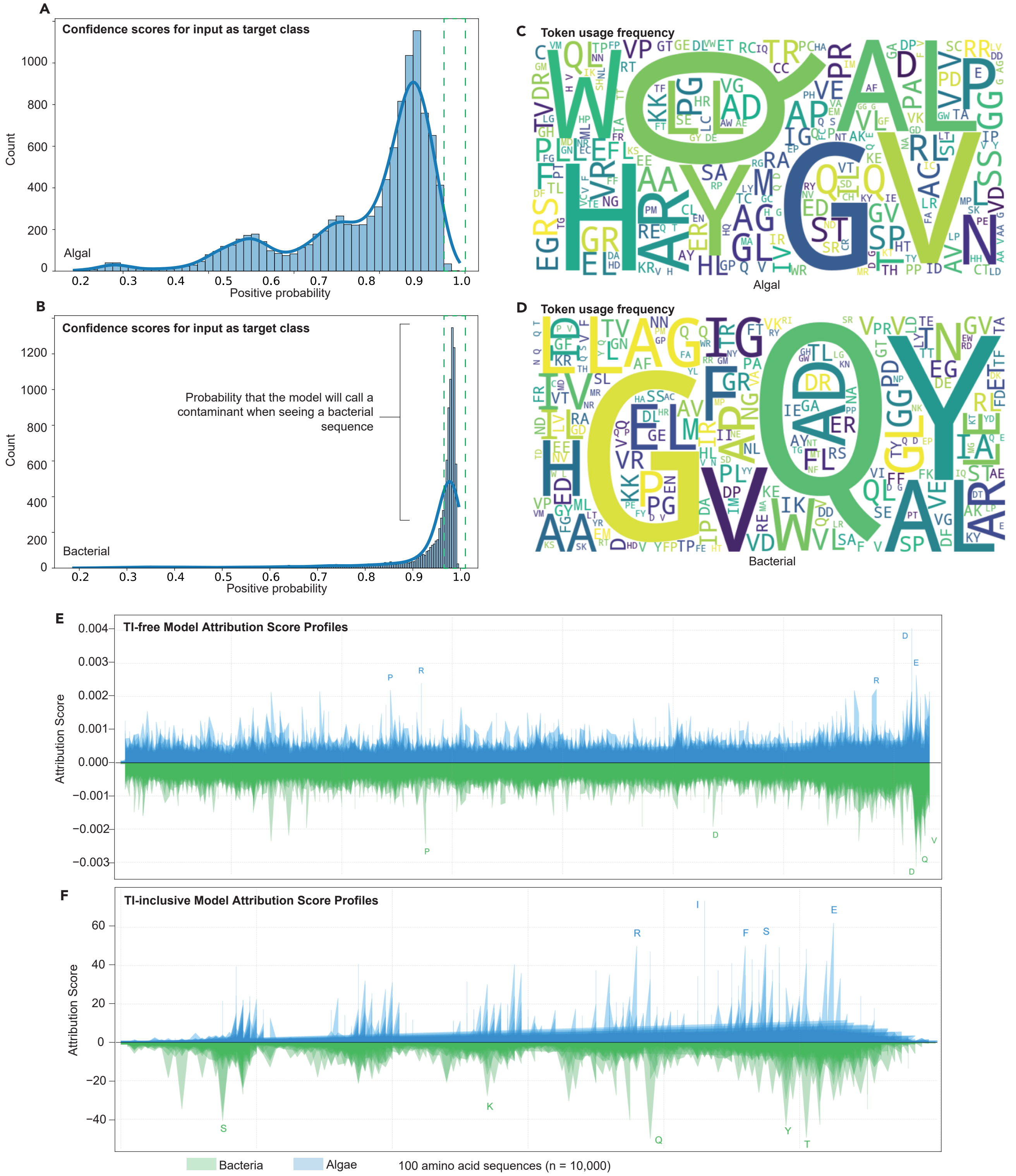

Figure 4. Model attribution analysis for sequence classification

(A) Confidence score distribution from Captum[23] attribution analysis evaluating the classification of algal sequences (see also Figure S6 and Table S2). Histograms show positive probability scores (0–1) across 20,000 TI-free representative algal and bacterial sequences, with higher values indicating stronger classification confidence. The algal distribution displays a multi-modal pattern, with a major peak at high confidence (>0.9) and secondary peaks at lower confidence, suggesting varying sequence-specific certainty.

(legend continued on next page)

structural flexibility, may be more frequent in bacterial proteins where compact, well-folded domains dominate.[32] These findings suggest that the model leverages both compositional and structural cues embedded in primary sequences to discriminate between microbial lineages, even in the absence of explicit evolutionary or functional annotations.

Interpreting decision making in LA^{4}SR through model dissection

Large language models (LLMs) have been notorious for their "black-box" opaqueness into their inner processes, but recent developments in interpretability tools facilitate explanation. We adapted several explainer packages to develop software allowing for inspection of protein language model (PLM) dynamics at varying scales. These programs, Hidden Embedding Layer Information Explorer (HELIX), DeepLift LA^{4}SR, and Deep Motif Miner Pro (DMMP), implement distinct approaches to transformer hidden state interpretation. For example, HELIX examines attributions at the per-layer scale, while DeepLift LA^{4}SR uses per-residue information. The DMMP software aggregates motif-based information and filters through thresholding to find influential motifs. Together, these interpretability packages provide insight into the model's evidence-accumulation dynamics in zero-shot proteomic classification.

DeepLift LA^{4}SR quantifies feature importance through attribution relative to a zero-reference state (Figures 4E and 4F). The method processes sequences in windows computing attribution scores $A = \sum(\mathrm{DL}(x, x_0))$, where A represents the attribution matrix, DL is the DeepLift operation comparing input sequence x against baseline reference x_0, w is the window size (32 residues), and stride is the step size (16 residues). Processing windows were necessary to avoid out-of-memory problems. The implementation wraps the embedding layer $E: \Sigma \rightarrow \mathbb{R}^d$ (where Σ denotes the amino acid alphabet and d is the embedding dimension) for direct gradient access, with attribution scores summed across embedding dimensions: $\mathrm{score}(i) = \sum_k A(i,k)$ for position i and embedding dimension k.

$$\Delta y = y(x) - y(x_0) = \sum_{i=1}^{L} \sum_{k=1}^{d} \frac{\partial y}{\partial x_{i,k}} (x_{i,k} - x_{0,i,k}),$$

where x_0 is the reference embedding and d is the embedding dimension. To examine where, along a 100-amino-acid input, the model draws its strongest class signal, we computed per-residue influence scores over 10,000 algal and bacterial queries using a sliding-window approach (window = 32 residues, stride = 16 residues). The resulting distribution of scores is broadly uniform across positions in the TI-free model, but spikes near the end of the windows and full sequence in the TI-inclusive model (Figures 4E and 4F).

A custom Captum[23] analysis pipeline (Data S3,[13] Table S2, and Figures S4–S6) was developed to analyze feature contributions using layer-f gradients analysis. For each input sequence, normalized attribution scores and model prediction probabilities were calculated, providing insights into which parts of the input most strongly influenced the model's decision-making process. The end-of-sequence token and the 2–5 preceding tokens, representing TI, had higher influence scores than sequences from other region trends (Figures 4E and 4F) in TI-free and TI-inclusive models, suggesting that this pattern is inherent to the transformer architectures we used and independent of the information in bona fide sequence ends. This trend was observed in all transformer models tested except for the bidirectional DistilRoBERTa[33–35] post-trained TI-free model, which demonstrated uniform influence scores (Figure S4). However, the accuracy of the DistilRoBERTa-based LA^{4}SR model was poor on the bacterial test set, showing its liability to potentially underestimate contaminants (Figure 2A).

To complement the Captum[23] approach and expand the capacity for explainer tools to non-causal language models, such as the DistilRoBERTa-based[33–35] LA^{4}SR model, we developed a Shapley additive explanations (SHAP)-like[25] gradient explainer to decipher sequence attribution (Data S3[13]). This software implements a custom gradient explainer to compute attribution values directly from PyTorch[36] output. The program quantifies the contribution of each amino acid to the model's classification decision and elaborates on features influencing the model (Figure S4). Our custom ShapExplainer class calculates these values by back-propagating from the loss of predicting specific tag tokens to the input embeddings, overcoming the limitations of traditional methods when applied to LLMs. For an input amino acid sequence $x = (a_1, \ldots, a_L)$ of length L, we construct the embedding matrix $E(x) = [e_1, .., e_L]^\top \in \mathbb{R}^{L \times d}$, where each vector $e_i \in \mathbb{R}^d$ is the learned d-dimensional representation of residue a_i. With a tag y, model output $F(x)$, and a loss of $\mathcal{L} = \ell(f(x), y)$, the gradient of the loss with respect to each embedding is

$$G = \frac{\partial \mathcal{L}}{\partial E(x)} \in \mathbb{R}^{L \times d}.$$

The SHAP-like contribution for each token i is then the dot product of gradient and embedding:

$$\phi_i = G_{i,\cdot} \cdot e_i = \sum_{j=1}^{d} \frac{\partial \mathcal{L}}{\partial e_{i,j}} e_{i,j},$$

(B) Corresponding probability distribution for bacterial test sequences (n = 10,000) showing a pronounced peak near 1.0, indicating a high-confidence classification of bacterial contaminants. The sharper, more concentrated distribution compared to algal sequences suggests more distinctive bacterial sequence features.
(C) Word cloud visualization representing token usage frequencies for algal inputs.
(D) Word cloud showing token usage frequencies for bacterial inputs.
(E) DeepLift[24] attribution profiles for TI-free model processing of the TI-free dataset, analyzed using a sliding window (window = 32 residues, stride = 16 residues) across 10,000 sequences per class. The generally flat shape reflects the model's distributed feature learning without terminal information. Shaded fits represent ~10% of the most conservative estimates. Letters above major peaks indicate key discriminative residues influencing classification decisions. A large terminal emphasis is observed, which is likely an artifact from the unidirectionality of the decoder-only architecture.
(F) DeepLift attribution profiles for the TI-inclusive model processing the same TI-free dataset. The TI-inclusive model exhibits stronger periodic patterns and higher-magnitude cumulative attributions across the measured sequence windows.

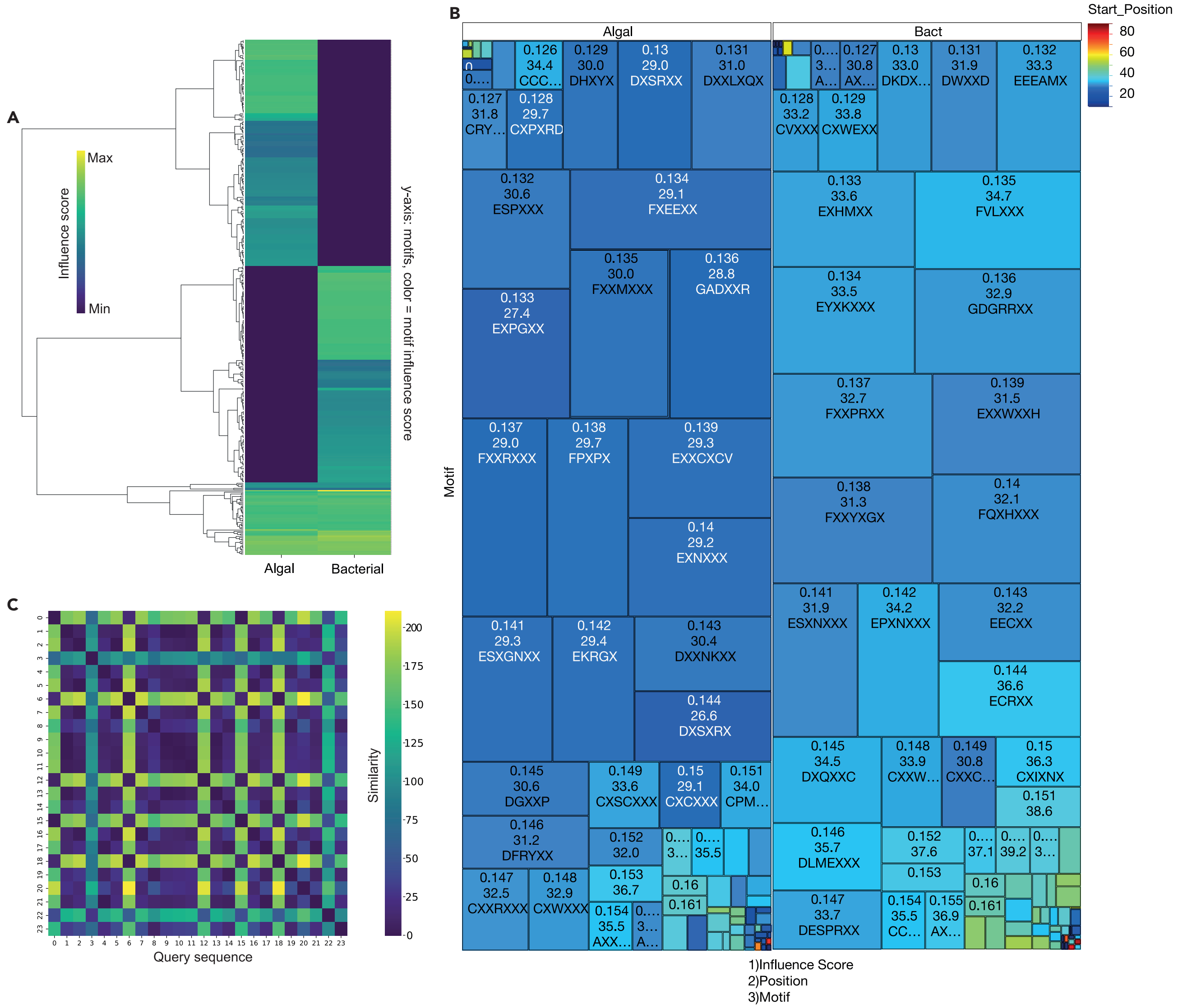

Figure 5. Examining sequence influence in LA^{4}SR with motif attribution for next-token prediction using Deep Motif Miner Pro

A GPT-NeoX[26] architecture-based pre-trained LA^{4}SR was used to process amino acid sequences and extract hidden state representations across multiple transformer layers. Flexible motif identification was then applied. The suite includes functionality to visualize amino acid representations in 2D space, analyze the influence of individual amino acids across model layers, and generate comprehensive heatmaps of flexible motif influences across multiple sequences. Altogether, 10,000 sequences for each of the algal and bacterial groups were analyzed; aggregative statistics are shown.

(A) A heatmap displaying the influence scores of various motifs in the query sequence. Motifs are categorized as either algal or bacterial, based on their ground-truth origin, not on their model determination.

(B) Influential motif compositions and positions, where X represents the position of variable residues. Algal motifs are, in general, slightly earlier in the average query sequence, which suggests an accelerated understanding of algal sequence patterns compared to bacterial sequence patterns. The text in white shows motifs starting earlier than 30 residues.

(C) Sequence similarity plot; sequences are the first 23 lines from the shuffled training set where the ground truth is known. The cross-hatch pattern shows similarity among motif distributions in the training algal and bacterial sequences used by the model to differentiate the two groups.

which collects across all positions to give the attribution vector $\phi = [\phi_1, \ldots, \phi_L]^\intercal = (G \odot E(x))\mathbf{1}_d$. Here, d is the embedding dimension, $\mathbf{1}_d$ $d \times 1$ is vector of ones, $\odot$ is the element-wise (Hadamard) product, and $f(x)$ is the model's forward output whose loss $\mathcal{L} = \ell(F(x), y)$ is back-propagated to get $G = \partial\mathcal{L}/\partial E(x)$.

Our SHAP-like value analysis of the DistilRoBERTa-based[33–35] LA^{4}SR model revealed class-specific patterns in its interpretation of sequences that were level throughout sequences and did not display spiking near termini (Figure S4), indicating that this bidirectional model eliminates bias seen in unidirectional models. Algal sequences displayed diverse attribution scores in SHAP-like attribution value heatmaps (visualized as varying shades of blue; Figure S4). In contrast, bacterial sequences showed more uniform patterns (predominantly colored in red). This consistency across bacterial samples indicates that the model has identified robust features characteristic of bacterial

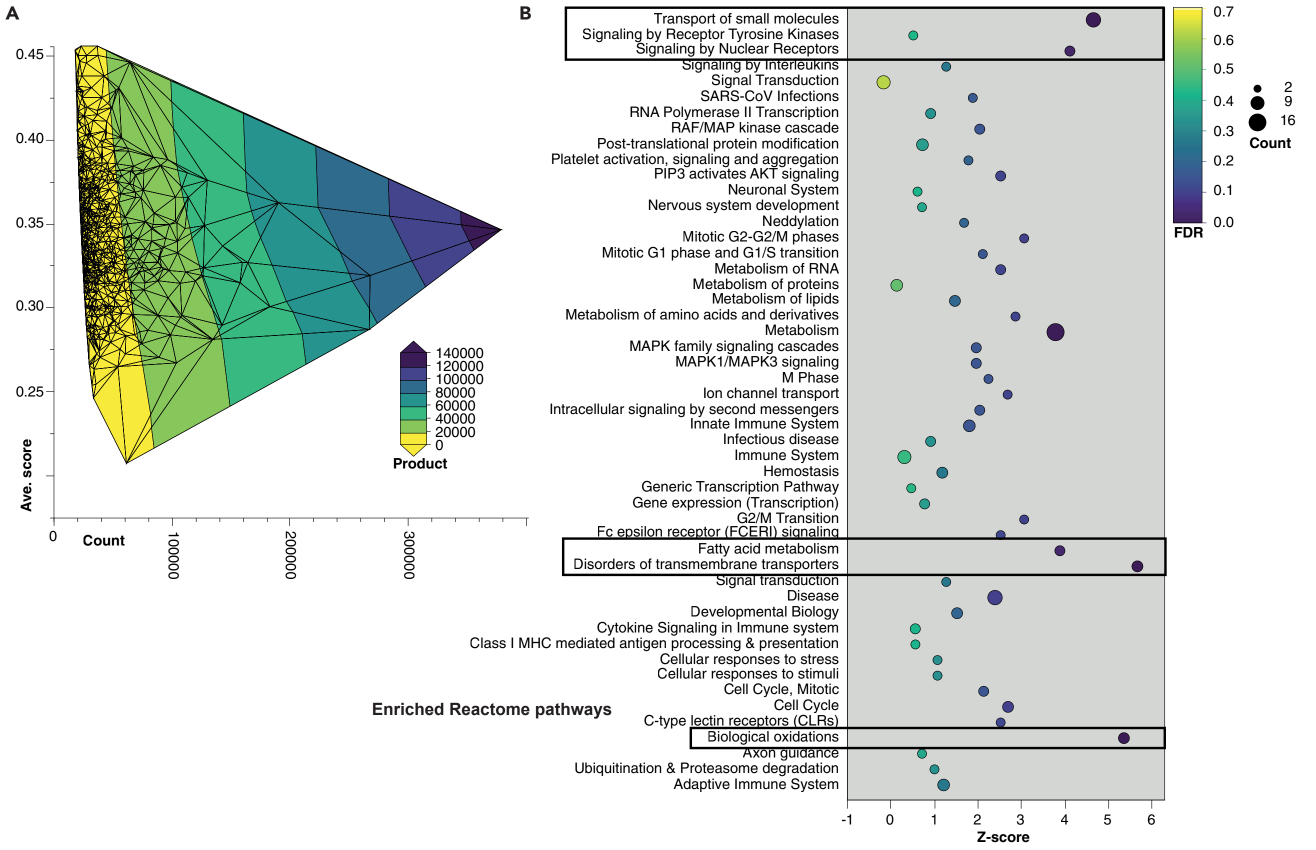

Figure 6. Integration of PFAM-A motif influence with Reactome enrichment

(A) Contour plot of PFAM-A motif distributions, with motif count on the *x* axis, mean per-residue attribution score on the *y* axis, contour lines denoting density, and color fill representing the product of count × score. The product of average influence score per motif and PFAM frequency was used to approximate relevance to known biological processes. The top 10,000 scoring PFAMs were used as dcGO[27] Reactome[28] enrichment queries (Table S3).

(B) Bubble plot of enriched Reactome[29] pathways showing *Z* score of enrichment (*x* axis) vs. Reactome pathway name (*y* axis), bubble area proportional to motif count and color indicating false discovery rate (FDR). Enrichment does not indicate the presence of the listed pathway in microalgae (e.g., microalgae do not get infected with SARS-CoV viruses, but they are enriched for this pathway because of shared PFAMs). The bounded boxes highlight three clusters: (1) transport of small molecules and receptor kinase/nuclear receptor signaling, (2) fatty acid metabolism and transmembrane transporters, and (3) biological oxidations. These enrichments reflect lineage-specific shared PFAM domains from biochemical programs implicitly learned by LA^{4}SR.

proteins. Conversely, diversity in algal attributions suggests a more complex, nuanced understanding of algal sequence features.

Influential motif identification and functional annotation

To identify sequence motifs that drive model predictions, we developed two complementary attribution-based methods: a flexible motif finder for visualizing degenerate influence patterns across model layers and a strict motif extractor optimized for downstream functional annotation. Influential motifs were detected using DMMP, a tool we developed that computes position-specific influence scores from hidden states by quantifying the deviation of each token's embedding from the mean representation (Table S3). For every residue identity *a* (one of the 20 canonical amino acid letters or "X") and every aligned position *i* along the sequence ($1 \le i \le L$), we measure how far the model's embedding for *a* departs from the background at the same position. Thus, the class separation index is $I(a, i) = \left\| \mu_{a,i} - \mu_{\neg a,i} \right\|_2$, where $\mu_{a,i}$ is the mean embedding vector of all tokens that carry residue *a* at position *i*; $\mu_{\neg a,i}$ is the mean embedding of the complementary set—tokens that are *not a* at that position; and $\| \cdot \|_2$ denotes the Euclidean norm, capturing the magnitude of the vector difference. The threshold for "high-importance" sites was $\theta = P_{95}(\{I(a, i)\})$, which is the 95th percentile across the entire collection of $I(a,i)$ values.

In DMMP, the motif seed set was calculated as $M = \{(s, p) | I(s[p], p) > \theta\}$, where *s* indexes a protein in the corpus and *p* denotes a position in that protein. Each ordered pair (s,p), therefore, points to a residue whose identity contributes unusually strongly, relative to alternatives at the same alignment column. Contiguous stretches that contain at least two such high-importance residues are extended symmetrically until the signal falls back to baseline, yielding variable-length motifs of 5–9 residues. Degenerate notation (e.g., "XRXDX") summarizes these motifs and highlights the positional constraints the language model has learned to associate with algal vs. bacterial identity (Figure 5 and Table S4).

Input sequences display sharp increases in both magnitude of influence and variability at residues 29–34 (Figure 5). From a machine-learning perspective, this arises because (1) our DMMP

CellPress
OPEN ACCESS

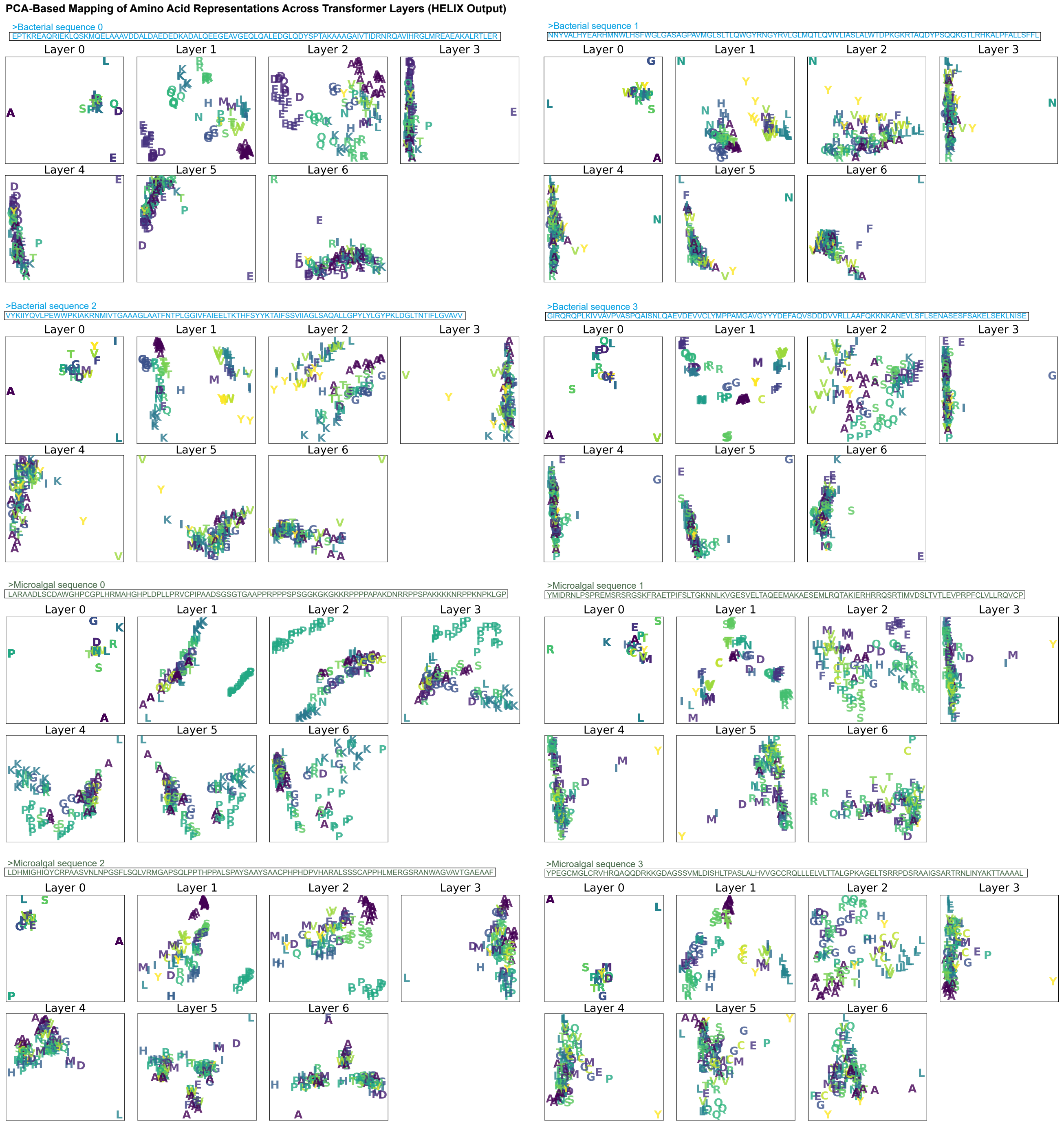

Figure 7. Layer-wise evolution of feature representations in a TI-free Pythia-based LA^{4}SR model

The progressions of amino acid feature representations through the layers of a deep-learning model trained on algal (green) and bacterial (blue) genomic data as detected in HELIX (Data S3)[13] are shown. The scatterplots depict the distribution of amino acid features from the synthetic chimera TI-free sequences in 2D space after dimensionality reduction with PCA for each layer of the neural network. Each point represents an amino acid. Each scatter subplot shows performance at an individual layer from analysis on a single algal sequence. This visualization demonstrates the neural network's transformation of genomic data into discriminative features based on their per-residue attribution scores. The increasing separation in deeper layers reflects the model's growing ability to distinguish between genomic signatures in both groups. The consistent presence of all classes across layers indicates maintained information throughout the network's depth. Expanding axis ranges in deeper layers suggest feature space expansion for improved separation. The figure visualizes feature representations across seven neural network layers (layer 0 to layer 6). Colors and shapes denote different residues as indicated in the legend. Layer 0 (input layer): a dispersed distribution with some clustering but significant class overlap. Layers 1–3: progressive feature separation, indicating learning of algal sequence type differentiation. Layers 4 and 5: further refinement of feature clusters and multi-dimensional separation. Layer 6 (output layer): the most pronounced separation suggesting refined features.

motif mining consistently locates the most discriminative 3–6 residue patterns in that 29- to 34-residue hotspot, and (2) the second attribution window (positions 16–47) is the first to fully encompass those motifs. By the time those signals are ingested, the unidirectional transformer's posterior probability has already saturated (often >90% confidence), so subsequent residues contribute only marginal adjustments. This "one-third" effect is therefore an emergent artifact of both where the model has learned its highest-impact motifs and how evidence is aggregated via overlapping windows.

To link high-confidence motifs to biological function, we applied a stricter extraction pipeline that combined attention, Captum's integrated gradients, and hidden state divergence into a composite score per position. Integrated gradients were calculated as

$$\mathrm{IG}_x(x) = \left(x_i - x_{0,i}\right) \int_{\alpha = 0}^{1} \frac{\partial F(x_0 + \alpha(x - x_0))}{\partial x_i} d\alpha,$$

where x_i and $x_{0,i}$ are the d-dimensional embedding vectors for residue i in the sequence and an all-padding baseline, respectively; F is the model's pre-soft-max logit for the algal class; and the line integral over $\alpha \in [0,1]$ accumulates gradients as the input is morphed from baseline to true sequence, producing the integrated-gradients attribution $\mathrm{IG}_i(x)$. All other symbols follow standard conventions: $x = (x_1, \ldots, x_L)$ is the full embedded sequence of length L, and d is the embedding dimension.

Thresholding was performed as in the flexible motif search (Figure 6). To evaluate the biological relevance of the learned motifs, we scanned the PFAM-A database for exact matches and assigned each match a double-weighted score—the product of motif frequency and mean influence—across 60,801,532 motif-domain pairs (Figure 6). Strikingly, the 10,000 highest-scoring pairs collapsed to just 65 unique PFAM accessions, and the top 100,000 pairs collapsed to only 335 PFAMs, with many mapping to archetypal eukaryotic functions that plausibly differentiate microalgae from contaminants.

Prominent examples include nucleic-acid and signaling modules—C2H2 zinc fingers (PF00096), RNA-recognition motifs (PF00076), Ras GTPases (PF00071), protein kinase catalytic cores (PF00069, PF07714), and rhodopsin-like 7-TM receptors (PF00001)—alongside scaffold repeats such as WD40 β propellers (PF00400), ankyrins (PF12796), and leucine-rich repeats (PF13855, PF13516). Membrane-transport and metabolic domains were also over-represented, including ABC transporters (PF00005), major facilitator superfamily permeases (PF07690), cytochrome P450s (PF00067), mitochondrial carrier proteins (PF00153), and AAA-ATPases (PF00004). Extracellular matrix and adhesion motifs—fibronectin type III (PF00041), cadherins (PF00028), EGF-like (PF00008) and calcium-binding EGF repeats (PF07645), CUB domains (PF00431), collagen triple-helix repeats (PF01391), and LDLR class A repeats (PF00057)—underscore the complex cell-surface architectures characteristic of many algal lineages. Catalytic and chaperone components such as trypsin proteases (PF00089), short-chain dehydrogenases (PF00106), DnaJ domains (PF00226), and P-type E1-E2 ATPases (PF00122) further highlight the biochemical breadth captured by the model (Figure 6).

The top-scoring double-weighted motif-PFAM assignments were further integrated with dcGO[27] (http://www.protdomainonto.pro/dcGO)-based enrichment analyses. Here, we show Reactome[29] database results as a well-curated resource for mapping PFAMs to known biological functions and processes (Table S3), Beyond Reactome, the results are amenable to use any ontology collection offered by the portal—including Gene Ontology (GO) terms, Kyoto Encyclopedia of Genes and Genomes (KEGG) pathways (KEGG, PANTHER, WikiPathway, and MitoPathway), Hallmarks (MSigDB), Regulators (ENRICHR and TRRUST), diseases (MONDO, DO, and EFO), drugs (DGIdb and tractability buckets), and phenotypes (HPO and MPO)—where the platform automatically applies one-tailed enrichment tests and multiple-testing correction, enabling comprehensive downstream exploration.

Enriched Reactome pathways included nucleotide metabolism, protein phosphorylation, and organellar translation, consistent with the biochemical roles of motifs identified in both bacterial and algal proteomes. Collectively, this multi-tiered approach revealed that interpretable deep-learning models can recover evolutionarily meaningful motifs and assign them to specific biological processes.

Layer-wise, per-residue, and per-position attributions: HELIX

To gain deeper insights into how LA^{4}SR models accumulate classification evidence along a sequence, we implemented both Captum's integrated gradients and a custom PyTorch[36] attribution pipeline (Data S3)[13] to probe hidden representations at each transformer layer in HELIX (Data S3[13]). HELIX performs layer-wise principal-component analysis (PCA) transformations on hidden states (e.g., Pythia 70m's dimension $d = 768$) to analyze embedding space topology. Hidden states were defined as

$$H^{(\ell)} = \begin{bmatrix} h_1^{(\ell)} \\ h_2^{(\ell)} \\ \vdots \\ h_n^{(\ell)} \end{bmatrix} \in \mathbb{R}^{n \times d},$$

where $h_i^{(\ell)}$ is the d-dimensional embedding of residue i at transformer layer ℓ, and n is sequence length. The hidden state matrix at layer ℓ, $H^{\ell} \in \mathbb{R}^{n \times d}$, was decomposed by singular value decomposition:

$$H^{\ell} = U^{(\ell)} \Sigma^{(\ell)} \left(\mathrm{V}^{(\ell)}\right)^{\mathsf{T}}, U^{(\ell)} \in \mathbb{R}^{n \times n}, \Sigma^{(\ell)} \in \mathbb{R}^{n \times d}, \mathrm{V}^{(\ell)} \in \mathbb{R}^{d \times d}.$$

We then projected each residue onto the first two principal axes:

$$Z^{(\ell)} = H^{(\ell)} V^{(\ell)}_{:,1:2} \in \mathbb{R}^{n \times 2}, z_i^{(\ell)} = Z_{i,:}^{(\ell)}$$

and plotted the resulting 2D coordinates $z_i^{(\ell)}$ for all residues (Figure 7). Here, $V^{(l)}_{(:,1:2)}$ is the $d \times 2$ matrix containing the first two right-singular vectors (principal axes), so that the residue-wise coordinates are $Z^{(l)} = H^{(l)} V^{(l)}_{(:,1:2)}$ and Z_i^{l} is the corresponding 2D point for residue i.

This transformation allowed us to observe how amino acid embeddings spread and clustered as network depth increased.

Thus, the HELIX explainer extracts hidden-state vectors from every layer and reduces them via PCA to track how amino acid features evolve through the network (Figure 7). We observed a progressive increase in representation complexity from layer 0 to layer 5—with tight clustering by layer 3 and maximal separation around layer 5—followed by a slight convergence at the final layer, indicating refined, class-specific feature encoding. Notably, methionine consistently occupies a central position in these PCA projections (reflecting its role as an initiator residue), whereas cysteine appears at the periphery, hinting at its unique structural and redox functions in proteins.

The distinctive trends we observed that were unique to cysteine and methionine are peculiar considering the evolutionary significance these residues represent. These two sulfur-containing amino acids exhibited unique representation patterns across layers (Figure 7), potentially reflecting their special roles in protein folding and redox cellular processes. Methionine consistently appeared at the center of amino acid clusters in layer-wise projections in PCA plots, aligning with its known function as the initiator of protein synthesis. Notably, cysteine, which also contains sulfur, exhibited atypical trends in these PCA plots, occupying peripheral projections. This anomaly may be linked to cysteine's crucial role in redoxome enzymes, which are fundamental to a species' antioxidant capacity.[37–39] Given that antioxidant capabilities influence evolutionary trajectories,[40–42] these distinctive cysteine influence patterns may reflect underlying lineage-specific adaptations and warrant further investigation.

Together, the layer-wise PCA trajectories and the per-position influence distributions illustrate how LA^{4}SR rapidly locks onto its classification decision once the key internal sequence features have been observed. Traditional phylogenomic methods have struggled to elucidate the complex interplay between environmental pressures and lineage relationships. Meanwhile, our results suggest that deep neural networks can capture and unravel these intricate long-term interactions.

Validation across diverse genomic datasets

In addition to testing on our holdout sets composed of unseen sequences from the training taxa (Figure 2A), we validated our approach using data from new assemblies from seen species (Figure S7), contaminated assemblies from unseen genera (Figure S7), and clean assemblies from unseen genera (Figure 8). These applications were crucial for differentiating between natural algal sequences, which can sometimes appear bacteria-, archaea-, or fungi-like, and true contaminant species—a distinction vital for accurately assessing the genetic makeup of algal samples and identifying potential contamination in sequencing data.

To show that LA^{4}SR performs well on new assemblies from seen taxa, we cultured and sequenced ten separate isogenic colonies of *Chlamydomonas reinhardtii* CC-1883. As *Chlamydomonas* has a high natural mutation rate,[43–45] the genomic sequences of these colonies were not expected to be exact matches for the reference and were not included in the training datasets. Of these, nine were sequenced with Illumina 150-bp paired-end short reads and one with PacBio HiFi reads and DoveTail Hi-C to generate a complete, axenic reference assembly (Figure S7, NCBI Bioproject SAMN44618602).

We compared the performance of LA^{4}SR with BLASTP for two axenic *Bigelowiella natans* cultures sequenced independently from geographically distant labs and published in different studies.[17,46] These are excellent representatives of bona fide genome assemblies from an outlier alga relatively distant from all other microalgal lineages. These genomes yielded very similar assemblies with relatively high counts of bacterial-like sequences as determined using LA^{4}SR as well as BLASTP.[14] Out of 9,749 and 9,571 total queries with BLASTP hits, 1,005 and 1,035 sequences had the highest number of hits in bacteria (Table S2). These results (Figure S7 and Table S1) bring the maximum expected bona fide algal sequences registering as false positives per genome up to 10.56% ± 0.36%.

To address whether this high false-positive rate in this lineage is because of its unique multi-endosymbioses or nucleomorph genome,[17,47] we downloaded the *Bigelowiella natans* nucleomorph genome (accession numbers GenBank: DQ158856, DQ158857, and DQ158858 for chromosomes 1, 2, and 3). We found that 87.62%, 73.79%, and 85.33% of proteins from the nucleomorph chromosomes were predicted as contaminants. Because the nucleomorph genome only contains <1% of all coding sequences for *Bigelowiella*, its composition was not expected to influence predictions up to 10%; the false positives here may be resulting from genes of nucleomorph evolutionary origin that had already transferred to the nucleus. Ultimately, false positives in any given microalgal genome most likely result from evolutionary distance and clade under-representation.

The ultra-sensitive Diamond BLASTP[11] detection of contaminants suffered from low recall rates, with no hits found for 65.3% ± 0.25% of input sequences from all genomes tested (n = 166, E = 0.00001, see Table S1). Similarly low recall was seen in NCBI BLASTP$^+$ when tested on representative sequences (Figure 1). In contrast to BLASTP, algaGPT-nano made predictions for >99% of sequences in each input genome (Table S1). Similar near-completely genome-wide predictions could be achieved with LA^{4}SR models[48] on most genomes (~99%). The rate of predicted contamination to algal phylogenetic assignment was comparable in BLASTP and LA^{4}SR, although LA^{4}SR predictions generally had fewer false positives (Figure S7).

Finally, to demonstrate LA^{4}SR performance on genomes from a wide variety of unseen genera, we tested on unseen genera with known contamination (Figure S7) and downloaded 12 genomes from NCBI that had been published after the model was trained or otherwise not included in the training data (Figure 8). These unseen genera included two macroalgal genomes (*Asparagopsis taxiformis* and *Agarophyton chilense*) and one alga lacking chlorophyll (*Prototheca cutis*). The benchmark sets were the original TI-free holdout testing sets, where the algal portion was substituted with tORFeomes from each new algal genome assembly. We found that LA^{4}SR does as well on these new unseen taxa as for the holdout testing sets, with no significant difference between them. The average macro F_1 was 0.9173 for the newly published microalgae species and 0.9212 for the original TI-free holdout set.

In conclusion, the LA^{4}SR AI models demonstrated performance equivalent to or surpassing that of conventional CPU-based methods in contaminant detection while generalizing well to unseen taxa. They had approximately three times the

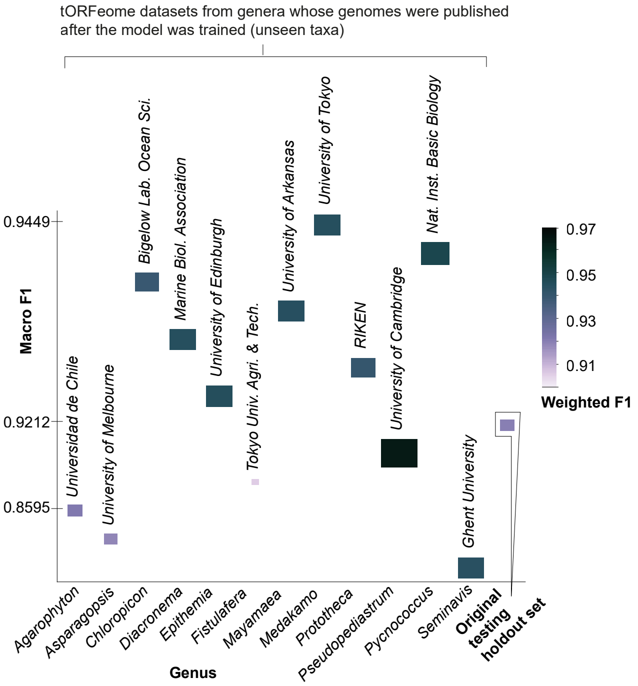

Figure 8. Performance on genomes that were published after the model was trained

See Table S4. The genera shown were not included in the training dataset. Macro F_1 enables direct comparison with the balanced holdout set baseline (n = 10,000 sequences each set) by treating both classes equally, while weighted F_1 reflects performance given the class distribution in each test set. The source institutions producing the assemblies are also shown.

recall rate while operating at speeds orders of magnitude faster. Preceding performance gains, this work shows the successful application of a broad range of open-source AI software to niche, high-level custom biological questions.

DISCUSSION

Our study's application of next-generation language models to biological sequence analysis represents an advancement in computational biology. This approach highlights the potential of transfer learning in bioinformatics, bridging the gap between general language understanding and specific biological sequence analysis. The investigation into TI in LA^{4}SR models' decision-making algorithms revealed intriguing parallels with human language processing. The observed reliance on TI, even when scrambled, mirrors cognitive science findings about the importance of word beginnings and endings in human language processing. This reveals potential similarities between artificial and biological neural networks in sequence interpretation, opening new avenues for interdisciplinary research at the intersection of computational biology and cognitive science. However, the heavy reliance on TI also exposes a potential limitation in current transformer-based models. The bias toward terminal sequences can lead to underemphasizing important internal patterns during training, which is particularly problematic for incomplete or poorly annotated genomes. Our development of TI-free models addresses this issue, demonstrating comparable performance without relying on TI. This approach shows the robustness of LA^{4}SR models[48] and challenges researchers to reconsider the design of attention mechanisms in biological sequence analysis.

The transfer learning paradigm presented in this work capitalizes on the vast amount of knowledge embedded in the model design and the pre-trained weights. The models' pre-existing understanding of complex language structures translates well to the "language" of protein sequences, enabling the capture of subtle patterns and relationships that might be overlooked by traditional methods, especially when dealing with highly divergent or novel sequences. A major focus of the study was investigating the effect of TI in the decision-making algorithms of the models we developed. Deep neural networks, as well as human brain neural networks, weigh the ends of words more than the intermediate letters when deciphering meaning from text.[49–51] This phenomenon is reminiscent of the interactive activation model proposed by McClelland and Rumelhart, which accounts for context effects in letter perception.[52] Research on non-contiguous letter combinations further supports the significance of letter position in word processing.[49,51] Drawing parallels from speech recognition, where recurrent neural networks perform in processing sequential information, we considered how similar principles might apply to protein sequences. Our results suggest that, although TI is processed differently in model-specific manners, the models could learn enough from internal sequence features for accurate classification.

Termini (N and C termini) are often more variable or incomplete in genome annotations: The terminal regions of proteins, especially those that are intrinsically disordered, display higher rates of sequence evolution and variability. These regions are less structurally constrained and more tolerant to sequence changes.[12] Additionally, protein termini are subject to alternative splicing, alternative translation initiation, and post-translational modifications, all of which contribute to their variability and the frequent incompleteness or ambiguity in genome annotations.[15] Furthermore, internal regions of proteins are generally more evolutionarily conserved: structural and functional constraints within the core of proteins mean that these regions tolerate fewer mutations.[12,15] Buried or core residues, which are crucial for maintaining protein stability and function,[2,53] evolve more slowly than surface or disordered regions. This conservation is due to the necessity of preserving the protein's three-dimensional structure and function, which is often dictated by these internal residues.[2,53] Our study leveraged these facts to expand generalizability to a "pan-microalgal" model able to encompass genomic signatures from all microalgal lineages without overfitting. By essentially eliminating TI from the models' knowledge base, we forced learning on "core" patterns in microalgal and contaminant genomes.

The increasing complexity in deep-learning models often results in "black-box" systems that, while powerful, lack transparency and interpretability.[54] This opacity can hinder adoption, confidence, and the extraction of meaningful biological insights. The multi-faceted interpretability framework presented here, combining gradient-based, perturbation-based, and layer-wise analyses, enhances our ability to interpret and validate predictions. Regarding the attribution calculations in each of the programs presented here, HELIX uses purely geometric transformations, DeepLift LA^{4}SR employs back-propagation-based attribution

CellPress
OPEN ACCESS

against reference states, and DMMP utilizes distance metrics in hidden state space combined with statistical thresholding. As AI models become increasingly complex, such interpretability methods will be crucial in establishing confidence and extracting meaningful biological insights. This approach not only predicts and classifies but also elucidates the underlying mechanisms of sequence pattern learning—a crucial feature in bioinformatics, where understanding predictions is as vital as making them.

Although we incorporated more than 77 million distinct sequences in the training data, the biological sequence data available for several representative groups remains sparse. Despite efforts to include diverse algal and contaminant sequences, an under-representation of less-sampled microalgal lineages, including Chromeridia, dinoflagellates, rhodophytes, ochrophytes, and, in general, non-chlorophyte or Bacillariophyta genomes in public databases, limits the scope of the work. These challenges present an opportunity for innovation in database and training data generation and the development of new model architectures and training methodologies, potentially leading to more sophisticated and biologically relevant machine-learning models. The insights gained from comparing TI and TI-free approaches could inform future model designs, potentially leading to more robust and versatile tools for analyzing the complexities of biological systems.

This work contributes to improving the accuracy of algal genome assembly and annotation, enhancing our understanding of microbial evolution and biodiversity, and supporting biotechnological applications that rely on pure algal genetic material. As computational biology continues to evolve, the integration of language models with existing methods and experimental approaches, as exemplified in this study, will be critical in advancing the understanding of the molecular languages of life on Earth, particularly in complex and diverse microbial communities such as those found in algal ecosystems. Combining powerful deep-learning models with robust explainability techniques paves the way for more transparent, reliable, and biologically meaningful microbial genomics analysis.

METHODS

Open-source model architectures

The LA^{4}SR framework integrated several open-source software packages and models. We used low-rank adaptation (LORA)[55] and quantized low-rank adaptation (QLORA) for parameter-efficient post-training and Mamba as an alternative to transformer-based[56] architectures. The Hugging Face (https://huggingface.co) transformers library facilitated implementation and post-training of the open-source models, while Bitsandbytes enabled quantization and memory-efficient training. For model analysis and interpretability, we developed custom gradient extraction programs based on SHAP[25,57] (https://github.com/shap/shap, see Figure S4), Captum,[23] and DeepLift. We post-trained LLMs including Mistralai/Mistral-7B-v.0.1 (https://huggingface.co/mistralai/Mistral-7B-v0.1), as well as various Mamba pre-trained models from the Hugging Face model hub. We used the S6 Mamba[58,59] and transformer-based Mistral,[60] GPT variants (nanoGPT[61] and GPT-NeoX[26]), ByT5,[62] DistilRoBERTa,[33–35] MiniLM,[63] BLOOM-560m,[64] and Pythia (7B and 70m)[65] for pre-training and post-training.

Uniformity in coding was ensured using AutoModel.[66] This system automatically handled model-specific configurations through the from the _pretrained() method, which loads pre-trained weights from either local storage or Hugging Face's model hub. The initialization process involved three main components accessed through their respective Auto classes (AutoConfig, AutoModel, and AutoTokenizer), maintaining consistent configurations, model weights, and tokenizers while preventing component incompatibilities. The AutoModel approach facilitated the architecture-agnostic implementation of LA^{4}SR, where model-specific details are abstracted away through the AutoConfig class. This class automatically selected appropriate configurations based on the model type, handling architecture-specific parameters such as model dimensions, the number of layers, and attention heads according to each pre-trained model's specifications.

The DistilRoBERTa[33–35] architecture was the only bidirectional model as well as the only model using sentence transformers (https://sbert.net/). PyTorch[36] was used as the primary deep-learning framework for implementing custom datasets, data loaders, and training pipelines. Additionally, we developed custom Python scripts for data pre-processing, model training, and analysis, including a novel motif-based explainer pipeline for model interpretability. Training was performed on an HPC cluster using NVIDIA[67] (Santa Clara, CA, USA) A100, V100, and H100 GPUs.

Implementing parameter-efficient post-training techniques, such as LORA and QLORA, proved crucial for post-training larger LLMs. The low-rank approach allowed us to efficiently fine-tune large models. The relatively low level of input data needed to make the LLMs perform well (compared to sub-1B models, i.e., LMs) suggests that our primary classification task (i.e., algal vs. bacterial) is a low-rank problem with a small number of extremely complex and important features that can be rapidly learned by LLMs as shown in our post-training of Mistral 7B (Figures 1 and S2). These influential features are the proteomic evolutionary adaptations characterizing organisms from each group.

Genomic data acquisition and preparation

Complete proteomes were obtained from publicly available genomic data for species across 12 algal and plant phyla (Rhodophyta, Chlorophyta, Streptophyta, Cryptophyta, Haptophyta, Ochrophyta, Myzozoa, Euglenophyta, Heterokonta, Cercozoa, Chromerida, and Pelagophyta). These datasets—generated primarily by our large-scale ALG-ALL-CODE project[46]—include high-quality assemblies of nuclear, chloroplast, and mitochondrial genomes, as well as coding sequences from all known extrachromosomal compartments: secondary-plastid nucleomorphs (in cryptophytes and chlorarachniophytes), plastid minicircles (in dinoflagellates), natural plasmid-like elements (in diatoms and certain green algae), and viral or episomal inserts. For training, we assumed that every sequence assembly deriving from a given microalgal species was authentic (i.e., microalgal rather than contaminant), and thus our "complete" proteomes incorporate every protein-coding gene from nuclear, chloroplastic, mitochondrial, and other intracellular plasmid or episomal DNA. Non-algal sequences were downloaded from the non-redundant protein sequence database at NCBI (nr, ftp://ftp.ncbi.nlm.nih.gov/blast/db/FASTA/nr.gz).

We prioritized bacterial contaminants due to their pervasive presence and resilience throughout the entire sequencing workflow—from cultivation in photobioreactors or natural ponds through DNA extraction and sequencing library preparation. Bacteria frequently exhibit tight associations with algal cells, either intracellularly or on the cell surface, enabling their DNA to persist even after rigorous washing and standard lysis protocols. Moreover, bacterial DNA contamination can originate directly from extraction reagents and kits, further complicating the distinction between genuine algal sequences and contaminants.

While bacteria represented the primary focus due to their prevalence and documented interference with genomic workflows, we also evaluated fungal contaminants because fungi produce resilient spores and form stable associations that withstand typical DNA extraction protocols. Similarly, archaea and viruses were included in the study owing to their documented occurrences in algal cultures and their potential to significantly influence genomic analysis outcomes. Viruses, though often overlooked, can integrate genetic material or persist in cultures undetected, posing risks of misannotation and genomic assembly errors.

Non-microalgal protists, despite their ecological relevance as grazers and potential intracellular contaminants, were not included due to their generally larger cell size and relative ease of visual identification under microscopy, reducing their likelihood of persisting unnoticed throughout cultivation and sequencing processes. Additionally, protists' closer phylogenetic proximity to algae could complicate genomic analysis, thus warranting separate, specialized investigations distinct from our bacteria-centric study.

In silico proteome generation

For all species, we used SNAP[68] gene prediction software to identify coding sequences and generate *in silico* translated proteomes (https://github.com/KorfLab/SNAP). This method was previously found to generate the best predictions, overall, for each microalgal lineage.[46] The SNAP command used was "snap A.thaliana.hmm $GENOME.fa -residue $GENOME.aa.fa -tx $GENOME.tr.fa -gff -quiet -name $GENOME > $GENOME.gff." tORFeomes from ~120 microalgae species were first screened for potential contaminants using BLAST-based detection. Only tORFeomes with less than 10% potential contaminant sequences were retained for further analysis.

Training data preparation

Algal and contaminant (either mixed with fungi and archaea or purely bacterial) sequences were combined to form the training/evaluation split training datasets at a 1:1 ratio, with 58,650,525 (6,375,974,800 characters) and 17,880,279 (4,224,680,663 characters) sequences in the TI-free and TI-inclusive training datasets (Data S1).[13] tORFeomes from more than 100 microalgae species were first screened for contaminants using traditional BLAST-based detection, and tORFeomes passing a 10% filter were maintained.

This threshold was chosen based on results we obtained from two independently sequenced,[17,46] bona fide chloroarachniophyte genomes. The greatest quantity of contaminant-like sequences that could be predicted from known clean algal genomes from the BLAST[14]/Blast-limiting eukaryotic genomes, heuristically (BLEACH)[46] approach was from the chloroarachniophyte species (*Bigelowiella natans*),[17,46] at ~10% (Table S2). Passing genomes were divided into training, evaluation, and test sets based on the training regime (TI-inclusive or TI-free) used. Our study examined the influence of sequence length on the decision algorithm in both pre-trained and post-trained models and explored how token masking was applied across different model configurations.

The training data preparation workflow consisted of large-scale protein/amino acid sequence processing and LLM training, with several distinct processing stages. Sequence pre-processing was performed using a custom pipeline (see Data S2)[13] that handled segmentation, labeling, and format standardization. The core training infrastructure included distributed training scripts for multi-GPU setups alongside specialized training implementations for different model architectures. Post-training capabilities were implemented using fastLoRA-train.py and configurations specified in Data S2.[13] We trained and inferenced models with and without TI (Figure 2). One class of datasets used full-length sequences (TI-inclusive), while the other scrambled start and stop site information (TI-free, Figure S1). These two approaches were drastically different, each presenting different caveats and advantages. For full-length sequences, a "<" was added to the end of each sequence to indicate the switch to sample for a label instead of a protein sequence. However, in testing, labels could usually be generated with sequence prompts alone without the need for additional characters.

To generate TI-free training data, we first stripped all FASTA headers and removed every newline character, yielding one continuous "unisequence." That unisequence was then segmented into fixed 100-amino-acid fragments (printed at 100 residues per line) and fully shuffled, thereby eliminating any terminal or gene-boundary information. This process produces chimeric, non-natural sequences while ensuring each fragment remains exactly the same length, which benefits both training and inference by removing length as a predictive cue. The approach can be summarized as

$$f_j = \left(a_{(j-1)l+1,\ldots,}a_{jl}\right), l = 100, j = 1, \ldots, \lfloor L/l \rfloor .$$

Each 100-residue fragment retained its original group label and was concatenated into a single master dataset. After a final shuffle, we split that dataset into training and testing sets under two schemes—a binary classification (algal vs. bacterial) and a four-way classification (algal, bacterial, fungal, and archaeal)—each preserving a 1:1 ratio of algal-to-contaminant fragments. By forcing the model to rely solely on position-independent amino acid patterns (core signatures), this TI-free approach rigorously tests its ability to distinguish classes without any TI bias. To streamline data feeding, we built a custom PyTorch dataset and paired it with a DataLoader optimized for throughput: we used batch sizes of 32–128, enabled shuffling, launched 4–8 worker processes for asynchronous loading, and pinned memory to speed up GPU transfers. Prior to batching, every sequence is tokenized at either the character level (TI-free) or subword level, converted into PyTorch tensors, and uniformly padded to 100 characters. This fixed-length format simplifies

memory management, aligns neatly with transformer attention windows, and reduces per-batch pre-processing overheads.

Although LA^{4}SR had near-perfect recall for bacterial contaminants (and strong performance on fungal and archaeal sequences), it does not yet explicitly model eukaryotic protist contaminants—such as ciliates, amoebae, flagellates, labyrinthulids, or zooplankton—which, by virtue of being closer to algae phylogenetically, may exhibit subtler sequence differences and thus represent a potential blind spot in contamination detection. Finally, during fine-tuning, we varied learning rates from 1×10^{-4} down to 1×10^{-5}, experimented with batch sizes between 8 and 96, and used context windows ranging from 128 to 4,096 tokens; these hyperparameter choices consistently delivered robust downstream classification performance.

Tokenization and model versatility

A key factor in comparing diverse LM architectures is minimizing confounding influences from tokenization schemes. For each architecture—whether nanoGPT, GPT-NeoX, Mistral, or others—we evaluated multiple tokenizers (including native subword, hybrid byte-pair/byte-level, byte-level, and pure character-level encodings). Across these experiments, overall classification accuracy varied by less than 1%, indicating that model performance was largely agnostic to tokenizer choice. In practice, we selected final model variants based strictly on their performance metrics, without privileging any particular tokenization strategy.

To support consistent interpretability and avoid compatibility issues with explainability tools, we generally adopted a byte-level tokenizer (ByT5) when running attribution analyses and benchmarking across architectures. Byte-level tokenization eliminates out-of-vocabulary concerns, ensures uniform input granularity, and simplifies attention-window alignment—critical for visualizing residue-level attribution heatmaps. Nonetheless, final model variants were chosen purely on performance: the top TI-free model employed the byte-level tokenizer, whereas the top TI-inclusive model used its native GPT-NeoX subword tokenizer. We also preserved each model's native tokenizer for its original full-length training data, thus maintaining pre-trained vocabulary, leveraging subword motifs linked to protein domains, and ensuring seamless compatibility with model weights.

In the "TI-free" scrambled fragment experiments, pure character-level encoding was applied to guarantee that every input consisted of exactly 100 tokens. By removing subword segmentation and fixed-length cues, this strategy forces models to rely exclusively on intrinsic amino acid patterns. This uniform input format also streamlines batching and downstream analyses by decoupling classification performance from variable token lengths or subword biases.

Training dynamics and optimization

Training experiments across a variety of LA^{4}SR architectures revealed consistent patterns in convergence speed, generalization, and batch-size sensitivity. Models typically attained strong performance within 20,000–50,000 training steps depending on size and initialization strategy. Batch size proved critical for optimizing model accuracy and stability. While larger batch sizes ($\geq$512) occasionally succeeded, they consistently degraded generalization performance, leading to sharper minima and lower downstream F_1 scores. Optimal results were with batch sizes between 64 and 96, particularly for models trained on A100 GPUs. This finding aligns with prior observations in deep-learning literature that extremely large batches can compromise model robustness.[69]

Training efficiency scaled favorably across architectures, with no major instabilities observed even at large context window sizes (up to 4,096) or during post-training of parameter-efficient models (e.g., with LoRA or QLoRA adaptors). Combined with the rapid emergence of domain knowledge, these training dynamics accelerated learning, supporting LA^{4}SR's scalability to larger datasets.

Generation and use of new, real-world sequencing examples

The foundational datasets we used for training consisted of real-world sequencing experiments, either at the whole-genome or large-scale whole-genome level. Here, to validate LA^{4}SR models[48] with new real-world data, we performed both short-read and long-read sequencing on additional axenic and xenic algal cultures. Ten isogenic colonies of *Chlamydomonas reinhardtii* strain CC-1883 (cw15 NIT$^+$ mt$^-$; https://www.chlamycollection.org/product/cc-1883-cw15-nit-mt/) were isolated and cultured under standard growth conditions. Nine colonies were sequenced using NovaSeq 6000 (Illumina, San Diego, CA, USA) with 150-bp paired-end reads. For the tenth colony, we generated a complete reference assembly using a combination of Pacific Biosciences (PacBio, Menlo Park, CA, USA) HiFi long reads and Hi-C chromatin capture data. Cultures were maintained in Tris-acetate-phosphate medium at 25°C under continuous light (100 μmol photons m^{-2} s^{-1}) with constant shaking at 150 rpm. Genomic DNA was extracted using a protocol optimized for algal cells as indicated in Nelson et al..[70] For Illumina sequencing, DNA libraries were prepared using the NEBNext Ultra II DNA Library Prep Kit following the manufacturer's instructions. Paired-end sequencing was performed with a target coverage of 50× per sample. For the PacBio HiFi sequencing, high-molecular-weight DNA was extracted and size selected for 15- to 20-kb fragments. Hi-C libraries were prepared using the DoveTail (Sydney, Australia) Omni-C Kit following the manufacturer's protocol. This produced a chromosome-level assembly that served as a clean, axenic reference. For validation, we also analyzed previously published genomes from isolates of *Bigelowiella natans*, known for containing bacterial-like sequences due to their evolutionary history,[17] from two separate whole-genome sequencing projects. We evaluated potential contamination using three approaches: traditional BLAST-based detection using the BLEACH pipeline, ultra-sensitive Diamond BLASTP (E = 0.00001), and LA^{4}SR models, including algaGPT-nano. Our analyses showed that several of the genomes would not finish after runtime of 1 month with NCBI BLASTP, effectively precluding further investigation with this tool for our purposes. Ultra-sensitive Diamond[11] BLASTP commonly finished jobs in approximately 2 days or less, and most LA^{4}SR jobs finished in runtime of under 1 h.

The results from axenic cultures established baseline false-positive rates for bacterial-like sequence detection. Analysis of the *Bigelowiella natans* genomes revealed that up to 10.561% ± 0.357% of bona fide algal sequences may present bacterial-like characteristics, providing an important benchmark for model evaluation. This reference point helped calibrate

expectations for LA^{4}SR model precision, particularly when analyzing organisms with complex evolutionary histories. Ultra-sensitive Diamond BLASTP showed low recall rates, failing to classify 65.3% ± 0.25% of input sequences from the tested genomes (n = 166) even in the ultra-sensitive mode. In contrast, LA^{4}SR models[48] showed near-complete sequence classification (~99%) while maintaining accuracy comparable to or exceeding BLAST-based methods. The real-world validation demonstrated that LA^{4}SR models could effectively distinguish between genuine bacterial contamination and algal sequences with bacterial-like characteristics, a crucial capability for accurate genome analysis. The newly sequenced genomes shown in Figure S7, including a new Hi-C/PacBio reference assembly for the *Chlamydomonas reinhardtii* strain CC-1883, are available from Data S4[13] and NCBI (SAMN44618602).

Computational resources and energy consumption

We trained ten LA^{4}SR models (70m to 12B parameters) in parallel, each on its own NVIDIA A100 GPU: models under 300m parameters were pre-trained from scratch via architecture mimicry using AutoModel from Hugging Face configurations in mixed-precision (FP16), while larger models (≥300m) were fine-tuned using low-rank adapters (LoRA) and 8-bit QLoRA for parameter-efficient adaptation. Mamba[58,59] S6 models required 32FP. Each model trained continuously for a maximum of 4 days (≈96 GPU-hours per model; ≈960 GPU-hours total), with each A100 drawing ~400 W (~38.4 kWh per run; ~384 kWh aggregate; ≈500 kWh including a power usage effectiveness [PUE] of 1.3), and gradient checkpointing was used to cut memory footprint and energy consumption by ~15%.

Computational analysis of *in silico* proteomes

Proteome data were processed in comparative phylogenetic analyses (Figure 1). Sequence manipulation was performed with BioPython,[71] tabular data were handled with Pandas,[72] statistical computations employed SciPy,[73] and all visualizations were generated via Matplotlib[74] and Seaborn.[75] For each phylum we computed three tiers of metrics: (1) phylum level, including total protein count, species count, average proteins per species, and total amino acid residues; (2) protein level, namely the full distribution, median, and mean of individual protein lengths; and (3) amino acid level, i.e., the percentage composition of each of the 20 standard residues.

Protein-length distributions were displayed with violin plots embedding boxplots (showing median, quartiles, and extrema); values above the 99th percentile were omitted from the plots for clarity but retained in all analyses. To enable side-by-side comparison of disparate metrics, each value was min-max normalized by its observed maximum. A composite, dual-panel figure juxtaposed a phylum dendrogram (left) with aligned bar plots of normalized total proteins, proteins per species, and average protein length (right).The code was executed in Python 3.8 with SciPy 1.7.1, and figures were exported as publication-quality scalar vector graphics using Matplotlib 3.5.0 and Seaborn 0.11.2.

Technical evaluations of LA^{4}SR and traditional architectures

To assess the computational requirements of LA^{4}SR models, we calculated several key metrics. These included the number of parameters (Params), multiply-accumulate operations (MACs), floating-point operations (FLOPs), and floating-point operations per second (FLOPS). We distinguished between forward-propagation FLOPs (fwd FLOPs) and backward-propagation FLOPs (bwd FLOPs), assuming that the default model back-propagation requires twice the computation of forward propagation. We calculated these metrics for both the forward pass alone (fwd) and combined forward and backward passes (fwd + bwd). The total training parameters were computed, along with fwd MACs, fwd FLOPs, fwd + bwd MACs, and fwd + bwd FLOPs. All values were calculated and reported in appropriate units (m for millions and B for billions) (Table S1).

The larger models we tested reached higher accuracy with less training data (Figure 2). For instance, a QLORA-post-trained Mistral 7B model reached an F_1 score of ~88 on algal/bacterial classification after only 2,000 training steps, compared to roughly 20,000 steps required for smaller models trained from scratch. The TI-free approach, using fixed-length 100-amino-acid sequences, had higher inference speeds than full-length sequence processing. Memory optimization techniques, including quantization and gradient accumulation, enabled efficient training and inference even on limited hardware. This scalability and flexibility make LA^{4}SR adaptable to various research environments, from individual workstations to high-performance clusters, offering a fast and accessible tool for microbial genomics and protein sequence analysis across different computational settings.

One of the primary motivating factors for our work was instilling the capacity to work with the latest GPU technologies[67] into bioinformatics software. The A100 GPU demonstrated a 200–400× improvement in floating-point operations per second compared to single-core CPU performance in double-precision calculations. For example, the performance gap between a GPU, specifically the NVIDIA A100[67] used here in most calculations, and a typical high-performance CPU core (e.g., an AMD EPYC 7742 64-core processor at 2.25 GHz) for a BLASTP-like algorithm is monumental and is due to several technical factors. At its core, this difference stems from the fundamental architectural disparities between these two types of processors. The A100 GPU has 6,912 CUDA cores capable of massive parallelism, delivering up to 19.5 TFLOPS in double precision and 156 TFLOPS in single precision.

This raw computational power, coupled with a staggering 1.6 TB/s of memory bandwidth, stands in stark contrast to a typical high-performance CPU core, which might offer 50–100 GFLOPS and 50–100 GB/s of memory bandwidth. This translates to the GPU potentially performing 200–400 times more floating-point operations per second than a single CPU core in double precision, and even more in single precision. We tested speed using translated coding sequences from whole algal genomes (n = 166) as queries for ultra-sensitive Diamond BLASTP[11] and the LA^{4}SR model algaGPT (Table S1). The algaGPT model was 82.9 times faster, on average, than ultra-sensitive Diamond BLASTP. Genomes generally finished in under 1 h with algaGPT on an NVIDIA A100 GPU, while Diamond BLAST runs took 1–2 days using an AMD EPYC 7742 64-core processor at 2.25 GHz. The sensitive mode of Diamond BLAST was previously found to be 2,000 times faster[11] than NCBI BLAST; thus, using NCBI BLAST for these genome-level comparisons was not feasible. We thus used the estimate from Buchfink et al.[11] to

compare the speed of our models to NCBI BLAST, translating to a 16,580× speedup of algaGPT, on average, compared to NCBI BLAST.

This extrapolation from the Diamond BLAST publication was close to our experimental values for runtime speedups over NCBI BLASTP+ (Figures 1E and 1F; Table S1). To quantify LA4SR's speedup over NCBI BLASTP+, we wrote a lightweight Python wrapper (see supplemental code in Data S5) that (1) splits a multi-FASTA into individual queries, (2) invokes BLASTP on each with user-specified -outfmt, -evalue, and thread count, and (3) records per-query runtimes into a TSV. Temporary single-sequence FASTA files are written into blast_tmp/, then submitted either locally or via an SLURM array (28 CPUs, 90 GB RAM, 96 h) where each task reads a filename from filelist.txt (Data S3).[13] In normal (non-benchmark) mode the same script simply streams full blastp output exactly as if it were invoked directly. All timing data were aggregated and plotted in Figures 1E and 1F (and Table S1), demonstrating a median 10,701× speedup over NCBI BLASTP+ (AMD EPYC 7742) and ~83× over Diamond BLASTP+ on identical query sets.

Our analyses revealed large variations in both computational requirements and potential runtime performance. LLMs (i.e., >1 billion [1B] parameters) demonstrated the highest computational intensity. For example, the Mistral 7B-1000 model could run at 1,830 GFLOPS and 912.8 GMACs, coupled with the largest parameter count of 7B, suggesting it would likely have the longest runtime among the models studied. Conversely, the 19m TI-free LA4SR model with a Pythia 70m architecture had the lowest computational needs (5.09 GFLOPS and 2.54 GMACs) and a compact parameter count of 19.31m, indicating potential for faster execution. The sentence-transformers-all-distilroberta-v1-dualityFT50000[76] and Byt5-Prophecy100s-30000[77] models exhibited similar computational profiles (approximately 22 GFLOPS and 11 GMACs), suggesting comparable runtimes, despite slight differences in their parameter counts (82.12m and 86.14m). The 70m TI-free LA4SR model with Pythia 70m architecture occupied a middle ground regarding terms of both computational intensity and potential speed, with 11.63 GFLOPS, 5.81 GMACs, and 70.43m parameters. Notably, the TI-inclusive LA4SR model based on gpt-neo-125m architecture showed moderate computational requirements (32.24 GFLOPS and 16.11 GMACs) but had a larger parameter count of 125.2m, suggesting a balance between model size and runtime efficiency. These metrics provide insights into the trade-offs between model complexity and potential execution speed, crucial factors in selecting models for various applications with different performance requirements.

For a more granular analysis, we broke down the calculations for each module in the model. For each module, we reported its parameters, percentage of total parameters, MACs, percentage of total MACs, FLOPS, and percentage of total FLOPs (Table S2). Some modules may use torch.nn.module or torch.nn.functional to compute logits (e.g., CrossEntropyLoss), which are not counted as submodules in our calculations. This can result in a discrepancy between a parent module's MACs and the sum of its submodules' MACs. Additionally, we acknowledge that the number of floating-point operations is a theoretical estimation, which may result in calculated FLOPS exceeding the maximum system throughput.

Inference for algal/contaminant sequence discrimination in genomic data

Our primary objective was to leverage generative modeling techniques to differentiate between algal and contaminant genomic sequences, where the predominant contaminants come from various bacterial lineages. This task is crucial in algal genomics, where the accurate identification of source material is essential for downstream analyses and applications.

Models under 300m parameters were trained from scratch by extracting or mimicking the underlying architecture configuration from the Hugging Face model, and post-trained models used the weights from the pre-trained models to start. According to our BLAST results that identified substantial portions of bona fide algal sequences as bacteria-like (up to ~10% in some species; see Table S1), we did not expect the precision of LA4SR models[48] to reach higher than 90% when inferencing on algal sequences (Table S2). However, in several instances, this metric was surpassed (Figure 2). Reasons for the superior performance of some LA4SR models compared to the BLAST-based baseline estimates include biases in the input sequence and biases in the determination of the 90% algal precision threshold.

By prompting each sequence (or 100-residue fragment) to generate a single tag token and simply tallying those outputs, we could classify without any extra head or fine-tuning. Next-token tag aggregation proved both simple and effective: by prompting each sequence (or fragment) to emit a single tag token and tallying those predictions, we obtained high-fidelity discrimination without any additional classifier layers. This tag-counting scheme leverages the model's intrinsic generative capacity, preserves inference efficiency, and seamlessly integrates with our existing pipeline. The approach can be summarized as

$$p(x_t|x_{<t};\theta) = \mathrm{softmax}(Wh_t + b)_{x_t},$$

$$L(\theta) = -\frac{1}{N}\sum_{i=1}^{N}\sum_{t=1}^{T_i}\log p\left(x_t^{(i)} \mid x_{<t}^{(i)};\theta\right).$$

Applying it across diverse holdout datasets (Figure 2) confirmed its robustness, underscoring that next-token outputs alone can drive accurate algal vs. contaminant classification.

For testing, we used either batches of 1,000, 10,000, or ~300 million sequences per set, representing small, standard, and exhaustive testing datasets not used in training, corresponding to either TI-inclusive and TI-free algal, bacterial, fungal, and archaeal sequences not used in training (Figure 2). For multi-class comparisons, we calculated macro F_1 as the unweighted average of per-class F_1 scores: macro F_1 = (F_1_algae + F_1_bacteria)/2. This metric treats both classes equally regardless of sample size. We also calculated weighted F_1, which weights each class's F_1 score by its support: weighted F_1 = (n_algae × F_1_algae + n_bacteria × F_1_bacteria)/(n_algae + n_bacteria), where n_algae and n_bacteria are the number of samples in each class. Macro F_1 enables direct comparison with balanced test sets, while weighted F_1 reflects real-world performance given class distributions.

We reason that sensitivity to learning outlier algal sequence examples by the model determines a domain of accuracy specific to clades with members showing more chimeric genomes, such as dinoflagellates, Chromeridia, Myzozoa, and Cercozoa. While the BLAST-based contaminant detection analysis

registers ~10% contamination in the bona fide *Bigelowiella natans* proteome, models were trained with all its sequences labeled as algal sequences. Thus, discriminating models based on higher-diversity sequences can help inform more holistic decisions regarding lineage.

We screened our TI-free 10,000-sequence algal and 10,000-sequence bacterial holdout sets for any exact duplicates against the training database by concatenating each FASTA record into a single "bare" sequence string (i.e., without wrappings) and performing fixed-string, whole-line matching. This process identified 204 overlapping sequences (<1.1% of the 20,000 total holdout), which were then removed to produce a cleaned holdout set of 19,796 sequences (9,934 algal and 9,862 bacterial). We recomputed all classification metrics on both the original and cleaned holdout sets: overall accuracy was 92.12% vs. 92.13%, macro F_1 was 0.9212 vs. 0.9213, and per-class precision/recall differences remained below 0.1%. The near-identical performance demonstrates that this small amount of exact-sequence overlap had no meaningful impact on our results, and thus our test split can be considered effectively clean.

The commands used for the inference results for TI-free model metrics shown in Figure 2 were 'PYTORCH_CUDA_ALLOC_CONF=expandable_segments:True && python infer-ByT5tok-attn.py $LINE BactTop10000-10holdout >> "$cleaned"_BactTop10000-10holdout' and 'PYTORCH_CUDA_ALLOC_CONF = expandable_segments:True && python infer-ByT5tok-attn.py $LINE AlgalTop10000-10holdout >> "$cleaned"_AlgalTop10000-10holdout' for the contaminant and algal holdout sets. These commands generated next-token predictions, which were then screened for tags corresponding to either group. The metrics were generated from the two holdout inference result files, counting algal and bacterial markers to tally true/false positives and negatives in each. Then, precision, recall, and F_1 scores for both algal and bacterial classifications were computed. The TI-inclusive models were evaluated similarly except with sets of 1,000 full-length sequences representing the full spectrum of contaminant groups.

Well-performing models were expected to achieve high precision on unknown bacterial sequences because, in general, bacterial genomes do not contain algal-like sequences. Bona fide bacterial sequences called "algal" by the model represent false negatives that may have been learned due to their similarity to bacterial-like algal sequences. These results represent the heaviest caveat of LA^{4}SR; ideally, the stringency of bona fide bacterial sequence calls should be paramount. Still, some of the LA^{4}SR models[48] approximate the target stringency at the cost of slightly lower algal precision (Figure 2). We used mixed-precision training (FP16) with gradient checkpointing to optimize memory usage. We included the AdamW optimizer with a weight decay of 0.01 and an initial learning rate of 1×10^{-4} coupled with a cosine learning rate scheduler incorporating 2,000 warmup steps.

The implementation includes robust error handling for invalid model configurations, missing or corrupted model weights, incompatible tokenizer configurations, and resource constraints during model loading. This ensures system stability and provides meaningful error messages for debugging and maintenance. All models present a unified interface for forward passes, state management (training/evaluation modes), and weight loading and saving despite their architectural differences. When necessary, the configuration can be modified for specific downstream tasks while maintaining architectural compatibility, providing flexibility for various applications while preserving the integrity of the underlying model architecture.

Training was run for 0.02–3 epochs with a per-device batch size of 16–96 and gradient accumulation steps of 8–32 to simulate larger batch sizes while managing memory constraints. To monitor training progression, we integrated Weights & Biases (wandb.ai) logging and tracking key metrics including training and validation losses. The amount of training for optimal results (F_1 > 90) was generally around this level for 100–200m parameter models. In contrast, the smaller Pythia 70m needed longer training times and more input data, and the larger models (>300m) needed less training on average. The high F_1 scores for both algal and bacterial sequences reached in many LA^{4}SR models[48] indicate that they have learned to capture the distinctive features of each group, making it a valuable tool for microbial community analysis and protein function prediction.

Balancing algal and bacterial precision was not necessarily a function of the input training dataset size or training time. For example, a GPT-NeoX-125m model trained with a ByT5 tokenizer exhibited a "sweet spot" of model performance at 30,000 training steps (batch size = 64), while the 25,000- and 35,000-step checkpoints showed lower performance. At the 25,000-step checkpoint, the model showed good performance, with an algal F_1 score of 0.7646 and a bacterial F_1 score of 0.6741. The algal precision was notably high at 0.8871, indicating a low false-positive rate for algal sequence identification, but here the recall was low at 0.6720. The model's performance improved significantly by the 30,000-step checkpoint. The algal F_1 score increased to 0.8712, with a remarkable improvement in recall (0.9602) while maintaining a good precision (0.7975). The bacterial F_1 score also increased to 0.8903, with high precision (0.9666) and good recall (0.8253). This checkpoint demonstrated a well-balanced performance for both algal and bacterial sequence classification.

Our GPT-NeoX-125m training instance exemplified the trend of language models often reaching diminishing returns and even degraded performance with extended training regimes. Although recall was maintained, precision wavered after 30,000 steps. The 35,000-step checkpoint showed a slight decrease in performance compared to the 30,000-step mark. The algal F_1 score was 0.8688, with a precision of 0.8008 and a recall of 0.9496. The bacterial F_1 score was 0.8855, with a precision of 0.9566 and a recall of 0.8244. Similar diminishing returns or performance drops were commonly observed in overtrained LA^{4}SR models. The 30,000-step checkpoint model showed the overall best performance on the TI-free benchmark testing set, suggesting that this might be an optimal point for model selection. Thus, exported models were always taken from checkpoints with the highest performance on the testing benchmark datasets (Figure 2). Models are hosted at huggingface.co as described in Table S1 and also in Data S5.[13]

Lineage-specific sensitivity analysis

To probe taxonomic bias and generalizability, we assembled a secondary holdout panel comprising complete proteomes from 30 algal and 25 bacterial species spanning all major lineages—explicitly including under-represented groups such as

CellPress
OPEN ACCESS

Chromerida, dinoflagellates, haptophytes, and cryptophytes (Figures 2 and S3). We ran the same Python metrics pipeline on these genomes, extracting generated token tags per protein and marking missing tags as unknown. By linking each sequence ID to its source species and higher-order taxon via a metadata lookup table, we stratified predictions by lineage and computed metrics for each group.

We further derived a taxon-macro F_1 (the unweighted mean of group F_1 scores) to summarize cross-lineage robustness and generated accuracy-coverage curves per lineage by thresholding tag-generation confidence. This lineage-stratified sensitivity analysis revealed any systematic performance degradation in under-represented algal clades and directly addresses any potential taxonomic bias in model performance.

SHAP-like gradient explainer

SHAP (https://github.com/shap/shap) represents a unified framework for interpreting predictions by combining game theory with local explanations. At its core, SHAP uses Shapley values[78] from cooperative game theory to assign importance values to each feature for a particular prediction. For a model f taking as input a feature set $F = \{1, \ldots, d\}$, the Shapley value Φ_i, of feature i at point x is

$$\Phi_1(x) = \sum_{S \subseteq F \setminus \{i\}} \frac{|S|!(d - |S| - 1)!}{d!} \left[f\left(x_{s \cup \{i\}}\right) - f(x_s) \right].$$

This satisfies local accuracy, missingness, and consistency. This method provides consistent and locally accurate explanations that satisfy important properties like local accuracy and missingness. SHAP stands out for its model-agnostic nature, meaning it can explain any machine-learning model's output, and its ability to provide both local (individual prediction) and global (entire model) interpretations. However, SHAP's computational complexity can be significant, especially for large datasets or complex models, as calculating exact Shapley values requires evaluating all possible feature combinations. We re-engineered the SHAP pipeline to suit our models (SHAP-like explainer; Data S3).[13] SHAP-like values were implemented in our work using our DistilRoBERTa-based[33–35] LA^{4}SR model as an example (Figure S4).

The "SHAP-like explainer" (see Data S3)[13] introduces a new class "CustomShapExplainer" and two new functions (explain (self, input_data, target_tags) and shap_explain(model, tokenizer, test_dataset, num_samples = n)) to extract SHAP-like[25] values for input sequences. Representative output from the custom SHAP-like extraction method is shown in Figure S4. In brief, the method calculates loss toward a target (e.g., the next token) and calculates SHAP-like values with shap-like_values = (input_embeds.grad * input_embeds).sum(dim = −1).detach().cpu().numpy() using the gradients from the input embeddings to designate the influence toward the "algal" or "bacterial" decision. This function directly realizes

$$\phi_i = \sum_j \left[\nabla_{e_i} \mathcal{L}\right]_j e_{i,j}.$$

We demonstrated its application by analyzing how a DistilRoBERTa-based[33–35] LA^{4}SR model processes algal and bacterial queries (Figure S4).

Captum workflows

Captum[23] (https://captum.ai/) offers a comprehensive library of attribution algorithms specifically designed for PyTorch[36] models. It implements various interpretation methods, including integrated gradients, layer conductance, neuron conductance, and several other gradient-based approaches. Captum's main advantage is its deep integration with PyTorch, making it particularly valuable for deep-learning practitioners using this framework. The library excels in providing detailed insights into neural network behavior, allowing attribution at different levels—from individual neurons to entire layers.

To analyze the model's token attributions, we used the LayerIntegratedGradients and DeepLift methods from the Captum[23] library. For each input text, we first tokenized the input and used the model to generate nine new tokens. We then applied LayerIntegratedGradients to compute attributions for the entire generated sequence. The algorithm used 50 integration steps and zero tensors as baselines for both input IDs and attention masks. This process allowed us to quantify the importance of each input and generated token in influencing the model's final token prediction. The resulting attributions were normalized and visualized using a color-coded sequence, with green representing positive attributions and red representing negative ones.

To identify distinct patterns in the attribution scores across protein sequences, we performed a k-means clustering analysis. The optimal number of clusters was determined using the elbow method, which examines the relationship between the number of clusters (k) and the within-cluster sum of squares (inertia). For each value of k from 1 to min(10, n), where n is the number of sequences, we calculated the percentage decrease in inertia compared to $k - 1$. The optimal k was selected when adding additional clusters yielded diminishing returns, defined as the first k where the percentage decrease in inertia fell below 20%. Prior to clustering, the attribution scores were standardized using Z-score normalization to ensure equal weighting of features. The resulting clusters were visualized using PCA. This approach facilitated the identification of natural groupings in the attribution patterns while avoiding overfitting through excessive data partitioning.

DeepLift explainer analyses

This analysis used DeepLIFT[24] (deep learning important features), a method for computing feature importance scores. While DeepLIFT forms the basis for DeepSHAP, it is distinct from Shapley value calculations. DeepLIFT works by comparing neuron activations to reference activations and back-propagating the differences to input features. In the analyses shown in Figure 4, a sliding-window approach was implemented where each window underwent independent tokenization and attribution analysis, with adjacent windows sharing 16-residue overlapping regions (e.g., window 1: positions 0–31, window 2: positions 16–47, and so forth). This windowing approach enabled fine-grained attribution mapping across the sequence length while maintaining computational efficiency suitable for larger models. The overlapping nature of the windows provides redundant sampling at boundary regions, enhancing the robustness of the attribution signals. Contrasting results from TI-inclusive and TI-free methods showed a distinct position-dependant trend in TI-inclusive models.

Layer-wise transformer output PCA, t-SNE, and UMAP projections

The HELIX program registers output from the individual transformer layers. For data manipulation and numerical operations, we used NumPy.[79] Signal-processing tasks, particularly peak detection, were handled using SciPy[73] (scipy.signal). The Transformers library[66] from Hugging Face was instrumental for this work. Model weights were accessed and loaded using the huggingface_hub and safetensors libraries. For dimensionality reduction, we used Scikit-learn's[80] PCA, t-SNE,[81] and UMAP[82] from the umap-learn library.

We performed PCA separately on each layer to capture layer-wise embedding geometry, whereas t-SNE and UMAP were run on the aggregated final-layer representations to visualize the overall structure of the model's learned residue features (Figure 3). Data visualization was done in Matplotlib[74] and svgwrite[83] for scalar vector graphic generation. Additional utilities included the gc module for memory management, argparse for parsing command-line arguments, and collections for efficient data structures.

Influential motif identification from transformer attributions

We developed the DMMP software (see Data S3)[13] to identify influential motifs in the model and quantify their impacts on model decisions. This method analyzes hidden states from the model and calculates position- and residue-specific influence scores across all layers to identify motifs that strongly influence the model's decisions. We used a multi-step process to identify influential amino acids within protein sequences, leveraging the hidden state representations from the GPTNeoX-based[26] LA^4SR model. Our approach involved extracting hidden states for each input sequence, computing influence scores by calculating the Euclidean distance between the mean representation of each amino acid and the mean of all others, and normalizing these scores before using peak detection to identify local maxima.

For each layer's hidden states, we computed a position-wise influence score using the L2 norm of the difference between each position's representation and the mean representation of that layer: influence = ||h_i − μ||_2, where h_i is the hidden state at position i and μ is the mean hidden state for the layer. This operation is vectorized across all positions: position_influence = np.array([np.linalg.norm(state − np.mean(state, axis = 0), axis = 1) for a state in hidden_states]). The resulting scores were min-max normalized to a [0, 1] range, then across all layers to obtain a single influence score per position. To identify peaks in this influence profile, we applied the find_peaks function from scipy.signal with a height threshold set at the 95th percentile of all scores and a minimum peak distance of positions. Around each identified peak, we extracted a window of amino acids to form potential motifs. Patterns were extracted that exert a disproportionate influence on the model's decision-making process, revealing class-defining signatures. These peaks are considered the centers of influential motifs, extracted as subsequences of 3–6 residues centered on each peak. The motifs are then ranked by their average influence score, providing a prioritized list of potentially functionally or structurally important amino acid subsequences within the protein.

Flexible and strict influential motif discovery was done using DMMP. To identify biologically relevant sequence patterns learned by our model, we developed two complementary attribution-based motif discovery approaches: a flexible motif finder used for model interpretability (Figures 5 and 6) and a strict motif finder used for functional annotation. The flexible motif finder identifies variable-length patterns containing a mixture of conserved and degenerate positions (e.g., "XRXDX") by scanning attribution profiles derived from layer-wise hidden state influence. Using a sliding window and peak detection over averaged position-wise attribution scores, we extracted windows enriched for locally high influence. Motifs were defined as windows with at least two non-degenerate positions exceeding the 95th percentile in attribution. Overlapping motifs were merged to account for variable motif boundaries, and outputs were visualized in a heatmap across sequences.

The main function for the flexible motif-based analysis program was identify_influential_motifs() (identify_influential_motifs (hidden_states, sequence, window_size = n, percentile = n)). Influence scores were calculated for each position, where position_influence[layer, pos] = np.linalg.norm(pos_state − other_state). To minimize terminal sequence effects, we normalized positional influence scores (position_influence = (position_influence − position_influence.min())/(position_influence.max() − position_influence.min())). To extract motif-based influence scores, the average influence across layers was calculated (avg_influence = np.mean(position_influence, axis = 0). From average influence, peaks (peaks = find_peaks(avg_influence, height = threshold, distance = window_size) above a threshold (threshold = np.percentile(avg_influence, percentile) were returned with the following function: def print_influential_motifs (motifs, avg_influence, percentile, top_n = 10): print(f"\nTop top_n Influential Motifs:"); print("Motif | Start Position | Influence Score"); print("-" * 40); for motif, start, score in motifs[:top_n]: print(f"motif:5 | (start:15] | (score:.4f]").

In contrast, the strict motif finder identified short, fully conserved patterns by integrating three complementary attribution signals: self-attention diagonals, integrated gradients (IGs), and hidden state divergence. Self-attention diagonals reflect each token's intrinsic salience by measuring how much it attends to itself across all layers and heads. IGs quantify the contribution of each input position to the model's predicted token by integrating gradients from a zero-embedding baseline to the actual input. Hidden state divergence captures how distinct each token's representation is compared to the rest of the sequence, highlighting contextually unique residues. Each attribution channel was min-max normalized and combined into a composite score (0.4 × divergence + 0.3 × attention + 0.3 × IG) for every position. Motifs of 3–9 residues were extracted when the window's average composite score exceeded the 95th percentile and at least 75% of residues within it also exceeded that threshold. These strict, fully specified motifs were used for downstream mapping to PFAM domains and functional enrichment. The full implementation and reproducible scripts are provided in Data S3.[13]

Mapping model-derived motifs to PFAM domains

To biologically validate motifs identified by DMMP, we developed a scalable sequence scanning pipeline to map model-attributed

CellPress
OPEN ACCESS

motifs to known protein domains. High-influence motifs were aggregated from both bacterial and algal predictions (see Table S3). For each unique motif, we calculated a mean influence score across its appearances in the training data. To assess functional relevance, we searched these motifs against the full PFAM-A protein domain database using a regex-based sequence scanning approach. Each PFAM entry, composed of a domain-specific multiple sequence alignment and representative full-length sequences, was parsed from FASTA format. Using a compiled regular expression containing all model-attributed motifs, the pipeline scanned every sequence for motif occurrences in parallel across available CPU cores.

For each motif match, we recorded the corresponding PFAM accession and counted the number of times the motif occurred within sequences associated with that domain. This enabled us to construct a matrix of motif-PFAM associations, where high-frequency co-occurrence indicated a potential biological linkage. By merging these counts with the influence scores assigned by HELIX or DMMP, we prioritized motifs that were both salient to the model and recurrent in specific protein families.

Motif-PFAM association scoring, sorting, and filtering

To assess the biological plausibility of sequence motifs identified by our language model interpretability tools, we developed a post-processing pipeline that integrates model-derived salience with sequence-level recurrence. For each motif-PFAM pair (from ftp://ftp.ebi.ac.uk/pub/databases/PFAM/current_release/PFAM-A.fasta.gz) identified in the scanning step (see above), we calculated a simple composite score by multiplying the number of motif occurrences in PFAM-A.fasta, per PFAM (count), with its mean model-assigned influence score (mean_score): [weighted score = count $\times$ mean score].

This scoring scheme favors motif-domain associations that are both important to the model (i.e., consistently contribute to classification decisions) and frequently observed in nature (i.e., appear recurrently in PFAM-A sequences). This approach overcomes a key limitation of our earlier framework, in which each motif was assigned a single influence score regardless of its distribution across PFAM entries, resulting in many ties and limiting resolution during downstream prioritization. By incorporating recurrence, the new weighted score provides a more differentiated and biologically grounded ranking of motif-domain relationships, enabling clearer identification of functional associations and lineage-specific domain preferences.

The results of the motif-PFAM scanning step were stored as a large tab-separated file containing one row per motif-PFAM match, with columns for motif identity, PFAM accession, raw match count, and mean influence score. We appended the new weighted score as a fifth column and sorted the file in descending order of this value to prioritize high-confidence motif-domain pairs. This process was implemented as a streaming-safe Bash pipeline, ensuring compatibility with large input files (>1 GB).

This weighting scheme may introduce modest bias due to over-representation of well-characterized domains in the PFAM-A database, which is a beneficial trade-off because it increases resolution and robustness for motif ranking while still preserving the interpretive value of influence-based motif selection. Moreover, it allows for downstream enrichment analyses and visualization strategies that rely on motif-domain frequency relationships.

High-influence peptide motifs identified by our attribution analysis were subjected to organism-agnostic pathway enrichment as follows. First, each motif was scanned against the PFAM-A library (v.33.1) using HMMER3 to assign conserved protein domains. Domain-centric GO (dcGO; https://suPFAM.mrc-lmb.cam.ac.uk/SUPERFAMILY/cgi-bin/dcenrichment.cgi) annotations were then retrieved and used as input for Reactome (v.81)[28] pathway enrichment. Because Reactome curates conserved biochemical modules—such as ATP-binding P loops, kinase folds, and glycosyltransferase cores—pathway names (e.g., MAPK signaling, EGFR cascades, and cell-cycle checkpoints) may reflect human-centric nomenclature despite deriving from algal sequences. This cross-taxon, literature-driven approach leverages Reactome's ontology integration and species-agnostic data model to provide clear, context-rich interpretation of motif-based discoveries.

Containerized deployment of LA^{4}SR for scalable sequence analysis

To facilitate reproducible, high-throughput deployment of the LA^{4}SR framework across a variety of computational environments, we developed a comprehensive Singularity[84,85] container encapsulating all dependencies required for both model training and inference. The container supports diverse model architectures—including transformer-based (e.g., GPT-NeoX, Pythia, and Mistral) and state-space (e.g., Mamba) models—alongside all pre-processing, explainability, and visualization tools developed for this study. All software dependencies for our genomic analyses were encapsulated in a Singularity container built from an Ubuntu 20.04 base image on an AMD EPYC 7742 (2.25 GHz) host with cuda support for NVIDIA A100 80 GB GPUs. The user only needs a compiler (gcc/13.2.0), CUDA libraries (cuda/12.2.0), and Singularity[84,85] (singularity/4.2.0); the other 200 dependencies are included in the container.

All models presented in the study—including pre-trained and fine-tuned variants—are executable from within the container using lightweight wrapper scripts that support TI-inclusive and TI-free inference modes. GPU acceleration is fully supported and configured for A100, V100, and H100 NVIDIA GPUs in nodes on high-performance computing clusters. The container architecture ensures compatibility with both slurm-scheduled batch jobs and interactive shell environments and includes all tokenizer, configuration, and checkpoint files required for zero-setup operation. The LA^{4}SR container enables scalable, architecture-agnostic deployment of LLMs for biological sequence classification, democratizing access to high-performance deep-learning tools for microbial genomics. The container and associated resources are freely available for academic use (Data S5)[13] and will be released alongside the LA^{4}SR publication to facilitate transparent evaluation and adoption.

RESOURCE AVAILABILITY

Lead contact

Requests for further information and resources should be directed to and will be fulfilled by the lead contact, David R. Nelson (drn2@nyu.edu).

Materials availability

This study did not generate new unique reagents.

Data and code availability

- The original training and testing datasets, sequencing data, the evaluation and interpretability software programs, and the Singularity[84,85] container have been deposited at Zenodo: https://doi.org/10.5281/zenodo.13920000.[13] The Bioproject accession for the sequencing data generated in this study is NCBI: SAMN44618602.
- Additional files supporting this paper are available at Zenodo[13] as Data S1–S5. All training and inferencing scripts used in this study are available in Data S2 and S3. The Zenodo repository also contains an LA^{4}SR Singularity[84,85] container (Data S5).[13] LA^{4}SR models are hosted on huggingface.co.[76,77,86–92]

ACKNOWLEDGMENTS

We thank members of the Salehi-Ashtiani and Jean-Claude Twizere labs for discussions on the project. This research was supported by NYUAD Faculty Research Funds (AD060) and carried out on the High Performance Computing (HPC) resources at New York University Abu Dhabi. We especially thank the HPC management team and system administrators for maintaining a cutting-edge computer infrastructure that facilitates scalable machine-learning projects.

AUTHOR CONTRIBUTIONS

D.R.N. wrote the manuscript with input from the other authors and performed formal analyses. K.S.-A. and A.K.J. performed data curation and formal analyses. N.I. performed software testing. A.M. grew and prepared the *Chlamydomonas reinhardtii* CC-1883 cw15 NIT$^+$ mt-strain for sequencing. K.S.-A., D.R.N., and A.K.J. developed the conceptual framework of the project. K.S.-A. oversaw the completion of the work.

DECLARATION OF INTERESTS

The authors declare no competing interests.

DECLARATION OF GENERATIVE AI AND AI-ASSISTED TECHNOLOGIES IN THE WRITING PROCESS

During the preparation of this work the authors used Perplexity.ai,[93] ChatGPT 4o/o1/o3/o4/4.5,[94] and Claude Sonnet 3.5/3.7/4.0[95] to simplify language where possible without compromising content. After using this tool/service, the authors reviewed and edited the content as needed and take full responsibility for the content of the publication.

SUPPLEMENTAL INFORMATION

Supplemental information can be found online at https://doi.org/10.1016/j.patter.2025.101373.

Received: February 14, 2025
Revised: May 30, 2025
Accepted: August 28, 2025
Published: September 24, 2025

REFERENCES

1. Alberts, B., Johnson, A., Lewis, J., Morgan, D., Raff, M., Roberts, K., and Walter, P. (2014). Molecular Biology of the Cell, 6 Edn (Garland Science/W. W. Norton & Company).
2. Dill, K.A., and MacCallum, J.L. (2012). The protein-folding problem, 50 years on. Science *338*, 1042–1046. https://doi.org/10.1126/science.1219021.
3. Puigbo, P., Wolf, Y.I., and Koonin, E.V. (2010). The tree and net components of prokaryote evolution. Genome Biol Evol *2*, 745–756. https://doi.org/10.1093/gbe/evq062.
4. Battistuzzi, F.U., Feijao, A., and Hedges, S.B. (2004). A genomic timescale of prokaryote evolution: insights into the origin of methanogenesis, phototrophy, and the colonization of land. BMC Evol. Biol. *4*, 44. https://doi.org/10.1186/1471-2148-4-44.
5. Margulis, L., Dolan, M.F., and Guerrero, R. (2000). The chimeric eukaryote: origin of the nucleus from the karyomastigont in amitochondriate protists. Proc. Natl. Acad. Sci. USA *97*, 6954–6959. https://doi.org/10.1073/pnas.97.13.6954.
6. Stegemann, S., Hartmann, S., Ruf, S., and Bock, R. (2003). High-frequency gene transfer from the chloroplast genome to the nucleus. Proc. Natl. Acad. Sci. USA *100*, 8828–8833. https://doi.org/10.1073/pnas.1430924100.
7. Hedges, S.B., Blair, J.E., Venturi, M.L., and Shoe, J.L. (2004). A molecular timescale of eukaryote evolution and the rise of complex multicellular life. BMC Evol. Biol. *4*, 2. https://doi.org/10.1186/1471-2148-4-2.
8. Zimmer, A., Lang, D., Richardt, S., Frank, W., Reski, R., and Rensing, S.A. (2007). Dating the early evolution of plants: detection and molecular clock analyses of orthologs. Mol. Genet. Genomics. *278*, 393–402. https://doi.org/10.1007/s00438-007-0257-6.
9. Parfrey, L.W., Lahr, D.J.G., Knoll, A.H., and Katz, L.A. (2011). Estimating the timing of early eukaryotic diversification with multigene molecular clocks. Proc. Natl. Acad. Sci. USA *108*, 13624–13629. https://doi.org/10.1073/pnas.1110633108.
10. Barbrook, A.C., Santucci, N., Plenderleith, L.J., Hiller, R.G., and Howe, C.J. (2004). Plastid-derived single gene minicircles of the dinoflagellate *Ceratium horridum* are localized in the nucleus. Mol. Biol. Evol. *21*, 2310–2318. https://doi.org/10.1093/molbev/msh244.
11. Buchfink, B., Xie, C., and Huson, D.H. (2015). Fast and sensitive protein alignment using DIAMOND. Nat. Methods *12*, 59–60. https://doi.org/10.1038/nmeth.3176.
12. Reimand, J., Wagih, O., and Bader, G.D. (2015). Evolutionary constraint and disease associations of post-translational modification sites in human genomes. PLoS Genet. *11*, e1004919. https://doi.org/10.1371/journal.pgen.1004919.
13. Nelson, D.R., and Salehi-Ashtiani, K. (2025). Supplemental Datasets for: 'Pan-Microalgal Dark Proteome Mapping via Interpretable Deep Learning and Synthetic Chimeras'. Zenodo, https://doi.org/10.5281/zenodo.15559257.
14. Camacho, C., Coulouris, G., Avagyan, V., Ma, N., Papadopoulos, J., Bealer, K., and Madden, T.L. (2009). BLAST$^+$: architecture and applications. BMC Bioinf. *10*, 421. https://doi.org/10.1186/1471-2105-10-421.
15. Na, C.-W., Paek, E., Kim, M., Kim, J.-H., Kim, S., Kim, H., Lee, H., Kim, S.-Y., Kim, K.-J., and Kim, S. (2018). Comprehensive analysis of human protein N-termini enables assessment of alternative translation initiation, proteolytic processing and N-terminal modifications. Nat. Commun. *9*, 1026. https://doi.org/10.1038/s41467-018-03254-5.
16. Gao, L., Biderman, S., Black, S., Golding, L., Hoppe, T., Foster, C., Phang, J., He, H., Thite, A., Nabeshima, N., et al. (2020). The Pile: an 800GB dataset of diverse text for language modeling. Preprint at arXiv. https://doi.org/10.48550/arXiv.2101.00027.
17. Curtis, B.A., Tanifuji, G., Burki, F., Gruber, A., Irimia, M., Maruyama, S., Arias, M.C., Ball, S.G., Gile, G.H., Hirakawa, Y., et al. (2012). Algal genomes reveal evolutionary mosaicism and the fate of nucleomorphs. Nature *492*, 59–65. https://doi.org/10.1038/nature11681.
18. Leung, S.K., and Wong, J.T.Y. (2009). The replication of plastid minicircles involves rolling circle intermediates. Nucleic Acids Res. *37*, 1991–2002. https://doi.org/10.1093/nar/gkp063.
19. Karas, B.J., Diner, R.E., Lefebvre, S.C., McQuaid, J., Phillips, A.P.R., Noddings, C.M., Brunson, J.K., Valas, R.E., Deerinck, T.J., Jablanovic, J., et al. (2015). Designer diatom episomes delivered by bacterial conjugation. Nat. Commun. *6*, 6925. https://doi.org/10.1038/ncomms7925.

CellPress
OPEN ACCESS

20. Diner, R.E., Noddings, C.M., Lian, N.C., Kang, A.K., McQuaid, J.B., Jablanovic, J., Espinoza, J.L., Nguyen, N.A., Anzelmatti, M.A., Jansson, J., et al. (2016). Refinement of the diatom episome maintenance sequence and improved protocols for episome delivery. Front. Bioeng. Biotechnol. *4*, 65. https://doi.org/10.3389/fbioe.2016.00065.

21. Sundararajan, M., Taly, A., and Yan, Q. (2017). Axiomatic attribution for deep networks. Preprint at arXiv. https://doi.org/10.48550/arXiv.1703.01365.

22. Belrose, N., Ostrovsky, I., McKinney, L., Furman, Z., Smith, L., Halawi, D., Biderman, S., and Steinhardt, J. (2023). Eliciting latent predictions from transformers with the Tuned Lens. Preprint at arXiv. https://doi.org/10.48550/arXiv.2303.08112.

23. Kokhlikyan, N., Miglani, V., Martin, M., Wang, E., Alsallakh, B., Reynolds, J., Melnikov, A., Kliushkina, N., Araya, C., Yan, S., and Reblitz-Richardson, O. (2020). Captum: A unified and generic model interpretability library for PyTorch. Preprint at arXiv. https://doi.org/10.48550/arXiv.2009.07896.

24. Shrikumar, A., Greenside, P., and Kundaje, A. (2017). Learning important features through propagating activation differences. Preprint at arXiv. https://doi.org/10.48550/arXiv.1704.02685.

25. Contreras, J., Winterfeld, A., Popp, J., and Bocklitz, T. (2024). Spectral zones-based SHAP/LIME: enhancing interpretability in spectral deep learning models through grouped feature analysis. Anal. Chem. *96*, 15588–15597. https://doi.org/10.1021/acs.analchem.4c02329.

26. Andonian, A., Anthony, Q., Biderman, S., Black, S., Gali, P., Gao, L., Hallahan, E., Levy-Kramer, J., Leahy, C., Nestler, L., et al. (2023). GPT-NeoX: Large Scale Autoregressive Language Modeling in PyTorch. Zenodo, https://doi.org/10.5281/zenodo.5879544.

27. Bao, C., Lu, C., Lin, J., Gough, J., and Fang, H. (2023). The dcGO domain-centric ontology database in 2023: new website and extended annotations for protein structural domains. J. Mol. Biol. *435*, 168093. https://doi.org/10.1016/j.jmb.2023.168093.

28. Jassal, B., Matthews, L., Viteri, G., Gong, C., Lorente, P., Fabregat, A., Sidiropoulos, K., Cook, J., Gillespie, M., Haw, R., et al. (2024). The Reactome pathway knowledgebase 2024. Nucleic Acids Res. *52*, D963–D971. https://doi.org/10.1093/nar/gkad1029.

29. Milacic, M., Beavers, D., Conley, P., Gong, C., Gillespie, M., Griss, J., Haw, R., Jassal, B., Matthews, L., May, B., et al. (2024). The Reactome Pathway Knowledgebase 2024. Nucleic Acids Res. *52*, D672–D678. https://doi.org/10.1093/nar/gkad1025.

30. Martinez-Yamout, M.A., Nasir, I., Shnitkind, S., Ellis, J.P., Berlow, R.B., Kroon, G., Deniz, A.A., Dyson, H.J., and Wright, P.E. (2023). Glutamine-rich regions of the disordered CREB transactivation domain mediate dynamic intra- and intermolecular interactions. Proc. Natl. Acad. Sci. USA *120*, e2313835120. https://doi.org/10.1073/pnas.2313835120.

31. Michelitsch, M.D., and Weissman, J.S. (2000). A census of glutamine/asparagine-rich regions: implications for their conserved function and the prediction of novel prions. Proc. Natl. Acad. Sci. USA *97*, 11910–11915. https://doi.org/10.1073/pnas.97.22.11910.

32. Ruiz, D.M., Turowski, V.R., and Murakami, M.T. (2016). Effects of the linker region on the structure and function of modular GH5 cellulases. Sci. Rep. *6*, 28504. https://doi.org/10.1038/srep28504.

33. Liu, Y., Ott, M., Goyal, N., Du, J., Joshi, M., Chen, D., Levy, O., Lewis, M., Zettlemoyer, L., and Stoyanov, V. (2019). RoBERTa: a robustly optimized BERT pretraining approach. Preprint at arXiv. https://doi.org/10.48550/arXiv.1907.11692.

34. Sanh V, D.L., Chaumond J, Wolf T (2019). DistilRoBERTa-base: A distilled version of RoBERTa-base. Hugging Face. https://huggingface.co/distilbert/distilroberta-base.

35. Sanh, V., Debut, L., Chaumond, J., and Wolf, T. (2019). DistilBERT, a distilled version of BERT: smaller, faster, cheaper and lighter. Preprint at arXiv. https://doi.org/10.48550/arXiv.1910.01108.

36. Paszke, A., Gross, S., Massa, F., Lerer, A., Bradbury, J., Chanan, G., Killeen, T., Lin, Z., Gimelshein, N., Antiga, L., et al. (2019). PyTorch: An imperative style, high-performance deep learning library. Preprint at arXiv. https://doi.org/10.48550/arXiv.1912.01703.

37. Fu, L., Jung, Y., Tian, C., Ferreira, R.B., Cheng, R., He, F., Yang, J., and Carroll, K.S. (2023). Nucleophilic covalent ligand discovery for the cysteine redoxome. Nat. Chem. Biol. *19*, 1309–1319. https://doi.org/10.1038/s41589-023-01330-5.

38. Held, J.M. (2020). Redox systems biology: harnessing the sentinels of the cysteine redoxome. Antioxid. Redox Signal. *32*, 659–676. https://doi.org/10.1089/ars.2019.7725.

39. Schalka, S., Silva, M.S., Lopes, L.F., de Freitas, L.M., and Baptista, M.S. (2022). The skin redoxome. J. Eur. Acad. Dermatol. Venereol. *36*, 181–195. https://doi.org/10.1111/jdv.17780.

40. Jacobs, P.J., Hart, D.W., Merchant, H.N., Voigt, C., and Bennett, N.C. (2023). The evolution and ecology of oxidative and antioxidant status: a comparative approach in African mole-rats. Antioxidants *12*, 1486. https://doi.org/10.3390/antiox12081486.

41. Harada, M., Akiyama, A., Furukawa, R., Yokobori, S.I., Tajika, E., and Yamagishi, A. (2021). Evolution of superoxide dismutases and catalases in cyanobacteria: occurrence of the antioxidant enzyme genes before the rise of atmospheric oxygen. J. Mol. Evol. *89*, 527–543. https://doi.org/10.1007/s00239-021-10021-5.

42. Acquisti, C., Kleffe, J., and Collins, S. (2007). Oxygen content of transmembrane proteins over macroevolutionary time scales. Nature *445*, 47–52. https://doi.org/10.1038/nature05450.

43. Ness, R.W., Morgan, A.D., Vasanthakrishnan, R.B., Colegrave, N., and Keightley, P.D. (2015). Extensive *de novo* mutation rate variation between individuals and across the genome of *Chlamydomonas reinhardtii*. Genome Res. *25*, 1739–1749. https://doi.org/10.1101/gr.191494.115.

44. Morgan, A.D., Ness, R.W., Keightley, P.D., and Colegrave, N. (2014). Spontaneous mutation accumulation in multiple strains of the green alga, *Chlamydomonas reinhardtii*. Evolution *68*, 2589–2602. https://doi.org/10.1111/evo.12448.

45. Ness, R.W., Morgan, A.D., Colegrave, N., and Keightley, P.D. (2012). Estimate of the spontaneous mutation rate in *Chlamydomonas reinhardtii*. Genetics *192*, 1447–1454. https://doi.org/10.1534/genetics.112.145078.

46. Nelson, D.R., Hazzouri, K.M., Lauersen, K.J., Jaiswal, A., Chaiboonchoe, A., Mystikou, A., Fu, W., Daakour, S., Dohai, B., Alzahmi, A., et al. (2021). Large-scale genome sequencing reveals the driving forces of viruses in microalgal evolution. Cell Host Microbe *29*, 250–266.e8. https://doi.org/10.1016/j.chom.2020.12.005.

47. Gilson, P.R., Su, V., Slamovits, C.H., Reith, M.E., Keeling, P.J., and McFadden, G.I. (2006). Complete nucleotide sequence of the chlorarachniophyte nucleomorph: nature's smallest nucleus. Proc. Natl. Acad. Sci. USA *103*, 9566–9571. https://doi.org/10.1073/pnas.0600707103.

48. GreenGenomicsLab (2025). GreenGenomicsLab: Machine Learning Models and Datasets for Genomic Research. Hugging Face, https://huggingface.co/GreenGenomicsLab.

49. Davis, C.J. (2010). The spatial coding model of visual word identification. Psychol. Rev. *117*, 713–758. https://doi.org/10.1037/a0019738.

50. Graves, A., Mohamed, A.R., and Hinton, G. (2013). Speech recognition with deep recurrent neural networks. In 2013 IEEE International Conference on Acoustics, Speech and Signal Processing, pp. 6645–6649. https://doi.org/10.1109/ICASSP.2013.6638947.

51. Dandurand, F., Grainger, J., and Duñabeitia, J.A. (2011). On coding non-contiguous letter combinations. Front. Psychol. *2*, 00136. https://doi.org/10.3389/fpsyg.2011.00136.

52. McClelland, J.L., and Rumelhart, D.E. (1981). An interactive activation model of context effects in letter perception: I. An account of basic findings. Psychol. Rev. *88*, 375–407. https://doi.org/10.1037/0033-295X.88.5.375.

53. Anusha, Zhang, Z., Zhang, Z., Li, J., Zuo, H., and Mao, C. (2024). AlphaFold 3 - aided design of DNA motifs to assemble into triangles. J. Am. Chem. Soc. *146*, 25422–25425. https://doi.org/10.1021/jacs.4c08387.

54. Ribeiro, M.T., Singh, S., and Guestrin, C. (2016). Why Should I Trust You?": explaining the predictions of any classifier. In Proceedings of the 22nd ACM SIGKDD International Conference on Knowledge Discovery and Data Mining (Association for Computing Machinery), pp. 1135–1144.
55. Hu, E.J., Shen, Y., Wallis, P., Allen-Zhu, Z., Li, Y., Wang, S., Wang, L., and Chen, W. (2021). LoRA: low-rank adaptation of large language models. Preprint at arXiv. https://doi.org/10.48550/arXiv.2106.09685.
56. Vaswani, A., Shazeer, N., Parmar, N., Uszkoreit, J., Jones, L., Gomez, A. N., Kaiser, Ł., and Polosukhin, I. (2017). Attention is all you need. In Proceedings of the 31st International Conference on Neural Information Processing Systems (Curran Associates Inc.), pp. 6000–6010.
57. Usman, J., Abba, S.I., Abdu, F.J., Yogarathinam, L.T., Usman, A.G., Lawal, D., Salhi, B., and Aljundi, I.H. (2024). Enhanced desalination with polyamide thin-film membranes using ensemble ML chemometric methods and SHAP analysis. RSC Adv. *14*, 31259–31273. https://doi.org/10.1039/d4ra06078d.
58. Gu, A., and Dao, T. (2023). Mamba: linear-time sequence modeling with selective state spaces. Preprint at arXiv. https://doi.org/10.48550/arXiv.2312.00752.
59. Dao, T., and Gu, A. (2024). Transformers are SSMs: generalized models and efficient algorithms through structured state space duality. Preprint at arXiv. https://doi.org/10.48550/arXiv.2405.21060.
60. Jiang, A.Q., Sablayrolles, A., Mensch, A., Bamford, C., Singh Chaplot, D., de las Casas, D., Bressand, F., Lengyel, G., Lample, G., Saulnier, L., et al. (2023). Mistral 7B. Preprint at arXiv. https://doi.org/10.48550/arXiv.2310.06825.
61. Karpathy, A. (2023). nanoGPT: A minimal, fast repository for training/fine-tuning medium-sized GPTs. Version 1.0. GitHub, https://github.com/karpathy/nanoGPT.
62. Xue, L., Barua, A., Constant, N., Al-Rfou, R., Narang, S., Kale, M., Roberts, A., and Raffel, C. (2022). ByT5: Towards a token-free future with pre-trained byte-to-byte models. Trans. Assoc. Comput. Linguist. *10*, 291–306. https://doi.org/10.1162/tacl_a_00461.
63. Wang, W., Wei, F., Dong, L., Bao, H., Yang, N., and Zhou, M. (2020). MiniLM: deep self-attention distillation for task-agnostic compression of pre-trained transformers. Preprint at arXiv. https://doi.org/10.48550/arXiv.2002.10957.
64. Workshop, B., Le Scao, T., Fan, A., Akiki, C., Pavlick, E., Ilić, S., Hesslow, D., Castagné, R., Sasha Luccioni, A., Yvon, F., et al. (2022). BLOOM: A 176B-parameter open-access multilingual language model. Preprint at arXiv. https://doi.org/10.48550/arXiv.2211.05100.
65. Biderman, S., Schoelkopf, H., Anthony, Q., Bradley, H., O'Brien, K., Hallahan, E., Aflah Khan, M., Purohit, S., Sai Prashanth, U., Raff, E., et al. (2023). Pythia: a suite for analyzing large language models across training and scaling. Preprint at arXiv. https://doi.org/10.48550/arXiv.2304.01373.
66. Transformers v3.0.2: AutoModels documentation. (2020). Hugging Face, https://huggingface.co/transformers/v3.0.2/model_doc/auto.html.
67. NVIDIA: World Leader in Artificial Intelligence Computing. (2025). NVIDIA Corporation, https://www.nvidia.com/en-us/.
68. Korf, I. (2004). Gene finding in novel genomes. BMC Bioinf. *5*, 59. https://doi.org/10.1186/1471-2105-5-59.
69. Shirish Keskar, N., Mudigere, D., Nocedal, J., Smelyanskiy, M., and Tang, P.T.P. (2016). On large-batch training for deep learning: generalization gap and sharp minima. Preprint at arXiv. https://doi.org/10.48550/arXiv.1609.04836.
70. Nelson, D.R., Mystikou, A., Jaiswal, A., Rad-Menendez, C., Preston, M.J., De Boever, F., El Assal, D.C., Daakour, S., Lomas, M.W., Twizere, J.C., et al. (2024). Macroalgal deep genomics illuminate multiple paths to aquatic, photosynthetic multicellularity. Mol. Plant *17*, 747–771. https://doi.org/10.1016/j.molp.2024.03.011.
71. Cock, P.J.A., Antao, T., Chang, J.T., Chapman, B.A., Cox, C.J., Dalke, A., Friedberg, I., Hamelryck, T., Kauff, F., Wilczynski, B., and de Hoon, M.J.L. (2009). Biopython: freely available Python tools for computational molecular biology and bioinformatics. Bioinformatics *25*, 1422–1423. https://doi.org/10.1093/bioinformatics/btp163.
72. McKinney, W. (2010). Data structures for statistical computing in Python, S. van der Walt and J. Millman, eds., pp. 56–61.
73. Virtanen, P., Gommers, R., Oliphant, T.E., Haberland, M., Reddy, T., Cournapeau, D., Burovski, E., Peterson, P., Weckesser, W., Bright, J., et al. (2020). SciPy 1.0: fundamental algorithms for scientific computing in Python. Nat. Methods *17*, 261–272. https://doi.org/10.1038/s41592-019-0686-2.
74. Hunter, J.D. (2007). Matplotlib: a 2D graphics environment. Comput. Sci. Eng. *9*, 90–95. https://doi.org/10.1109/MCSE.2007.55.
75. Waskom, M. (2021). Seaborn: statistical data visualization. J. Open Source Softw. *6*, 3021. https://doi.org/10.21105/joss.03021.
76. GreenGenomicsLab. (2025). sentence-transformers-all-distilroberta-v1-dualityFT50000. Hugging Face, https://doi.org/10.57967/hf/6287.
77. GreenGenomicsLab. (2025). LA4SR-GPT2-ByT5_TI-free-30000. Hugging Face, https://doi.org/10.57967/hf/6282.
78. Shapley, L.S. (1988). A value for n-person games. In The Shapley value: essays in honor of Lloyd, S. Shapley and A.E. Roth, eds. (Cambridge University Press), pp. 31–40. https://doi.org/10.1017/CBO9780511528446.003.
79. Harris, C.R., Millman, K.J., van der Walt, S.J., Gommers, R., Virtanen, P., Cournapeau, D., Wieser, E., Taylor, J., Berg, S., Smith, N.J., et al. (2020). Array programming with NumPy. Nature *585*, 357–362. https://doi.org/10.1038/s41586-020-2649-2.
80. Pedregosa, F., Varoquaux, G., Gramfort, A., Michel, V., Thirion, B., Grisel, O., Blondel, M., Prettenhofer, P., Weiss, R., Dubourg, V., et al. (2011). Scikit-learn: Machine Learning in Python. J. Mach. Learn. Res. *12*, 2825–2830. https://scikit-learn.org/stable/.
81. Hinton, G., and Roweis, S. (2002). Stochastic neighbor embedding. In Proceedings of the 15th International Conference on Neural Information Processing Systems (MIT Press).
82. McInnes, L., Healy, J., and Melville, J. (2018). UMAP: uniform manifold approximation and projection for dimension reduction. Preprint at arXiv. https://doi.org/10.48550/arXiv.1802.03426.
83. Moitzi, M. (2022). svgwrite: A Python library to create SVG drawings (v.1.4.3). https://pypi.org/project/svgwrite/.
84. SyLabs (2025). Singularity. https://sylabs.io/singularity/.
85. Kurtzer, G.M., Sochat, V., and Bauer, M.W. (2017). Singularity: scientific containers for mobility of compute. PLoS One *12*, e0177459. https://doi.org/10.1371/journal.pone.0177459.
86. GreenGenomicsLab. (2025). LA4SR-Mamba-2.8b-QLORA-ft. Hugging Face, https://doi.org/10.57967/hf/6278.
87. GreenGenomicsLab. (2025). LA4SR-92.3-Pythia-70m-b-chk50000. Hugging Face, https://doi.org/10.57967/hf/6279.
88. GreenGenomicsLab. (2025). LA4SR-ELECTRA-FT530000. Hugging Face, https://doi.org/10.57967/hf/6280.
89. GreenGenomicsLab. (2025). gpt-neo125-ALMGA-FL. Hugging Face, https://doi.org/10.57967/hf/6281.
90. GreenGenomicsLab. (2025). Mamba-370m-88F1-45000. Hugging Face, https://doi.org/10.57967/hf/6283.
91. GreenGenomicsLab. (2025). LA4SR-Mistral7B-1000. Hugging Face, https://doi.org/10.57967/hf/6284.
92. GreenGenomicsLab. (2025). LA4SR-Pythia-12B-23000. Hugging Face, https://doi.org/10.57967/hf/6285.
93. Perplexity AI. (2025). Perplexity: AI-powered answer engine. https://www.perplexity.ai/.
94. OpenAI (2025). OpenAI: Advancing artificial general intelligence for the benefit of humanity. https://openai.com/.
95. Anthropic (2025). Claude 3.7 Sonnet: hybrid reasoning model. https://www.anthropic.com/news/claude-3-7-sonnet.

Patterns, Volume *6*

Supplemental information

Pan-microalgal dark proteome mapping via interpretable deep learning and synthetic chimeras

David R. Nelson, Ashish Kumar Jaiswal, Noha Samir Ismail, Alexandra Mystikou, and Kourosh Salehi-Ashtiani

Supplemental information for: "*Pan-microalgal dark proteome mapping via interpretable deep learning and synthetic chimeras*"

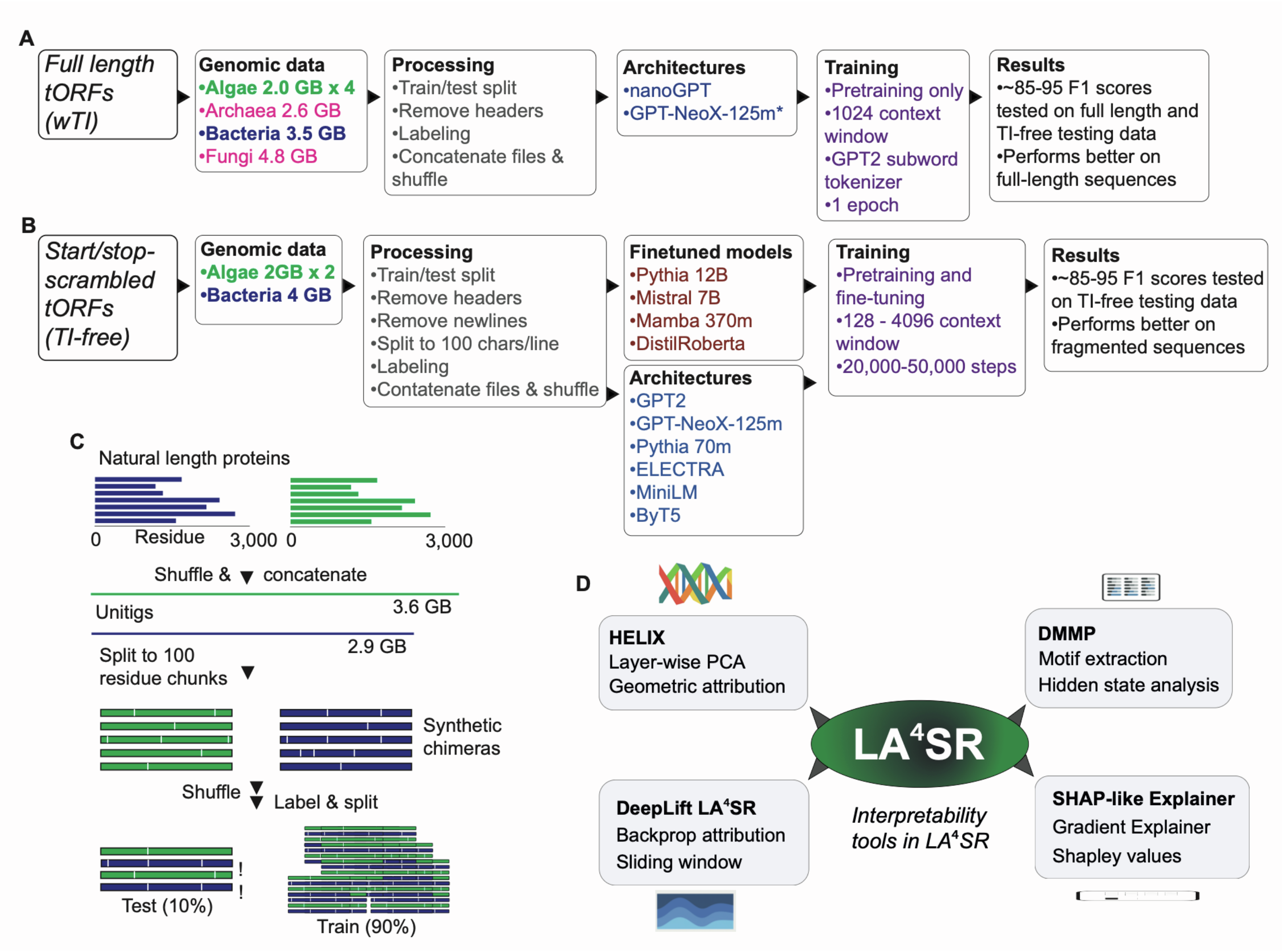

Figure S1. Schematic diagram of LM approaches for DNN embedding and representation of microbial genome-based amino acid sequence data

(A) Integrated, TI-inclusive approach. The TI-inclusive training regime used full-length protein sequences. The algaGPTnano and GPT-NeoX models were trained with a diverse representation of contaminant species' sequences (i.e, full-length proteins, full-spectrum (bacterial, fungal, archaeal), see Data S1). We used all available nonredundant microalgal genomic sequences passing a contamination filter, equaling about 2 gigabytes in total (Table S1).

(B) Core, TI-free approach. This approach had a directive to resolve intermediate regions regardless of TI. All input sequences per class were concatenated to a single line, then divided to lines of 100 residues each and shuffled. Resultant models exhibited robust performance with multiple adaptability and accessibility features, including small memory overhead and strong generalization capacity on incomplete sequences. The training data and scripts are in Data S1 and Data S2.

(C) Illustration of the TI-free set preparation.

(D) Interpretability tools for protein language models (PLMs). These models were developed to decipher the influence of amino acid patterns in the model's decision-making process at different scales.

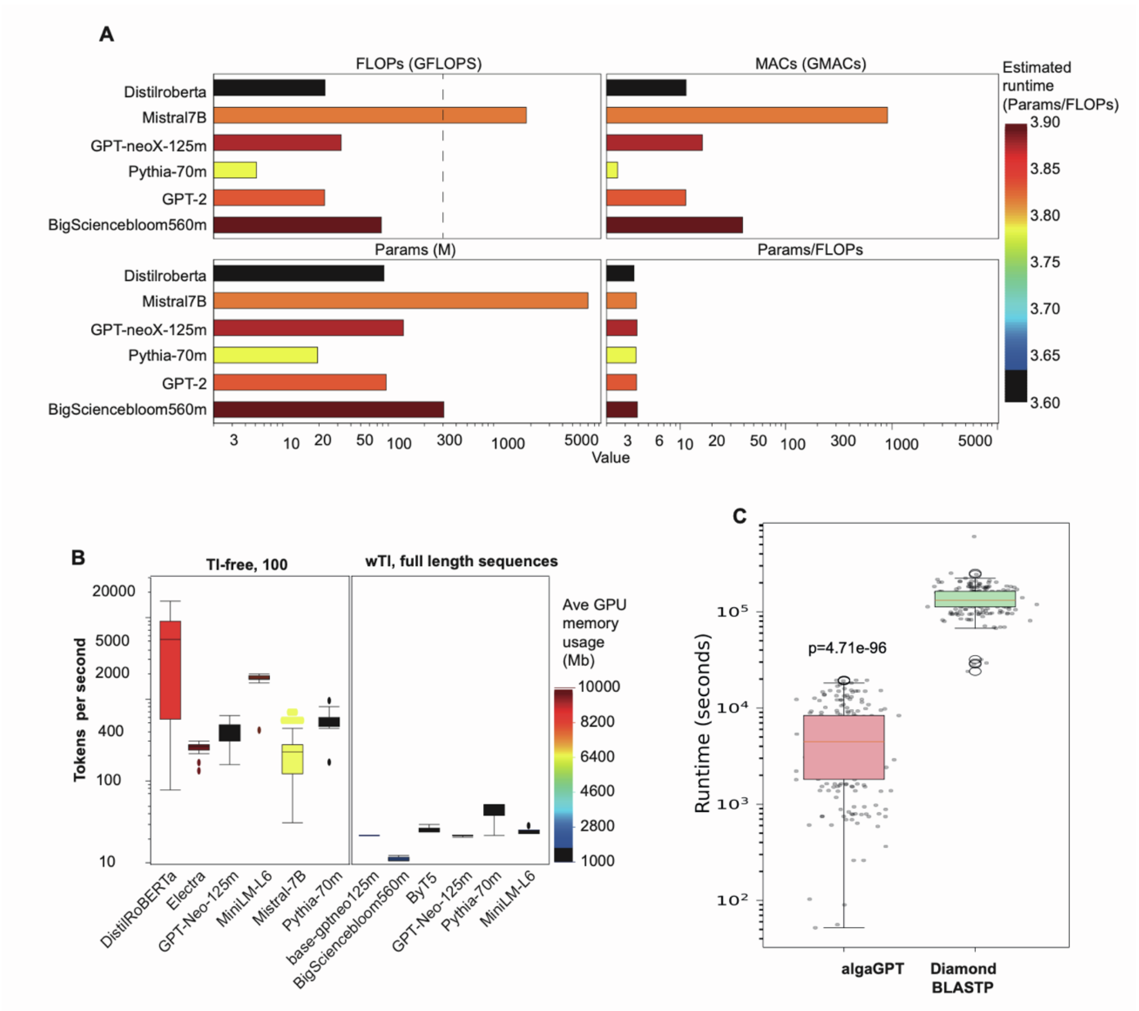

Figure S2. Technical performance evaluation of representative open-source LM model architectures used in LA^{4}SR

(A) Comparison of FLOPS, MACs, and parameters of representative LA^{4}SR models.
(B) The wTI and TI-free approaches are compared in terms of tokens per second and average GPU usage during inference of either TI-free (100 amino acids per query) and wTI (i.e., full-length sequences, average length = 197.76). We developed a custom evaluation pipeline that could accommodate a range of models. Results from compatible architectures are shown.
(C) Runtimes estimates of classification models. The algaGPT model runs 82.9x faster, on average, than Diamond BLASTP[S1], which has been shown to have up to 20,000 the speed of BLASTX (also see Table S1).

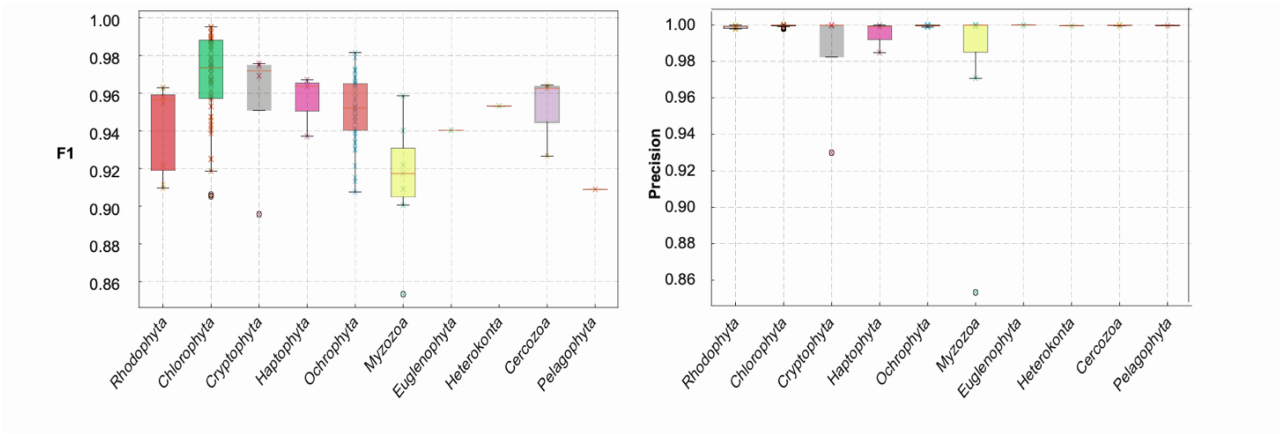

Figure S3. Microalgal lineage-stratified sensitivity analysis of LA^{4}SR classification performance.

Per-class F_1 and precision metrics were computed using the TI-free LA^{4}SR 13m model on full-length hold-out in silico proteomes. Boxplots depict the median (horizontal line), interquartile range (box), whiskers extending to 1.5× the interquartile range, and individual lineage scores as overlaid points. Despite uneven representation (e.g., very small clades or genera), median precision remains ≥ 0.98 across all groupings, confirming robust generalizability. F_1 score distributions aggregated by phylum across 10 microalgal lineages. Variation reflects differential sample representation and evolutionary distance; enforcing equal-size stratified sampling severely degraded performance in all phyla.

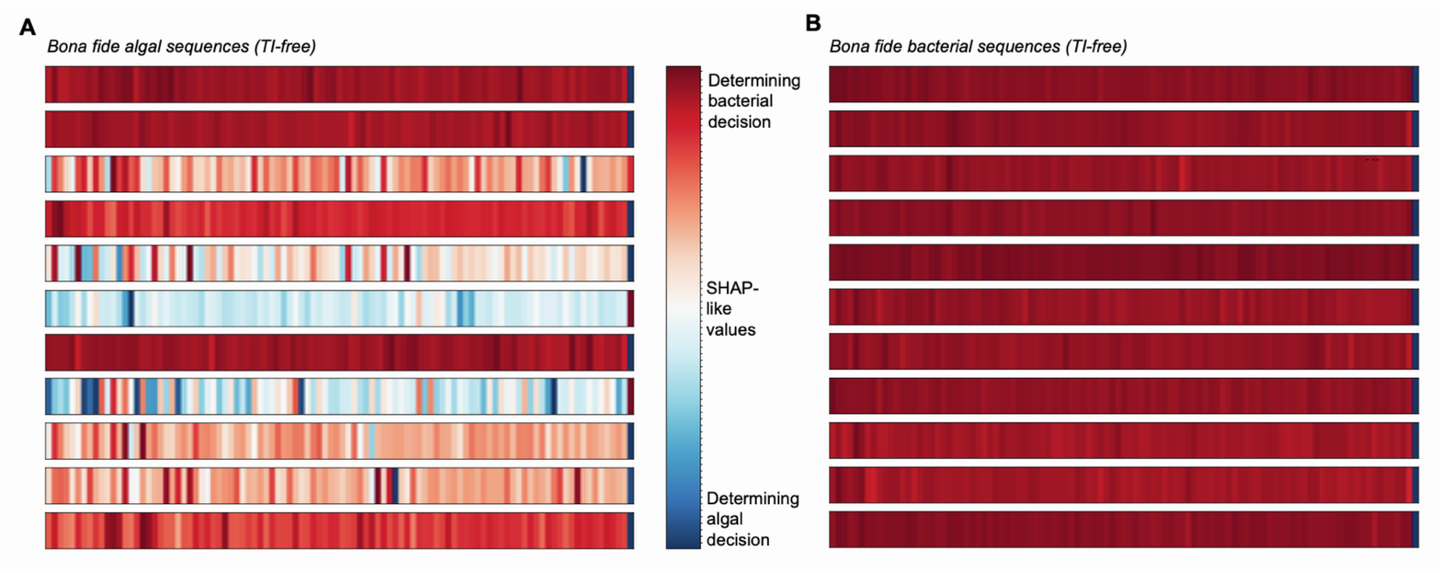

Figure S4. Feature attribution in bacterial and algal protein classification in a bi-directional BERT (distilRoBERTa)-based model.

(A) Heatmaps showing SHAP-like influence values for individual amino acids in TI-free algal protein sequences in a LA⁴SR -distilRoBERTa post-trained model. Each row represents a distinct protein sequence from the TI-free training set with amino acids displayed along the x-axis. Color intensity indicates the magnitude and direction of each amino acid's contribution to classification, with red signifying bacterial and blue algal prediction.

(B) Heatmaps showing SHAP-like influence values for individual amino acids in TI-free bacterial protein sequences in a LA⁴SR -distilRoBERTa post-trained model, similarly to (A).

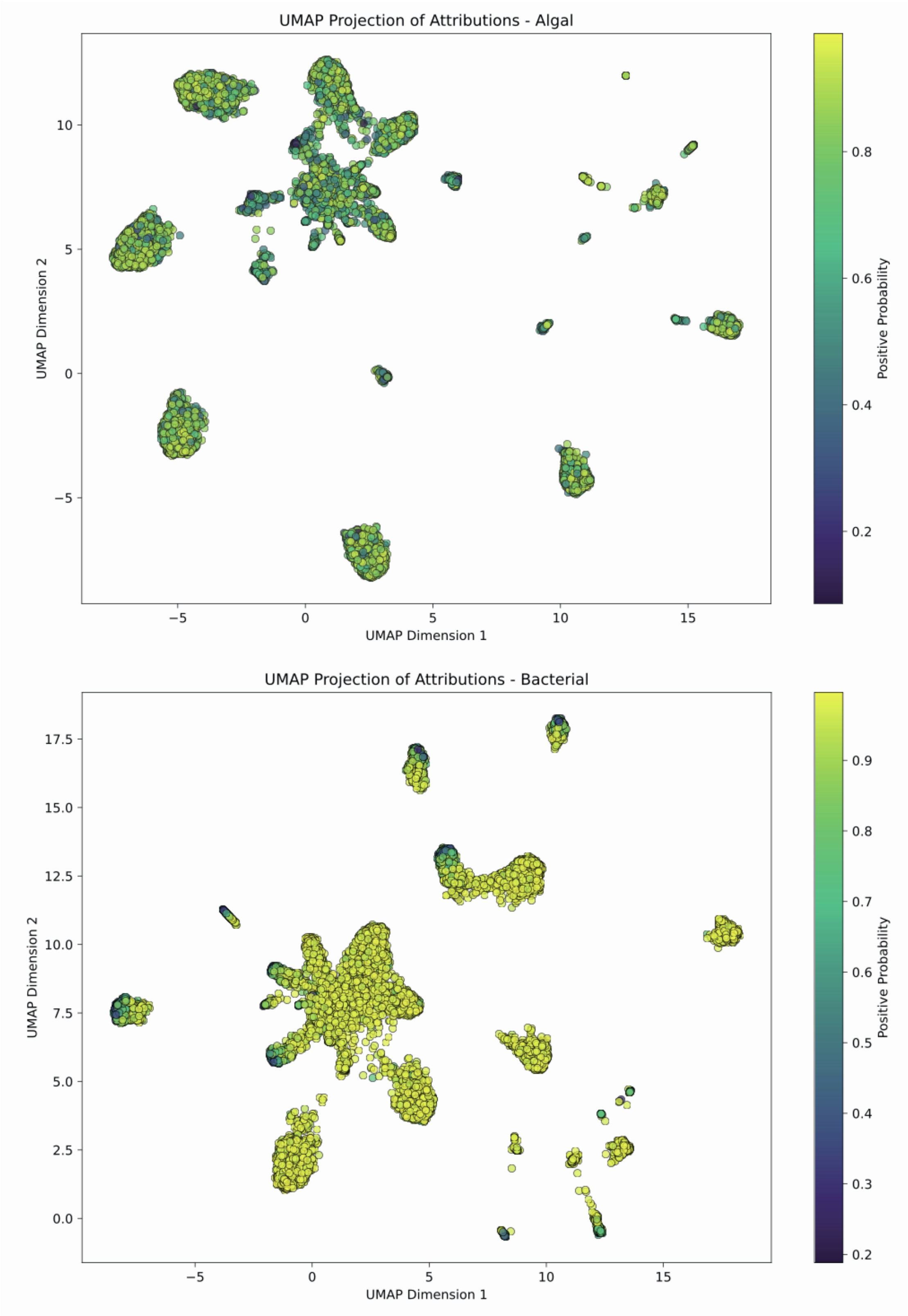

Figure S5. UMAP on attribution scores from 20,000 algal and bacterial sequences from LA^{4}SR model transformer outputs

A comparison of bacterial and algal inputs was analyzed with the ALMGA TI-inclusive LA^{4}SR model (Data S5). The tight clustering from UMAP reveals approximately ten large clusters of algal sequences, corresponding to the ten major algal phyla sampled in the training data, with a relatively low positive probability of being identified as a contaminant.

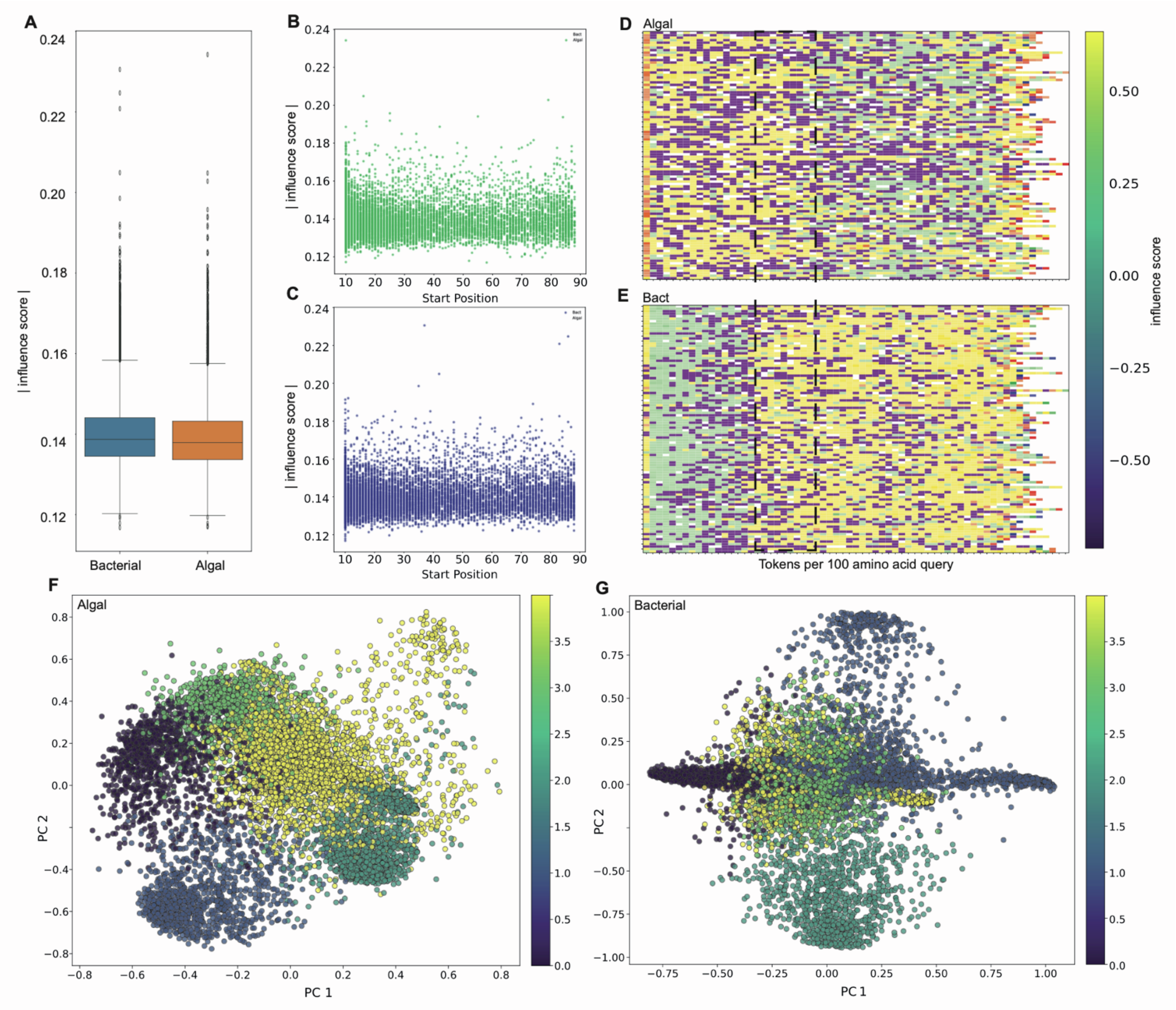

Figure S6. Captum-based analysis of algal and bacterial per-residue attribution scores

(A) Distributions of absolute influence scores in algal and bacterial sequences in a Pythia 70m-based LA^{4}SR model.

(B-C) Distributions of absolute influence scores per position in algal and bacterial sequences, showing a relatively even distribution of influence scores nearly independent of position.

(D-E) Heatmaps showing influence scores from sequences of tokens per 100 amino acid queries for algal (top) and bacterial (bottom) samples. The x-axis represents the position in the sequence, and the y-axis represents individual samples. The color scale ranges from -0.50 (blue: negative influence scores) to 0.50 (yellow: positive influence scores). The contrasting heatmaps give insight into the LA^{4}SR prediction process. The sequences around the 30 amino acid mark appear highly influential in the Pythia-based LA^{4}SR model. This finding was further supported by our motif-based analyses (Fig. 4), where the most influential three-to-six amino residue motifs were usually found 29-34 residues into the sampled sequence.

(F-G) PCA plots from layer-aggregated transformer outputs, corresponding to the main text Fig. 7. Color scales represent attributions.

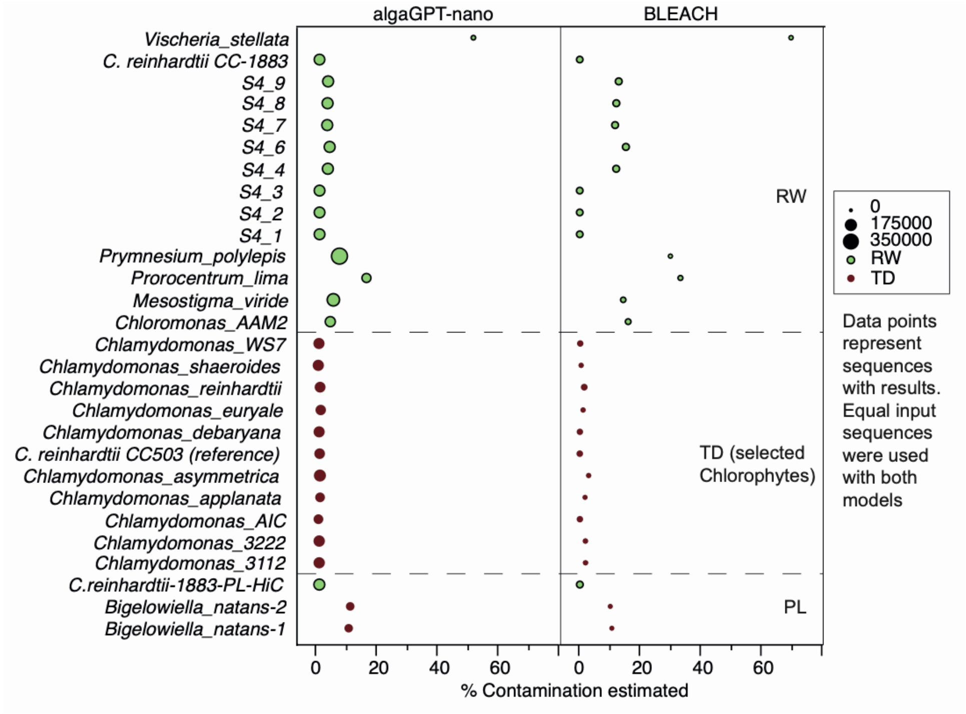

Figure S7. LA^{4}SR models perform well on new, real-world data

Shown are algaGPT-nano and BLEACH (BLAST-based contamination removal method)[S2] estimates of contamination in selected real-world (RW) sequencing, training datasets (TD), and platinum-level (PL) assemblies (i.e., chromosome-level, high coverage of long reads). Sequences known to be contaminated (e.g., the *Vischeria* and *Prorocentrum* assemblies) were compared with clean assemblies (e.g., assemblies from various species of *Chlamydomonas*[S3] and *Bigelowiella)*[S3,S4] from the TD using BLEACH and algaGPT-nano. The Dovetail Hi-C *C. reinhardtii* 1883 and *Bigelowiella natans*[S4] assemblies are shown as platinum-level, *bona fide* axenic reference benchmarks.

SUPPLEMENTARY TABLES

Table S1 | External spreadsheet. This spreadsheet contains LA^{4}SR performance metrics, technical performance estimations, and preliminary BLAST screening results of genomes comprising the algal training data.

Table S2 | External spreadsheet. Captum attributions for 100 sequences each of algal and bacterial origin obtained using the LayerIntegratedGradients function.

Table S3 | External spreadsheet. Influential motifs found with the DeepMotifMinerPro (DMMP) software introduced in this work as well as their functional associations (e.g., PFAM and REACTOME annotations (see also Data S3)).

Table S4 | External spreadsheet. LA^{4}SR and Diamond BLAST[S1] results for data from new assemblies from seen species (Fig. S7), contaminated assemblies from unseen genera (Fig. S7), and clean assemblies from unseen genera (Fig. 8). For the LA^{4}SR results for genome assemblies from unseen genera, the genomes were published after the model was trained, and the genera shown were not included in the training dataset. Newly sequenced genomes were uploaded to NCBI SRA accession SUB14799921.

SUPPLEMENTARY DATASETS

Data S1 | Neural network training sequences (4GB). Dataset comprising DNA sequences processed in two formats: with terminal information (TI-inclusive) and terminal information-free (TI-free).

Data S2 | Training and inference scripts. The LA^{4}SR framework integrated several open-source software packages and models. We used LORA[S5] (Low-Rank Adaptation) and QLORA[S6] (Quantized Low-Rank Adaptation) for parameter-efficient post-training and used Mamba[S7,S8] as an alternative to transformer-based architectures. The Hugging Face Transformers library facilitated implementation, pretraining, and post-training of the open-source models. Training was performed on an HPC cluster, with jobs going to nodes with NVIDIA (Santa Clara, CA, USA) V100, A100, or H100 GPUs.

Data S3 | Interpretability software developed in this study. Includes scripts for the implementation of the custom explainer programs presented with this work, including Captum[S9]-, DeepLift[S10], and SHAP[S11]-based approaches (Data S3) to explain how different amino acid residues and their patterns and positions affect model decisions.

Data S4 | Additional validations. To validate our approach on new clean assemblies from seen taxa, contaminated assemblies from unseen taxa, and new clean assemblies from unseen taxa, we performed additional validations. We applied LA^{4}SR models to data from new assemblies from seen species (Fig. S7), contaminated assemblies from unseen genera (Fig. S7), and clean assemblies from unseen genera (Fig. 8). We downloaded 12 new genomes from NCBI that had been published after the model was trained or otherwise not included in the training data; these genomes constitute the clean, unseen genera (Fig. 8). These unseen genera included two macroalgal genomes (Asparagopsis taxiformis and *Agarophyton chilense*) and one alga lacking chlorophyll (*Prototheca cutis*).

We cultured and sequenced ten separate isogenic colonies of *Chlamydomonas reinhardtii* CC-1883. Of these, nine were sequenced with Illumina 150 bp paired-end short reads and one with Pacific Biosciences (PacBio, Menlo Park, CA, USA) HiFi reads and DoveTail (Sydney, Australia) Hi-C to generate a complete, axenic reference assembly (Fig. S3; Data S4).

Data S5 | Singularity container to run LA^{4}SR. Includes an environment supporting LA^{4}SR, with over 200 dependency packages installed and organized, and run scripts, base programs, and example data inputs and outputs.

SI BIBLIOGRAPHY

S1. Buchfink, B., Xie, C., and Huson, D.H. (2015). Fast and sensitive protein alignment using diamond. Nat Methods. *12*, 59-60. https://doi.org/10.1038/nmeth.3176.

S2. Nelson, D.R., Mystikou, A., Jaiswal, A., Rad-Menendez, C., Preston, M.J., De Boever, F., El Assal, D.C., Daakour, S., Lomas, M.W., Twizere, J.C., et al. (2024). Macroalgal deep genomics illuminate multiple paths to aquatic, photosynthetic multicellularity. Mol Plant. *17*, 747-771. https://doi.org/10.1016/j.molp.2024.03.011.

S3. Nelson, D.R., Hazzouri, K.M., Lauersen, K.J., Jaiswal, A., Chaiboonchoe, A., Mystikou, A., Fu, W., Daakour, S., Dohai, B., Alzahmi, A., et al. (2021). Large-scale genome sequencing reveals the driving forces of viruses in microalgal evolution. Cell Host Microbe. *29*, 250-266 e258. https://doi.org/10.1016/j.chom.2020.12.005.

S4. Curtis, B.A., Tanifuji, G., Burki, F., Gruber, A., Irimia, M., Maruyama, S., Arias, M.C., Ball, S.G., Gile, G.H., Hirakawa, Y., et al. (2012). Algal genomes reveal evolutionary mosaicism and the fate of nucleomorphs. Nature. *492*, 59-65. https://doi.org/10.1038/nature11681.

S5. Hu, E.J., Shen, Y., Wallis, P., Allen-Zhu, Z., Li, Y., Wang, S., Wang, L., and Chen, W. (2021). Lora: Low-rank adaptation of large language models. Preprint at arXiv, https://doi.org/10.48550/arXiv.2106.09685.

S6. Dettmers, T., Pagnoni, A., Holtzman, A., and Zettlemoyer, L. (2023). Qlora: Efficient finetuning of quantized llms. Preprint at arXiv, https://doi.org/10.48550/arXiv.2305.14314.

S7. Dao, T., and Gu, A. (2024). Transformers are ssms: Generalized models and efficient algorithms through structured state space duality. Preprint at arXiv, https://doi.org/10.48550/arXiv.2405.21060.

S8. Gu, A., and Dao, T. (2023). Mamba: Linear-time sequence modeling with selective state spaces. Preprint at arXiv, https://doi.org/10.48550/arXiv.2312.00752.

S9. Kokhlikyan, N., Miglani, V., Martin, M., Wang, E., Alsallakh, B., Reynolds, J., Melnikov, A., Kliushkina, N., Araya, C., Yan, S., and Reblitz-Richardson, O. (2020). Captum: A unified and generic model interpretability library for pytorch. Preprint at arXiv, https://doi.org/10.48550/arXiv.2009.07896.

S10. Shrikumar, A., Greenside, P., and Kundaje, A. (2017). Learning important features through propagating activation differences. Preprint at arXiv, https://doi.org/10.48550/arXiv.1704.02685.

S11. Contreras, J., Winterfeld, A., Popp, J., and Bocklitz, T. (2024). Spectral zones-based shap/lime: Enhancing interpretability in spectral deep learning models through grouped feature analysis. Anal Chem. *96*, 15588-15597. https://doi.org/10.1021/acs.analchem.4c02329.
